# Brain Pathogenesis and Potential Therapeutic Strategies in Myotonic Dystrophy Type 1

**DOI:** 10.3389/fnagi.2021.755392

**Published:** 2021-11-15

**Authors:** Jie Liu, Zhen-Ni Guo, Xiu-Li Yan, Yi Yang, Shuo Huang

**Affiliations:** ^1^Department of Neurology, Stroke Center & Clinical Trial and Research Center for Stroke, The First Hospital of Jilin University, Changchun, China; ^2^China National Comprehensive Stroke Center, Changchun, China; ^3^Jilin Provincial Key Laboratory of Cerebrovascular Disease, Changchun, China

**Keywords:** myotonic dystrophy type 1, central nervous system, pathology, mechanism, treatment

## Abstract

Myotonic dystrophy type 1 (DM1) is the most common muscular dystrophy that affects multiple systems including the muscle and heart. The mutant CTG expansion at the 3′-UTR of the *DMPK* gene causes the expression of toxic RNA that aggregate as nuclear foci. The foci then interfere with RNA-binding proteins, affecting hundreds of mis-spliced effector genes, leading to aberrant alternative splicing and loss of effector gene product functions, ultimately resulting in systemic disorders. In recent years, increasing clinical, imaging, and pathological evidence have indicated that DM1, though to a lesser extent, could also be recognized as true brain diseases, with more and more researchers dedicating to develop novel therapeutic tools dealing with it. In this review, we summarize the current advances in the pathogenesis and pathology of central nervous system (CNS) deficits in DM1, intervention measures currently being investigated are also highlighted, aiming to promote novel and cutting-edge therapeutic investigations.

## Introduction

Myotonic dystrophies (DM1 and DM2) are inherited autosomal dominant skeletal muscle diseases that are characterized by progressive muscle weakness and myotonia, and involves multisystem engagement. DM1, also known as Steinert disease, is caused by the abnormal expansion of a CTG-trinucleotide repeat in the 3′-UTR of the dystrophia myotonic protein kinase (*DMPK*) gene (Brook et al., 1992; Fu et al., 1992; Mahadevan et al., 1992). In healthy individuals, there are approximately 5–38 CTG repeats in the *DMPK* gene, while DM1 patients harbor 50 to several thousands of repeats. CTG repeats tend to increase throughout aging. And as the number of repeats increase, disease severity escalates and age of onset decreases (Bird, 1993). As a progressively debilitating disease, DM1 tends to have an earlier onset and more severe phenotype from one generation to the next, while symptoms could be highly variable among patients (Udd and Krahe, 2012).

Conventionally, DM1 can be divided into five types: congenital, childhood-onset, juvenile-onset, adult-onset, and late-onset, with the adult-onset form being the most prevalent (De Antonio et al., 2016). In the past few years, several neurological symptoms, including cognitive impairment, behavioral impairment, and sensory-motor neural integration have attracted more and more attention, indicating cerebral involvement (Meola and Sansone, 2007; Meola and Cardani, 2015). These central nervous system (CNS) deficits among DM1 individuals significantly increase the disease burden, not only affecting neuropsychological domains but also decreasing the whole quality of life. A large clinical longitudinal study of DM1 patients by Miller et al. (2021) demonstrated that cognitive deficits, hypersomnolence, and apathy are critical brain symptoms of adult-onset DM1 caused by the underlying molecular mechanisms. In contrast, depression and anxiety are secondary coping symptoms with chronic physical and emotional stress (Miller et al., 2021). In addition, Simoncini et al. (2020) proved that the severity and progression rate of neurological impairments are highly variable over time, possibly attributed to underlying neuropathology (Mazzoli et al., 2020). The cognitive impairment in DM1 patients can be severe. This varies significantly with the phenotype, including the typical intellectual disability and lower intelligence quotient (IQ) levels in the congenital phenotype (Angeard et al., 2007, 2011; Echenne et al., 2008; Ekström et al., 2008; Douniol et al., 2012), the reading and spelling impairment, autistic behavior, attention deficits, deficiency in the speed of processing and severe difficulties in social interactions in childhood-onset phenotype (Steyaert et al., 1997), and dysfunctional personality, visuospatial deficits, unawareness of disease symptoms and signs, impaired facial expression and emotion recognition, and later apathy in the adult-onset phenotype (Meola et al., 2003; Winblad et al., 2006; Sansone et al., 2007; Takeda et al., 2009; Kobayakawa et al., 2010, 2012; Sistiaga et al., 2010; Jean et al., 2014; Gallais et al., 2015). Compared with the congenital groups, the childhood group shown greater cognitive and adaptive development (Lindeblad et al., 2019). Fatigue and sleep disorders including excessive daytime sleepiness (EDS) are also prominent complaints in DM1 patients (Steyaert et al., 1997; Quera Salva et al., 2006). Since fatigue involves both central and peripheral performances, the sense of tiredness is primarily caused by CNS dysfunction (Angelini and Tasca, 2012). Till now, a variety of neuropsychological tests have been developed to evaluate the CNS involvement in DM1 (Okkersen et al., 2017a; Simoncini et al., 2020). Tables 1, 2 briefly summarizes the critical CNS symptoms in DM1 and the recent findings about CNS involvement measured by neuropsychological tests in DM1, respectively (Meola and Sansone, 2007; Subramony et al., 2020). In addition, multiple neuroimaging studies relying on structural and functional explorations suggest a wide range of brain abnormalities in DM1 (Okkersen et al., 2017b; Minnerop et al., 2018; Angelini and Pinzan, 2019), mainly involving white matter (WM) abnormalities, widespread gray matter (GM) atrophy and hypometabolism in the frontal lobes. The primary studies focused on neuroimaging abnormalities in DM1 have been shown in Tables 3, 4. Furthermore, specific patterns of neuroimaging alterations and their correlations with other clinical parameters, such as clinical performances and neuropsychological test results have also been discovered, such as sleepiness might be associated with WM status in the superior longitudinal fasciculus and cingulum (Wozniak et al., 2014), and visuospatial impairment might be correlated with WM abnormalities and cortical atrophy (Cabada et al., 2017). Although the single structural or functional alterations seems to be critical for specific CNS dysfunctions, recent advance further revealed the abnormal functional connectivity patterns in DM1 brain and their effects on different personality traits (Serra et al., 2014, 2016a,b, 2020b). Besides, the main executive dysfunction and memory and visuo-spatial impairment were associated with the whole brain volume loss, but cannot be attributed to focal atrophy in any specific regions (Baldanzi et al., 2016b). Small but extensive WM damages in DM1 patients with normal-appearing WM beyond the signal changes detected with conventional MR imaging might be associated with the neuropsychological deficit (Anderson, 2013; Baldanzi et al., 2016b). This implying the critical effects of disrupted complex neuronal networks on cognitive impairments in DM1. Explorations on the complex neuronal networks and undetected lesions and their complex mechanisms contributing to clinical impairments might provide effective outcome measurements as well as effective therapeutic targets for DM1 CNS deficits. Despite a large amount of imaging and neuropsychological evidence of CNS involvement in DM1, the molecular mechanisms driving these deficits are largely unidentified, and targeted therapies aimed at ameliorating neurological deficits are scarce. This review summarizes the recent advances in the pathology and pathogenesis of CNS disorders in DM1 and the promising therapeutic strategies, hoping to provide new proposals for future investigations.

**TABLE 1:** CNS symptom in DM1.

**CNS symptom**	**Related symptoms**
Cognitive change	Mental retardation Reduced IQ values MMSE scores decrease Memory deficits Visual-Spatial deficits Attentional deficits Speech and language delay and verbal memory deficits Impaired facial expression and emotion recognition Difficulty in social communication Brain fog
Behavioral abnormality	Executive dysfunction/aphasia Avoidance behavior Impulsivity Personality changes Apathy Anxiety Depression Anosognosia
Sleep disorder	EDS SDB Restless legs syndrome and PLMS REM sleep dysregulation Long nighttime sleep
Fatigue	

*DM1, myotonic dystrophy type 1; CNS, central nervous system; IQ, intelligence quotient; MMSE, Mini Mental State Examination; EDS, excessive daytime sleepiness; SDB, sleep disorder breathing; PLMS, periodic limb movements of sleep; REM, rapid eye movement.*

**TABLE 2:** Neuropsychological tests in DM1.

**References**	**Neuropsychological tests (alterations)**	**Participants**	**Main results**
Tremblay et al., 2021	WASI-II (Intellectual abilities); FAB (Executive functioning); LARS (Apathy)	11 patients with DM1	Adults with childhood-onset DM1 present relative dependence in regard to IADLs, the level of which is, at least partially, associated with cognitive impairments
Cabada et al., 2021	Digit Span from TBR, Spatial Span from the WMS-III scale, and Letter-Number Sequencing from the WAIS-IV scale (Working memory); BVRT-Part C, Cubes from TBR (Visuospatial constructional ability); TMT-B (Alternating attention); TAVEC (Verbal memory); Word Accentuation Test-30 (Verbal IQ)	33 patients with DM1	DM1 patients have a significant deterioration in test performance that measures working memory and visuospatial skills, which are significantly associated with white matter lesion load
Lopez-Titla et al., 2021	MMSE and MOCA (Cognitive impairment); 19 CANTAB tests (Cognitive domains of attention, global memory, visual memory, and executive functions)	22 patients with DM1 vs. 22 healthy controls	Patients with DM1 have significant deficits in memory and problem-solving tasks WM integrity degradation at frontal, temporomedial, and parietal lobes could be associated with specific memory impairments in DM1
Woo et al., 2019	Wechsler Adult Intelligence Scale (Intelligence); Rey-Kim memory test (Memory); Executive Intelligence Test (Executive function)	19 patients with DM1	Verbal memory impairment significantly deteriorated in the juvenile-onset DM1 as compared to the adult-onset DM1
Breton et al., 2020	The digit symbol coding subscale of the WAIS-R (Processing speed); CVLT (Learning ability and verbal memory); Ruff 2 & 7 (Sustained and selective attention)	115 patients with adult-onset DM1	DNA methylation at the DMPK gene locus might be a predictor of DM1-related cognitive dysfunction
Romeo et al., 2010a	Raven’s progressive Matrices (Non-verbal intelligence); Stroop and Fluency tests (Frontal executive functions); Wechsler Memory Scale and Corsi’s Block tests (Memory and learning functions); Rey-Osterrieth Complex Figure (Visuo-spatial abilities)	50 patients with DM1 vs. 14 patients with DM2	There is a specific temporo-insular diffuse lesional pattern in DM1 There might be a possible correlation between cognitive impairment and diffuse frontal lesions Brain involvement might be different in DM1 and DM2, and severer CNS changes are observed in DM1
Meola et al., 2003	SCID-II personality scale (Personality); TMT (Serial ordering and alternation); WCST (Concept formation and set shifting); Stroop Test (Attentional control and response inhibition); TLT (Planning and problem solving)	21 patients with moderately severe DM1	Executive dysfunction and avoidant personality trait are associated with hypoperfusion in frontal and parieto-occipital regions of the brain
Sistiaga et al., 2010	WAIS-III (IQ, attention and working memory); WCST (Categorization and cognitive flexibility); Stroop Color and Word Test (Automatic response inhibition); Raven’s Progressive Matrices (Visual deduction, semantic and phonetic verbal fluency); BJLOT (Visuospatial ability); RCF (Visual-motor organization and planning strategies); CalCAP (Maintained attention and simple and complex reaction time); RAVLT (Immediate and delayed memory); MCMI-II (Personality traits and psychopathology)	121 adult patients with DM1 vs. 54 healthy controls	CTG expansion size in DM1 is negatively related to many cognitive and personality deficits The cognitive impairment predominantly affects the frontoparietal lobe
Serra et al., 2014	Clinical interview and Minnesota Multiphasic Personality Inventory-2 (Personality assessment)	27 patients with DM1 vs. 16 matched healthy controls	A continuum of atypical personality profiles ranging from schizotypal personality traits to paranoid personality disorder is discovered in DM1 patients Alterations of functional connectivity of the brain may explain the atypical personality traits observed in DM1 patients
Bertrand et al., 2015	SCL-90-R (Psychological symptoms); NEO-FFI (Personality dimensions); Rosenberg Self-Esteem Scale (Self-esteem); ASIQ (Suicidal ideation); WAIS-R Full Scale IQ (Global intellectual functioning); WAIS-R Information score, WAIS-R Verbal IQ, and Boston Naming Test total score (Language abilities); WAIS-R Picture Completion, WAIS-R Block Design, WAIS-R Performance IQ, Hooper’s test, TVPS subtests, and copy of the Rey-Osterrieth Complex Figure (Non-verbal abilities); WAIS-R Digit Span, Ruff 2 & 7 Speed and Accuracy, Stroop subtests, WAIS-R Similarities, Category and Letter verbal fluency, and Raven’s progressive matrices (Attention/executive functions); CVLT and Rey-Osterrieth Complex Figure immediate recall, delayed recall, and recognition total scores (Memory)	200 patients with DM1	Psychological traits differ across DM1 phenotypes The presence of higher phobic anxiety and lower self-esteem are associated with lower education, a higher number of CTG repeats, more severe muscular impairment, and lower cognitive functioning
Kobayakawa et al., 2012	RMET and faux pas recognition (ToM)	9 patients with adult-onset DM1	Social cognitive impairment in patients with adult-onset DM1 is associated with ToM dysfunction
Baldanzi et al., 2016b	RAVLT-IR, RAVLT-DR, ROCF, Digit Span, and CBT (Immediate memory); TMT-A and TMT-B (Selective attention and cognitive flexibility); Stroop Test (Automatic response inhibition); FAS, FAB, Modified WCST (Frontal and executive functions); ROCF-copy (Spatial organization and visuo-constructional skills)	30 patients with DM1	Disrupted complex neuronal networks can underlie cognitive-behavioral dysfunctions in DM1
Gallais et al., 2015	LARS (Apathy); Mini International Neuropsychiatric Interview (MDE); MMSE (Cognitive impairment); Stroop Test (Processing speed, attentional control, response inhibition); FAB (Executive abilities); KFSS (Fatigue)	38 patients with adult-onset DM1 vs. 19 patients with FSHD vs. 20 matched healthy controls	Apathy is more prevalent in DM1 than in FSHD, which is independent of the psychopathological domain, fatigue, age, and motor disability, but is associated with general cognitive status
Labayru et al., 2018	K-BIT (Overall cognitive functioning); Digit span subtest from the WAIS-III (Attention performance); POFA (Emotion recognition); The Faux Pas test (ToM); TECA (Empathy)	38 patients with DM1 vs. matched healthy controls	DM1 patients don’t manifest specific impairments in ToM, while emotion recognition appears as a core deficit
Serra et al., 2020a	Emotion attribution test, social situations test, moral/conventional distinction test (Social cognition)	30 patients with DM1 vs. 25 healthy controls	Cortical thickness changes in DM1 patients are significantly associated with deficits in social cognition performances
Serra et al., 2016a	RMET and ToM-story test (ToM); WAIS-R (Global cognitive efficiency)	20 patients with DM1 vs. 18 healthy controls	Deficits in ToM are associated with specific patterns of abnormal connectivity between the left inferior temporal and frontocerebellar nodes in DM1 brains
Minnerop et al., 2011	c.I.T.S (Focused attention); c.I.T.I (Interference); TMT A (Psychomotoric speed); TMT B (Attention shift, mental flexibility); DSS (Daytime sleepiness); NeurocogFX (Choice reaction time, interference, verbal memory-recognition, figural memory-recognition); KFSS (Fatigue); PSQI (Sleep quality); Ullanlinna-Narcolepsy Scale (Narcolepsy)	22 patients with DM1 vs. 22 patients with DM2 vs. matched healthy controls	Depression in DM might be a reactive adjustment disorder rather than a direct consequence of structural brain damage DM1 presents with more prominent WM lesions than DM2, with prominent callosal body and limbic system affection. WM changes might dominated the extent of gray matter changes.
Rakocevic-Stojanovic et al., 2014	Hamilton rating scale (Depressive and anxiety); KFSS (Fatigue); DSS (EDS); ACE-R (Global cognitive status); RSPM and the Serbian version of WAIS-R (General intellectual level); RAVLT (Verbal memory); ROCF (Visuoconstructive abilities and visual memory); TMT-A (Speed and attention); WCST and TMT-B (Executive functions); BNT (Phonetic and semantic verbal fluency); CANTAB (Attention, visual memory, executive functions)	22 patients with juvenile-onset DM1 vs. 44 patients with adult-onset DM1	Patients with juvenile-onset DM1 scored lower than adult-onset DM1 patients regarding total INQoL score and all INQoL subdomains, except for myotonia. Different central manifestations strongly influence QoL in patients with both adult-onset DM1 and juvenile-onset DM1
Heatwole et al., 2018	MDHI, INQoL (QoL); SF-36v2 (Health impairment)	52 patients with DM1	The MDHI correlates well with objective metrics that reflect disease severity in DM1 Participants in DM1 clinical studies favored the MDHI over the INQoL and the SF-36v2 in multiple areas of perceived relevance, usability, and responsiveness
Peric et al., 2016	SF-36 (QoL)	84 patients with DM1	QoL improved in DM1 patients during a 5-year period despite the disease progression SF-36 should be used with caution as a patient-reported outcome measure in DM1 clinical trials
Peric et al., 2017b	INQoL (QoL)	67 patients with DM1	INQoL questionnaire scores improved in DM1 patients during a 6-year period INQoL score did not correlate with progression of muscle weakness
Symonds et al., 2017	MDHI (Health status); DM1-Activ, DM1 Activc, LIFE-H (Activities of daily living); INQoL (Health-related quality of life); ESS, DSS, CFS, FSS, FDSS (Sleep and fatigue)	/	Several patient-reported outcome assessments are suited to make valid measurements in DM1 populations
Heatwole et al., 2016	MDHI	70 patients with DM1	MDHI is a valid tool to measure disease burden in DM1 patients
Baldanzi et al., 2016a	TIB (Intellectual functioning); BDI-II, STAI-Y2 (Depressive and anxiety); AES (Apathy); RAVLT-IR, RAVLT-DR, ROCF-DR, ROCF-IR, CBT (Immediate memory); TMT-A and TMT-B (Selective attention and cognitive flexibility); Stroop Test (Automatic response inhibition); FAS, FAB, Modified WCST (Frontal and executive functions); ROCF (Spatial organization and visuo-constructional skills); Raven’s progressive matrices (PM47) (Culture-free abstract reasoning); INQoL (Disease awareness)	65 patients with adult-onset DM1	Several cognitive functions, including executive and mnesic domains with visuo-spatial involvement, were affected in DM1 patients The reduced illness awareness occurs across different physical and life domains, and it appears more prominent in Activities and Independence domains The unawareness significantly related to the cognitive performance deficits, specifically in the domains of visuo-spatial memory, cognitive flexibility and conceptualization

*ACE-R, Addenbrooke’s Cognitive Examination-Revised; AES, Apathy Evaluation Scale; ASIQ, Adult Suicidal Ideation Questionnaire; BDI-II, Beck Depression Inventory-II; BJLOT, Benton Judgment of Line Orientation Test; BNT, Boston naming test; BVRT, Benton Visual Retention Test; CBT, Corsi Block-Tapping test; CFS, Chalder Fatigue Scale; c.I.T.S, subtest (symbol counting) of the Cerebraler Insuffizienztest; c.I.T.I, subtest (response inhibition) of the Cerebraler Insuffizienztest; CVLT, California Verbal Learning Test; CalCAP, California Computerized Assessment Package; CANTAB, Cambridge Neuropsychological Test Automated; DM, myotonic dystrophy; DSS, Daytime Sleepiness Scale; EDS, excessive daytime sleepiness; FDSS, the Fatigue and Daytime Sleepiness Scale; FSHD, facioscapulohumeral dystrophy; FAB, Frontal Assessment Battery; FAS, phonemic verbal fluency test; FSS, the Krupp Fatigue Severity Scale; IADLs, instrumental activities of daily living; INQoL, Individualized Neuromuscular Quality of Life questionnaire; IQ, Intellectual Quotient; KFSS, Krupp’s Fatigue Severity Scale; K-BIT, Kaufman Brief Intelligence Test; LARS, Lille Apathy Rating Scale; LIFE-H, Assessment of Life Habits; MCMI, Millon Multiaxial Clinical Inventory; MDE, major depressive episodes; MDHI, Myotonic Dystrophy Health Index; MMSE, Mini Mental State Evaluation; MOCA, Montreal Cognitive Assessment; NEO-FFI, NEO Five-Factor Inventory; NeurocogFX, computerized neuropsychological screening test battery; POFA, Pictures of Facial Affect; PSQI, Pittsburgh Sleep Quality Index; RCF, Rey’s Complex Figure; QoL, Quality of life; RMET, Reading the Mind in the Eyes Test; RAVLT-IR, Immediate Recall of the Rey Auditory Verbal Learning Test; RAVLT-DR, Delayed Recall of the Rey Auditory Verbal Learning Test; ROCF-IR, Immediate Recall of the Rey-Osterrieth Complex Figure test; ROCF-DR, Delayed Recall of the Rey-Osterrieth Complex Figure test; RSPM, Raven standard progressive matrices; STAI-Y2, State-Trait Anxiety Inventory-2; SCL-90-R, Symptom Checklist-90-Revised; TAVEC, Test de Aprendizaje Verbal España-Complutense; TBR, Test de Barcelona Revised; TECA, Test of Cognitive and Affective Empathy; TIB, Brief Intelligence Test; TLT, Tower of London Test; TMT, Trail-Making Test; ToM, theory of mind; TVPS, Test of Visual-Perceptual Skills; WAIS, Wechsler Adult Intelligence Scale; WAIS-R, Wechsler Adult Intelligence Scale-Revised; WCST, Wisconsin Card Sorting Test.*

**TABLE 3:** Structural brain imaging in DM1.

**References**	**Imaging tools**	**Participants**	**Imaging abnormalities**	**Possible correlations between imaging abnormalities and clinical symptoms**
Walker et al., 1984	CT	22 adults with DM vs. 45 healthy controls	Increased ventricular surface areas Focal cerebral atrophy	
Glantz et al., 1988	0.5T MRI	14 patients with DM vs. 12 controls	Periventricular hyperintensities Ventricular enlargement	
Hashimoto et al., 1995	MRI	7 patients with adult-onset DM vs. 6 patients with CDM	The incidence of a small corpus callosum or ventricular enlargement is higher in CDM than in adult-onset DM	
Baldanzi et al., 2016b	3T MRI BPF VBM LL% and Fazekas scale	30 patients with DM1 vs. healthy controls	Widespread GM reduction Decreased FA and increased RD, MD and AD	Negative relationships are discovered between left temporal atrophy and verbal memory, between RD and mnesic and visuo-spatial cognitive domains, and between AD and verbal memory The involvement of normal appearance WM, beyond the signal changes detected with conventional MR imaging, is associated with neuropsychological deficit
Romeo et al., 2010a	MRI SPECT	50 patients with DM1 vs. 14 patients with DM2 vs. 44 healthy controls	Scattered supratentorial, bilateral, symmetrical focal or diffuse WMHLs A typical temporo-insular diffuse subcortical pattern Minimal hypoperfusion in the posterior cortex planes	
Weber et al., 2010	BPF VBM FDG-PET	20 patients with DM1 vs. 9 patients with DM2 vs. healthy controls	Global GM volume reduction A bilateral hippocampal volume reduction Frontal and parietal lobes volume reduction A frontotemporal hypometabolism	Hippocampal volume reduction is correlated to episodic memory deficits
Kobayakawa et al., 2010	MRI	9 patients with DM1 vs. 13 healthy controls	More severe lesions in the frontal, temporal, and insular white matters	Sensitivity to the emotion of disgust is negatively correlated with temporal lesions Sensitivity to anger is negatively correlated with frontal, temporal, and insular lesions
Minnerop et al., 2011	3T MRI VBM DTI	22 patients with DM1 vs. 22 patients with DM2 vs. 22 healthy controls	Extensive WM changes involved all cerebral lobes, brainstem, corpus callosum and limbic system, especially in frontal WM	Sleepiness is linked with FA values in the brainstem
Wozniak et al., 2013	DTI	16 patients with DM1 vs. 15 healthy controls	Diffusive WM abnormalities	WM abnormalities are associated with the degree of working memory impairment
Wozniak et al., 2011	DTI	8 patients with DM1 vs. 8 healthy controls	Abnormal WM integrity indices: FA, RD, MD, and AD	Whole cerebrum fractional anisotropy is correlated with full-scale intelligence and a measure of executive functioning
Franc et al., 2012	MRI	5 adults with CDM1 vs. 5 adults with adult onset DM1 vs. 5 adults with DM2 vs. 5 healthy controls	WM integrity reduction GM volumes reduction only in adult-onset DM1 patients	
Wozniak et al., 2014	3T MRI DTI	45 patients with DM1 vs. 44 healthy controls	Bilateral disturbances in WM integrity	DTI metrics are correlated with cognitive functioning, particularly working memory and processing speed WM integrity is correlated with the muscular impairment Sleepiness is associated with WM status in the superior longitudinal fasciculus and cingulum
Caso et al., 2014	MRI VBM	51 patients with DM1 vs. 34 healthy controls	WM hyperintensities Regional GM atrophy WM tract microstructural damage	WMHs and microstructural damage are correlated with cognitive deficits
Schneider-Gold et al., 2015	3T MRI VBM	12 patients with juvenile or classical DM1 vs. 16 adults with DM2 vs. 33 healthy controls	Ventricular enlargement Supratentorial GM and WM atrophy WM reduction in the splenium of the corpus callosum and in left-hemispheric WM adjacent to the pre- and post-central gyrus	Morphological changes is related to reduced flexibility of thinking and atrophy of the left secondary visual cortex
Serra et al., 2015	3T-MRI BPF VBM	10 patients with DM1 vs. 16 healthy controls	Widespread GM atrophy and WM integrity	Extent of GM and WM damage is correlated with CTG triplet expansion and cognition
Baldanzi et al., 2016b	3T-MRI BPF VBM	30 patients with DM1 vs. 30 healthy controls	Widespread GM atrophy Decreased FA and increased RD, MD, and AD	BPF value is correlated with visuo-spatial and executive impairment Negative relationship between left temporal atrophy and verbal memory, between RD and amnesic and visuo-spatial cognitive domains, and between AD and verbal memory
Zanigni et al., 2016	DTI MRI VBM	24 adults with DM1 vs. 25 healthy controls	Widespread WM DTI abnormalities GM volume reduction	
Cabada et al., 2017	MRI DTI	42 patients with DM1 vs. 42 healthy controls	WML load Cortical and corpus callosum atrophy Diffuse WM DTI abnormalities	Visuospatial impairment is correlated with WM abnormalities and cortical atrophy Daytime sleepiness is associated with WML and ventral diencephalon and pallidum volume loss
Sugiyama et al., 2017	VBM	28 patients with DM1 vs. 28 healthy controls	Extensive GM atrophy, including cortical and subcortical structures Increased connectivity in the left fusiform gyrus and decreased connectivity in the right striatum	Increased connectivity in the left fusiform gyrus and decreased connectivity in the right striatum are associated with impairment in face perception and theory of mind, and schizotypal-paranoid personality traits
Labayru et al., 2019	MRI DTI VBM	31 patients with DM1 vs. 57 healthy controls	Global GM and WM volume reduction FA reduction	Higher ratings on muscular impairment and longer CTG expansion sizes predict a greater volume decrease in GM and lower FA values
van der Plas et al., 2019	3T MRI	79 patients with DM1 vs. 58 healthy controls	Smaller ICV Smaller volume in frontal GM and WM, parietal GM, corpus callosum, thalamus, putamen, and accumbens Larger volumes of the hippocampus and amygdala	Some morphological differences are associated with cognitive deficits and EDS
Serra et al., 2020a	3T MRI	31 patients with DM1 vs. 25 healthy controls	Thickness reduction in the right premotor cortex, angular gyrus, precuneus, and inferior parietal lobule	Cortical thickness are associated with social cognition performances
Cabada et al., 2020	MRI VBM DTI	33 patients with DM1	Increased WML Ventricular enlargement Decreased volume of the left thalamus, caudates, putamen, and hippocampus Global cortical volume decrease Mean diffusivity increase and fractional anisotropy decrease in WM	Working memory and visuospatial skills deterioration are significantly associated with WML load and mean diffusivity increase Progressive WM and GM involvement
Langbehn et al., 2021	3T MRI	59 patients with adult-onset DM1 vs. 68 healthy controls	Pathological increased volume of the hippocampus	Enlarged hippocampal volume is inversely associated with cognitive dysfunction
Leddy et al., 2021	Conventional and qMT MRI	28 patients with DM1 vs. 29 patients with MS vs. 15 healthy controls	Higher prevalence of anterior temporal lobe lesions, but none in the cerebellum and brainstem Characteristic demyelination (significantly reduced F values) A similar WM lesion distribution compare with that typical of relapsing remitting MS	

*DM, myotonic dystrophy; MRI, magnetic resonance imaging; CDM, congenital DM; SPECT, single photon emission computed tomography; GM, gray matter; WM, white matter; WMHL, WM hyperintense lesions; BPF, brain parenchymal fraction; VBM, voxel-based morphometry; FDG-PET, fluorodeoxyglucose positron emission tomography; DTI, diffusion tensor imaging; FA, fractional anisotropy; RD, radial diffusivity; MD, mean diffusivity; AD, axial diffusivity; WMH, WM hyperintensities; WML, WM lesion; ICV, intracranial volume; EDS, excessive daytime somnolence; qMT, quantitative magnetization transfer; MS, multiple sclerosis.*

**TABLE 4:** Functional brain imaging in DM1.

**References**	**Imaging tools**	**Participants**	**Imaging abnormalities**	**Possible correlations between imaging abnormalities and clinical symptoms**
Toth et al., 2015	fMRI	8 DM1 patients with grip myotonia vs. 8 DM1 patients without grip myotonia		Myotonia is related to cortical function in high-order motor control areas
Serra et al., 2014	RS-fMRI	27 patients with DM1 vs. 16 health controls		DMN functional connectivity in the bilateral posterior cingulate and left parietal nodes are associated with schizotypal-paranoid traits
Serra et al., 2016a	RS-fMRI	20 patients with DM1 vs. 18 healthy controls	Specific patterns of abnormal connectivity between the left inferior temporal and fronto-cerebellar nodes	Specific patterns of abnormal connectivity are associated with atypical personality profiles and ToM deficits
Serra et al., 2016b	RS-fMRI	31 patients with DM1 vs. 26 healthy controls	Reduced connectivity in a large frontoparietal network Peculiar patterns of frontal disconnection Increased parietal-cerebellar connectivity	Reduced connectivity in a large frontoparietal network is correlated with isolated impairment in visuospatial reasoning The balance between loss of connectivity and compensatory mechanisms is correlated with the paradoxical mismatch between structural brain damage and minimal cognitive deficits
Serra et al., 2020b	RS-fMRI	32 patients with DM1 vs. 26 healthy controls	Increased functional connectivity between VTA and the left supramarginal and superior temporal gyri	Deficit of decision-making is related to increased connectivity between VTA and brain areas critically involved in the reward/punishment system and social cognition
Renard et al., 2016	FDG-PET	24 patients with DM1 vs. 24 healthy controls	Reduced FDG-uptake especially in Brodmann area 8	Reduced FDG-uptake in Brodmann area 8 is correlated to CTG-repeat numbers
Peric et al., 2017a	FDG-PET	16 patients with DM1 vs. 13 patients with DM2	Prominent glucose hypometabolism in prefrontal, temporal, and pericentral regions	Right frontotemporal hypometabolism is associated with executive dysfunction
Romeo et al., 2010b	PET/SPECT	58 patients with DM1 subjected to SPECT and 17 patients with DM1 subjected to PET	Reduced CBF and perfusion and abnormal glucose metabolism, more pronounced in the left hemisphere, the frontal lobe and the cortex	
Meola et al., 2003	SPECT	21 patients with moderately severe DM1	Frontal and parieto-occipital hypoperfusion	Specific cognitive and behavioral profile (avoidant trait personality disorder) is associated with hypoperfusion in frontal and parieto-occipital regions of the brain
Chang et al., 1998	MRS	14 patients with DM vs. 24 healthy controls	Elevated levels of myoinositol, total creatine, and choline-containing compounds	Creatine and myoinositol levels are proportional to the number of trinucleotide (CTG)n repeats
Akiguchi et al., 1999	MRS	21 patients with DM vs. 16 healthy controls	Lower ratio of N-acetylaspartate to creatine and phosphocreatine Lower ratio of N-acetylaspartate to choline-containing compounds	
Krogias et al., 2015	TCS	17 patients with DM1 vs. 14 patients with DM2 vs. 31 healthy controls	Third ventricle enlargement	Mesencephalic raphe echogenicity is related with EDS
Peric et al., 2014b	TCS	66 patients with DM1 vs. 55 health controls	Increased third ventricle width Brainstem raphe hypoechogenicity Substantia nigra both hypoechogenicity and hyperechogenicity	

*DM, myotonic dystrophy; fMRI, functional MRI; RS-fMRI, resting-state fMRI; ToM, theory of mind; VTA, ventral tegmental area; FDG-PET, fluorodeoxyglucose positron emission tomography; MRS, magnetic resonance spectroscopy; TCS, transcranial sonography.*

## Molecular Mechanisms

**FIGURE 1:**
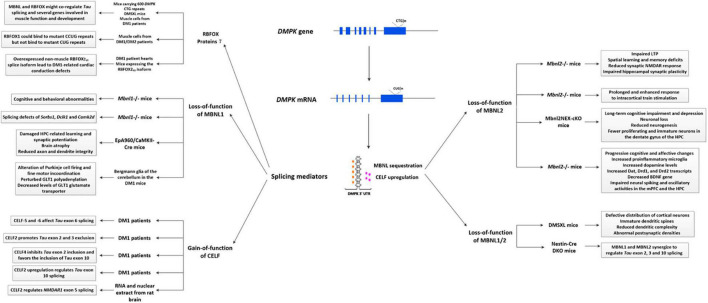
Primary splicing defects described in the DM1 brain. Splicing defects are generally considered the primary cause of DM1 pathology, similar to those in the brain. Specifically, the mutant *DMPK* gene transcripts into expanded CUG RNA, which folds into a hairpin-like structure in the nucleus called “nuclear foci.” These nuclear foci then bind and regulate RNA-binding proteins, affecting alternative splicing, eventually causing a wide range of pathogenic changes. Most splicing defects involved in brain pathology are mediated by the sequestration of MBNL proteins and upregulation of CELF proteins. In animal studies, it has been shown that loss-of-function of MBNL1 might be related to cognitive and behavioral abnormalities, learning abnormalities, alteration of Purkinje cell firing and fine motor incoordination, and perturbed GLT1 levels. Besides, it also contributes to alternative splicing alterations of several genes, such as *Sorbs1*, *Dclk1*, and *Camk2d*. Loss-of-function of MBNL2 could be associated with sleep alterations, spatial learning and memory deficits, impaired synaptic NMDAR response and hippocampal synaptic plasticity, neuronal loss, reduced neurogenesis, cognitive and affective changes and so on. Meanwhile, synergistic effects of MBNL1 and MBNL2 can regulate *Tau* exon 2, 3, and 10 splicing, while a combination loss of MBNL1/2 might be related to abnormal sleepiness, defective distribution of cortical neurons, immature dendritic spines, reduced dendritic complexity, and abnormal PSDs. In DM1 patients, it has been shown that gain-of-function of several CELF proteins can regulate *Tau* exon 2, 3, 6, and 10 splicing, and CELF2 can regulate *NMDAR1* exon 5 splicing. A novel splicing regulator, RBFOX protein has recently been considered a new player in DM1 pathogenesis, *via* a competitive or cooperative interaction with MBNL1. Additionally, the non-muscle RBFOX2_40_ splice isoform in DM1 heart may also contribute to cardiac conduction defects. DM1, myotonic dystrophy type 1; CELF, CUGBP/Elav-like family; MBNL, muscleblind-like; GLT1, glutamate transporter 1; LTP, long-term potentiation; mPFC, medial prefrontal cortex; HPC, hippocampus; BNDF, brain derived-neurotrophic factor.

The pathogenesis of DM1 has been mainly attributed to the gain-of-function of toxic RNA (Pettersson et al., 2015). Specifically, the mutant CTG repeat at the 3′-UTR of the *DMPK* gene causes toxic RNA expression that accumulate in the nucleus called “nuclear foci,” which interferes with RNA-binding proteins (Miller et al., 2000; Fardaei et al., 2002), leading to the sequestration of muscleblind-like (MBNL) proteins and upregulation of CUGBP/Elav-like family (CELF) proteins. These alterations subsequently affected hundreds of mis-spliced effector genes, resulting in aberrant expression of embryonic splice isoforms and loss of these gene product functions, accounting for the multisystemic phenotype (Udd and Krahe, 2012; Chau and Kalsotra, 2015). In recent years, other factors, such as repeat-associated non-AUG (RAN) translation, which can contribute to the formation of toxic homopolymeric (e.g., polyQ) polypeptides, aberrant polyadenylation, activation of protein kinase C (PKC)-dependent signaling pathway, and microRNA deregulation have also been reported to play important roles in DM1 (Chau and Kalsotra, 2015). Regarding CNS, alternative splicing dysregulation has been frequently reported (Caillet-Boudin et al., 2014). Meanwhile, the widespread distributions of mutant *DMPK* mRNA accumulated in nuclear foci in neurons, astrocytes, oligodendrocytes, as well as in human DM1 induced pluripotent stem cell (iPSC)-derived neural stem cells (NSCs) have been reported (Jiang et al., 2004; Hernández-Hernández et al., 2013a; Xia et al., 2013; Sicot et al., 2017). In animal models, different splicing defects and their associated CNS symptoms were also studied (Figure 1). Except for these, emerging pathogenic events independent of splicing defects have also been discovered in the brain of DM1 patients (Marteyn et al., 2011; Hernández-Hernández et al., 2013b). Some CNS symptoms are non-linearly dependent on patient age and CTG repeat length, suggesting the complex and multifactorial mechanisms driving neurological deficits (Heatwole et al., 2012). This section concludes the current advance about CNS pathogenesis, hoping to better understand the complex nature of the DM1 CNS disorders.

### Loss Function of Muscleblind-Like Proteins

Three MBNL paralogs are expressed in mammals, MBNL1, MBNL2, and MBNL3, which can be involved in regulating alternative splicing, mRNA stability, translation and so on (Charizanis et al., 2012; Wang et al., 2012). A large amount of previous studies have revealed the critical role of MBNL proteins in muscle or heart-related disorders, while in recent years, a close relationship between the loss-of-function of MBNL and DM1 brain-related phenotypes were also detected (Matynia et al., 2010; Charizanis et al., 2012; Goodwin et al., 2015), especially MBNL2. Nowadays, hundreds of dysregulated splicing factors, including in *Cacna1d* (McKinney et al., 2009), *Tanc2* (Han et al., 2010), *Ndrg4* (Yamamoto et al., 2011), and *GRIN1* (Shimizu et al., 2000) have been detected in the brain of *Mbnl* 2–/– mice, most of which were similarly dysregulated in DM1 patients, indicating a critical role of the *Mbnl2* loss in DM1 brain pathology (Charizanis et al., 2012). Further, *Mbnl2*–/– mice which are exposed to sleep deprivation also developed several CNS features including impaired long-term potentiation (LTP), deficits in spatial memory, reduced synaptic NMDAR response and impaired hippocampal synaptic plasticity (Charizanis et al., 2012), which were similar to the DM1 phenotype. In 2018, one study described prolonged and enhanced responsiveness to intracortical train stimulation in *Mbnl2*–/– mice, partially attributed to abnormal glutamate neurotransmission (Chen et al., 2018). Particularly, these abnormalities have also been observed in DM1 patients (Meola and Sansone, 2007; Takado et al., 2015).

Several studies also reported a potential role of MBNL1 protein in CNS disorders. For example, cognitive and behavioral abnormalities have been discovered in *Mbnl1*–/– mice (Matynia et al., 2010). In cultured primary hippocampal neurons and EpA960/CaMKII-Cre mice (a brain-specific DM1 model carrying 960 *DMPK* CTG repeats in the postnatal brain), expanded CUG repeats led to deubiquitination of cytoplasmic MBNL1, subsequent nuclear translocation, and morphological defects. These effects can be ameliorated by inhibiting the degradation of lysine 63-linked polyubiquitin chains or by promoting MBNL1 ubiquitination (Wang P. Y. et al., 2018). In 2020, cell studies shown that gain-of-function of MBNL1 could reverse the proliferation defect of skeletal muscle satellite cells in DM1 by inhibiting autophagy *via* the mTOR pathway (Song et al., 2020). Moreover, functional characterization of neuronal cells derived from human embryonic stem (ES) cells reported a reduced proliferative capacity and increased autophagy associated with alterations of the mTOR signaling pathway, while gain-of-function of MBNL1 rescued the phenotype (Denis et al., 2013), suggesting that MBNL1 loss might influence brain pathology by regulating the mTOR signaling pathway. Some important splicing defects have also been detected in the brain of *Mbnl1*–/– mice, in genes such as *Sorbs1*, *Dclk1*, and *Camk2d* (Suenaga et al., 2012). However, the extent of alternative splicing defects in the brain of *Mbnl1*–/– mice was much less than that observed in DM1. A number of alternative exons, such as *GRIN1* exon 4, *APP* exon 7, and *Tau* exons 3 and 9, which have already been reported to be mis-spliced in the brains of DM1 patients, were unaltered in *Mbnl1*–/– mice, thus indicating a limited contribution of MBNL1 to DM1 CNS defects (Suenaga et al., 2012).

In addition, it’s worth noting that the combined loss of MBNL1 and MBNL2 has shown infant/immature structural phenotypes in mutant brains, similar to that of DM1 patients (Goodwin et al., 2015). Besides, single gene knockout may also contribute to the compensatory upregulation of the remaining *Mbnl* genes (Lee et al., 2013). In *Mbnl1*–/– mice, the expression of MBNL2 could be upregulated, which subsequently targets transcripts that are normally regulated by MBNL1 (Lee et al., 2013). These findings indicate a collaborative role of MBNL1 and MBNL2 involved in DM1 CNS.

### Gain of CUGBP/Elav-Like Family Activities

The human CELF family has six members, all of which are involved in alternative splicing regulation (Ladd et al., 2001, 2004). Among these, the upregulations of CELF1 and CELF2 have been observed in the brain of DM1 patients (Dhaenens et al., 2011).

CELF1 has been identified as a (CUG)n repeat-binding protein (Timchenko et al., 1996). Unlike MBNL1, it is increased in DM1 patients mainly through PKC-mediated phosphorylation to stabilize the protein (Kuyumcu-Martinez et al., 2007), or through decreased levels of miR-23a/b (Kalsotra et al., 2014). In opposite, the increasing expression of CELF2 is not related to protein hyperphosphorylation, indicating other potential regulatory mechanisms. CELF1 and CELF2 can influence various transcripts (Ladd, 2013) in the DM1 brain, such as different exons of *Tau* and *NMDAR1* exon 5 (Leroy et al., 2006a,b). Tau proteins can promote neurite outgrowth, organize axonal microtubules, and participate in kinesin-dependent axonal transport (Andreadis, 2012). NMDARs are key components of glutamate-mediated excitatory signaling, which can contribute to excitatory synaptic transmission and synaptic plasticity, thought to be the basis of learning and memory (Zorumski and Izumi, 2012). It has been discovered that four exons (2, 3, 6, and 10) of *Tau* isoforms could respond to one or more CELF proteins in DM1 (Leroy et al., 2006a,b; Dhaenens et al., 2011). CELF2 can regulate alternative exon 5 transcripts of *NMDAR1* to change neuronal excitation in rat brain (Zhang et al., 2002), while CELF1 is inefficient. Aside from alternative splicing regulation, CELF proteins can also participate in regulating mRNA adenylation status, stability, and translation in various cell types (Dasgupta and Ladd, 2012), indicating its potential cytoplasmic functions in the brain.

### A Novel Splicing Regulator—RBFOX Proteins

RBFOX proteins are sequence-specific RNA binding proteins which can regulate alternative splicing in multiple tissues, such as skeletal muscle, heart, and brain (Gehman et al., 2011; Singh et al., 2014; Conboy, 2017; Jacko et al., 2018). Nowadays, it has been discovered that RBFOX1 is involved in the regulation of synapses and autism-related genes in the cytoplasm of neurons (Wang et al., 2016). Klinck et al. (2014) reported that MBNL1 and RBFOX1 protein could co-regulate the splicing of a series genes involved in muscle function and development, some of which are also mis-spliced in DM1 tissues. And the decreased RBFOX1 may amplify the mis-splicing changes caused by the loss-of-function of MBNL1. At the same time, they found that the ectopic expression of RBFOX1 partially rescued the mis-splicing of *Tau* exon 2 in glioblastoma cells. Since the MBNL proteins have been shown to regulate the splicing of *Tau* exon 2 in DM1 brains, it is therefore interesting to postulate that cooperation of MBNL and RBFOX1 might regulate *Tau* splicing in DM1 brains. Nevertheless, Sellier’s researches proposed different results, that RBFOX1 could bind to expanded CUG RNA repeats, competing with MBNL1 and reducing the sequestration of MBNL1 in DM2 muscle cells, which suggest a partly competitive relationship between RBFOX1 and MBNL1 (Sellier et al., 2018). Misra et al. (2020) found a new non-muscle RBFOX2_40_ splice isoform which is overexpressed in DM1 patient hearts. Particularly, mice expressing the RBFOX2_40_ isoform in hearts also performed DM1-related cardiac conduction dysfunctions (Misra et al., 2020), which may due to its promotion of the production of pathogenic ion channel splice variants. All of these results provide a novel idea explaining the splicing dysregulation in DM1, though their possible roles in CNS dysfunctions still need further exploration.

### Effector Genes Alterations Due to Splicing Defects

Nowadays, hundreds of spliced effector genes changes have been discovered in the DM1 brain (Jiang et al., 2004; de León and Cisneros, 2008; Suenaga et al., 2012; Hernández-Hernández et al., 2013a; Otero et al., 2021). Jiang et al. (2004) screened 45 exons (in 31 genes) spliced in the brain of DM1 patients, wherein four of them changed in the ratio of exon inclusion/exclusion splice products, including decreased inclusion of *APP* exon 7, *Tau* exons 2 and 10, and increased inclusion of *NMDAR1* receptor exon 5. Interestingly, the sequences encoding *APP* exons 7 and 8 are excluded in neurons, but are included in astrocytes, indicating the splicing defects in astrocytes. In 2021, *via* detecting transcriptome alterations in frontal cortex of DM autopsy samples, Otero et al. (2021) reported 130 high-confidence splicing changes, which occur in ion channels, neurotransmitter receptors, and synaptic scaffolds, while mis-splicing of *GRIP1* might change kinesin association. In frontal cortex samples, downregulated genes tend to express in neurons, while upregulated genes tended to express preferentially in endothelial and microglial, which suggest neuroinflammatory responses. In DMSXL mice (carrying ∼1,000–1,800 *DMPK* CTG repeats with multisystemic transgene expression), the mis-splicing patterns of *GRIN1* exon 21, *Ldb3* exon 11, and *Mbnl2* exon 7 in frontal cortex, as well as *GRIN1* exon 5, *Ldb3* exon 11, *APP* exon 8, and *Frx1* exons 15/16 in brainstem have been detected (Hernández-Hernández et al., 2013a), and *GRIN1* and *Tau* mis-splicing appear to be involved in synaptic dysfunction. In the brain of *Mbnl1*–/– mice, 14 mis-spliced events have been observed using splicing-sensitive microarray, including *Sorbs1* exons 6 and 25, *Spag9* exon 31, *Dclk1* exon 19, *APP* exon 7 and 8, *GRIN1* exon 4, and so on (Suenaga et al., 2012).

### Heterogeneity in Splicing Defects in Different Brain Regions

Recently, the heterogeneity of splicing defects in the DM1 brain also attracted increasing attention. In *Mbnl* knockdown mice, it has been identified that abnormal alternative splicing in the cerebellum are fewer than in other brain regions (Charizanis et al., 2012; Suenaga et al., 2012). The inclusion of *Mbnl1* exon 5 and *Mbnl2* exons 5 and 8 were higher in most brain areas, except in the cerebellum for *Mbnl2* exons 5 and 8. Besides, RNA foci preferentially accumulate in the frontal cortex and certain areas of the brainstem of DM1 transgenic mice (Huguet et al., 2012; Hernández-Hernández et al., 2013a), and seem to be more abundant in cortical astrocytes than in neurons (Hernández-Hernández et al., 2013a). Using autopsied brain tissues of DM1 patients, researchers further observed varying degrees of mis-splicing among the cerebellar cell layers (Furuta et al., 2018). LASER capture microdissection revealed splicing defects in the molecular layer of the cerebellum, but not in the granular layer (Furuta et al., 2018). Similarly, one study reported that mis-splicing in WM is less apparent than in GM of the DM1 brain, which may be attributed to the inability to transfer the abnormal/fetal splicing isoform to the axon (Nishi et al., 2020). Future analysis of the mis-splicing diversity in the DM1 brain may favor a precise therapy targeting specific sites.

### Translational RNA Differences

Though sharing with a common pathogenetic mechanism, alternative splicing defects, further findings suggested a new perspective influencing the performances of DM patients. According to the results of Salvatori et al. (2009) the molecular and biochemical differences of troponin T and the insulin receptor between DM1 and DM2 due to different translational patterns and distribution patterns might partially explain the apparent differences on their clinical phenotypes. This phenomenon promotes the exploration of DM1 pathogenesis at the translation level.

### Somatic Expansion

Recently, an important aspect of the pathogenesis of DM1 regarding to somatic expansion has attracted increasing attention. It has been demonstrated that the length of modal allele in blood DNA samples of DM1 patients increased over time, driven primarily by the inherited progenitor allele length (ePAL), age-at-sampling, and age-at-onset (Morales et al., 2020). Since the DNA mismatch repair proteins MSH2, MSH3, and MSH6, are considered critical players in CTG repeat expansion, and their decreased expressions inhibit the expansion (Dragileva et al., 2009; Tomé et al., 2009), they provide a new insight into the mechanism of CTG repeat instability in DM1. Tomé et al. (2009) reported that MSH2 ATPase domain mutation could influence somatic instability in DM1 transgenic mice. Compared with fibroblasts, MSH2, MSH3, and MSH6, were highly expressed in DM1 patient-derived iPSC, accompanied with longer CTG repeat, while MSH2 silencing inhibited CTG repeat expansion (Du et al., 2013). Flower et al. (2019) reported that the altered MSH3 levels caused by MSH3 3a repeat allele decreased somatic expansion and changed the DM1 phenotype. Thus, regulation of MSH3 might represent a new roadmap for potential target therapy of DM1.

## Cellular Processes Alterations

### Synaptic Dysfunction

Synaptic protein dysfunction is an important feature demonstrated in the DM1 brain. RAB3A is an abundant synaptic vesicle protein that regulates neurotransmission by interacting with other synaptic proteins. The upregulation of RAB3A causes spontaneous exocytosis, and plays important roles in spatial learning, sleep control, and synaptic plasticity (Sudhof, 2004). Synapsin I (SYN1) can regulate synaptic vesicle release in a phosphorylation-dependent manner, and its hyperphosphorylation determines short-term synaptic plasticity alterations (Rosahl et al., 1993). Hernández-Hernández et al. (2013a) reported RAB3A upregulation and SYN1 hyperphosphorylation in DMSXL mice, transfected cells, and DM1 patient samples, which were related to altered spontaneous neurosecretion in cell culture, electrophysiological and behavioral deficits in mice, and possibly contributed to the neuropsychological manifestations in DM1 patients. Then, Jimenez-Marin et al. (2021) demonstrated that transcriptional signatures of synaptic vesicle genes at least partially explain the mechanisms of DM1 neurodegeneration. Nowadays, a close relationship between splicing regulator alterations and synaptic protein dysfunctions has been found, possibly contributing to DM1 neurological phenotypes (Hernández-Hernández et al., 2013b). For example, loss-of-function of MBNL1 in DMSXL mice brain mediated the upregulation of RAB3A levels and determined the length of neuronal dendrites and axons (Hernández-Hernández et al., 2013b). The overexpression of CELF1 or CELF2 in neuronal-like PC12 cells containing expanded CUG transcripts mediated SYN1 hyperphosphorylation (Hernández-Hernández et al., 2013b). And other splicing alterations, such as *GRIN1* and *Tau* splicing defects, might also contribute to synaptic dysfunction in DM1 (Hernández-Hernández et al., 2013b). However, although influenced by different splicing regulators, these synaptic protein alterations seem to be independent of mis-splicing of their coding transcripts, suggesting that DM1 neuropathogenesis have far-reaching implications beyond the disruption of splicing programs.

In addition, other synaptic-related abnormalities have also been identified in the DM1 brain. Lee et al. (2019) found distribution defects of cortical neurons, abnormal dendritic morphology and complexity and postsynaptic densities in the *Mbnl1/2*–/– mice. A time-course study using EpA960/CaMKII-Cre mice revealed that hippocampus (HPC)-related learning and synaptic potentiation were damaged before structural alterations occurred in the brain, followed by brain atrophy associated with progressively reduced axon and dendrite integrity. Notably, cytoplasmic MBNL1 level on dendrites decreased before dendrite degeneration, whereas MBNL2 expression reduction and MBNL-mediated alternative splicing defects were evident after degeneration (Wang et al., 2017), suggesting that MBNL1 reduction might contribute to synaptic transmission dysfunction in the DM1 brain.

### The Defective Neuroglial Interactions

A recent study shows that compared to neurons, cortical astrocytes contain more ribonuclear foci in DM1 transgenic mice (carrying 45 kb of human genomic DNA cloned from a DM1 patient) (Hernández-Hernández et al., 2013a). Meanwhile, RNA sequencing discovered more frequent splicing deficits in glia of DMSXL mice (Oude Ophuis et al., 2009; González-Barriga et al., 2021), suggesting a non-negligible presence of neuroglial damage in DM1. Glutamate transporter 1 (GLT1) is a glial-specific glutamate transporter that can recapture excitatory glutamate from the synaptic cleft and protect it from neurotoxicity caused by glutamate overstimulation (Bellamy, 2006). In the Bergmann glia of the cerebellum in DMSXL mice, scientists found glutamate excitotoxicity associated with decreased GLT1 (Sicot et al., 2017). In astrocytes of DMSXL mice, downregulation of GLT1 increased glutamate neurotoxicity and caused neuronal damage, while the upregulation of GLT1 corrected Purkinje cell firing and motor incoordination (Sicot et al., 2017). These studies indicated that the loss-of-function of GLT1 and defective neuroglial interactions might play critical roles in inducing DM1 brain disorders.

Nowadays, studies have identified that the expression of GLT1 is regulated by RNA transcription, splicing and stability, post-translational modifications, and protein activity (Kim et al., 2011). Specifically, loss-of-function of MBNL1 can perturb GLT1 polyadenylation, thus decreasing the levels of GLT1 glutamate transporter, while MBNL2 inactivation did not affect GLT1 levels, but contributed to the compensating increase in MBNL1 protein (Batra et al., 2014; Mohan et al., 2014). In the future, restoring GLT1 protein and glutamate neurotransmission by regulating MBNL proteins might be promising approaches to reverse the defective neuroglial interactions in DM1. Besides, in an inducible glial cell model of DM1 derived from human retinal Müller glial cells (MIO-M1) expressing 648 CUG repeats [MIO-M1 CTG(648)], scientists identified that the activation of inflammatory pathways and immune responses could partially explain DM1 CNS defects associated with defective glia (Azotla-Vilchis et al., 2021; González-Barriga et al., 2021). In glial cells of DMSXL mice, expanded CUG RNA affected preferentially differentiation-associated molecular events, which open new avenues in studying DM1 brain pathology with cell type resolution (González-Barriga et al., 2021).

### Altered Brain Insulin Signaling

Previous studies have observed the insulin resistance (IR) phenotype in DM1 patients (Moxley et al., 1978), which could be attributed to the mis-splicing of the insulin receptor gene. While recently, altered insulin signaling in the brain has also been proposed, thus provides potential alternative explanations for the DM1 neuropathogenesis (Nieuwenhuis et al., 2019). For example, animals with impaired insulin receptor signaling have shown reduced motivation, which could translate to apathy in humans (Dagenhardt et al., 2017), a critical symptom in adult DM1 patients (Gallais et al., 2015). In patients with obsessive-compulsive disorder, altered brain insulin signaling has been observed (van de Vondervoort et al., 2016). Besides, cognitive deficits, especially visuospatial and verbal memory deficits (Kullmann et al., 2016), depressive symptoms (Minier et al., 2018; van der Velden et al., 2019) and decreased behavioral flexibility (van de Vondervoort et al., 2019), have also been shown to be associated with brain IR. Moreover, a recent review pointed out the effects of insulin signaling on tauopathy and Aβ metabolism (Gonçalves et al., 2019). And several neuroimaging studies have discovered a decrease in glucose uptake in the brain of DM1 patients (Fiorelli et al., 1992; Peric et al., 2017a), however, it remains unclear whether altered insulin signaling is involved.

### Neurochemical Changes

A series of neurochemical changes were also found in DM1, which may account for specific symptoms. For example, the loss of serotonin (5-HT)-containing neurons in the dorsal raphe nucleus (DRN) and the superior central nucleus (SCN) of DM1 patients is associated with hypersomnia (Ono et al., 1998). And the alteration of serotonergic raphe structures might be involved in the pathogenesis of hypersomnia (Peric et al., 2014b; Krogias et al., 2015; Krogias and Walter, 2016). The hypoechogenicity of nucleus raphe might be correlated with EDS and depression in DM patients (Peric et al., 2014b). The extent of WM hyperintensities might be correlated with fatigue (Minnerop et al., 2011). Using conditional *Mbnl2*NEX-cKO mice (a tissue-specific knockout mouse model lacking the *Mbnl2* gene in forebrain glutamatergic neurons), long-term cognitive impairment and depression were found (Ramon-Duaso et al., 2020). This might be associated with significant neuronal loss, reduced neurogenesis, and fewer proliferating and immature neurons in the dentate gyrus of the HPC. Additionally, in *Mbnl2*–/– mice, increased proinflammatory microglia, dopamine levels as well as Dat, Drd1, and Drd2 transcripts levels, decreased expression of the brain derived-neurotrophic factor (BDNF) gene, and impaired neural spiking and oscillatory activities in the medial prefrontal cortex (mPFC) and the HPC have been found, accompanied with progressive cognitive and affective changes (Ramon-Duaso et al., 2019). Similar abnormalities have also been observed in the mPFC and HPC of DM1 patients with severe depression and cognitive impairment (Romeo et al., 2010b). Currently, chronic treatment with methylphenidate (MPH) has been shown to reverse the behavioral abnormalities, reduce proinflammatory microglia and Dat level, and increase BDNF and Nrf2 mRNA expressions in *Mbnl2*–/– mice. This makes it a promising drug candidate to treat CNS dysfunctions in DM1 patients (Ramon-Duaso et al., 2019). However, this intervention also impaired glutamate uptake and increased glutamate levels in juvenile rats (Schmitz et al., 2016, 2017), querying whether MPH therapy would increase glutamate neurotoxicity and induce the defective neuroglial interactions.

In addition, dysregulation of cerebrospinal fluid (CSF) homeostasis was observed in early onset DM1. A study introducing *DMPK* CTG expansions into the mouse found that mis-splicing significantly affected brain choroid plexus epithelial cells (Nutter et al., 2019). Besides, increased levels of total-Tau, IgG, γ-globulin, and myelin basic protein (MBP), as well as decreased levels of Aß1-42 and orexin-A were found in the CSF of DM1 patients (Hirase and Araki, 1984; Martínez-Rodríguez et al., 2003; Winblad et al., 2008; Peric et al., 2014a). Orexins are hypothalamic peptides that play critical roles in sleep/wake regulation (Sakurai, 2014). A possible correlation between an altered CSF orexin-A levels and EDS in systemic lupus erythematosus (SLE) patients with hypothalamic lesions and patients with frontotemporal dementia (FTD) have been reported (Çoban et al., 2013; Suzuki et al., 2018). Notably, significantly lower orexin-A levels were also detected in the CSF of 6 DM1 patients affected by EDS (Martínez-Rodríguez et al., 2003), thus providing potential explanations for DM1 sleep disorders. Furthermore, in 2015, a decreased level of BDNF, a neurotrophin participate in learning and memory, was detected in the serum of DM1 patients. Since it can cross the brain-blood barrier (BBB), it might be considered a promising biomarker of CNS defects (Comim et al., 2015).

### Neuropathological Defects in Early Developmental Processes

To determine the expression patterns of DMPK with age, Langbehn et al. (2021) analyzed the brain from 99 donors with DM1 ranging from 5 postconceptional weeks to 80 years old. They found that peak expression of wildtype *DMPK* coincides with a time of dynamic brain development, thus indicating that the abnormalities in DM1 brain *DMPK* expression may affect early brain development. Besides, direct injection of (CUG)_91_ repeat-containing mRNA into single-cell embryos of zebrafish induced CNS toxicity during early development, resulting in morphological abnormalities, behavioral abnormalities, and extensive transcriptional alterations, while co-injection of zebrafish *Mbnl2* RNA suppressed this toxicity and reversed the associated behavioral and transcriptional abnormalities (Todd et al., 2014). In addition, defects in the genes involved in dysfunctional neurite outgrowth and synaptogenesis at the neuromuscular junction were also observed in neuronal progeny derived from DM1 mutant human ES cells, which are associated with the decreased expression of two genes that belong to the *SLITRK* family, *SLITRK2* and *SLITRK4* (Marteyn et al., 2011). Transfection of *SLITRK2* and *SLITRK4* into cultured DM1 cells restored neurite length to control levels. However, it is interesting that *DMPK* mutation and dysregulation of splicing by MBNL1 do not appear to be involved in SLITRK misexpression or neurite outgrowth, suggesting other new molecular mechanisms involved in DM1 abnormal neurodevelopment (Marteyn et al., 2011).

## Pathological Features

### Tau Pathology

In the DM1 brain, dysregulation of alternative splicing could lead to pathologic Tau proteins accumulations and the formation of neurofibrillary tangles (NFTs) (Caillet-Boudin et al., 2014), which are mainly located in the HPC, entorhinal cortex, and most of the temporal areas, called Tau pathology. Nowadays, Tau pathology has been confirmed a critical histopathological characteristic in the brain of DM1 patients (Caillet-Boudin et al., 2014), and could interfere with axonal transport and neurosecretion (Caillet-Boudin et al., 2014). Although there is no direct evidence, current research suggests that this pathology may be related to cognitive dysfunctions in DM1.

In the adult brain, *Tau* gene could encode six *Tau* isoforms through alternative splicing of exons 2, 3, and 10, whereas in DM1, all of these exons are absent, thus promoting fetal expression of the 3-repeat *Tau* isoform (Sergeant et al., 2001; Jiang et al., 2004). Interestingly, a recent study in a congenital DM1 patient with intellectual disability also suggested the existence of a 4-repeat tau dominant pathology (Mizuno et al., 2018). Originally, cell studies identified that long CUG repeats-mediated loss-of-function of MBNL1 could be responsible for the changes in *Tau* splicing (Dhaenens et al., 2008). While in 2014, researchers discovered that both MBNL1 and MBNL2 have an enhancer activity of *Tau* exon 2 inclusion, and only the interaction of MBNL1 and MBNL2 can fully reverse the splicing defect of *Tau* exon 2 induced by the mutant CUG repeats, similar to that observed in DM1 (Carpentier et al., 2014). Then in 2015, a further study examining the *Tau* splicing using Nestin-Cre DKO mice (a *Mbnl1/2*–/– mouse model) suggested that both MBNL1 and MBNL2 synergize to regulate *Tau* exon 2, 3, and 10 splicing (Goodwin et al., 2015). These findings proved that the regulation of MBNL1/2 and their interactions are highly essential in DM1 Tau pathology. Except MBNL proteins, CELF proteins also serve as potential regulators of *Tau* splicing. Four exons (2, 3, 6, and 10) of *Tau* isoforms have been discovered to respond to one or more CELF proteins (Leroy et al., 2006a,b). Among them, *Tau* exon 10 responds specifically to CELF2 upregulation (Dhaenens et al., 2011). *Tau* exon 6 splicing is regulated by CELF5 and CELF6 (Leroy et al., 2006b). Notably, the heterogeneous distribution of Tau protein in the DM1 brain has also been reported, which may affect its splicing regulation patterns.

Other than Tau pathology, several kinds of protein and nucleotide deposits have also been observed in the brain of DM1 patients (Weijs et al., 2021), including Lewy bodies (LBs), neuronal intranuclear eosinophilic inclusion bodies, intracytoplasmic inclusion bodies, increased Marinesco bodies, gliosis (Itoh et al., 2010; Jinnai et al., 2013), skein-like ubiquitin-positive inclusions and granulovacuolar degeneration (GVD), which suggest neurodegeneration in the DM1 brain (Itoh et al., 2010; Yamazaki et al., 2011; Nakamori et al., 2012).

### RNAopathy and Spliceopathy

In the DM1 brain, the mutant *DMPK* RNA accumulates as nuclear foci in extensive areas, which contributes to abnormal alternative splicing (Miller et al., 2000; Fardaei et al., 2002). The nuclear foci are widely distributed throughout the brain of DM1 patients, including cortex, WM in subcortical and callosal areas, HPC, thalamus, brainstem, and cerebellum, and presented in various cell types, such as neurons, astrocytes, oligodendrocytes, Purkinje cells and human DM1 iPSC or ES-cell-derived NSCs (Jiang et al., 2004; Denis et al., 2013; Hernández-Hernández et al., 2013a). Their distributions varied from different genetic features, histological features and clinical features in each person (Jiang et al., 2004). Abnormal alternative splicing has been considered the critical pathogenesis of DM1. Changes in RNA-binding proteins, such as MBNL and CELF proteins, can lead to splicing defects of a variety of pre-mRNAs and misexpressions of different protein isoforms. Nowadays, the interaction between RNAopathy, spliceopathy, and Tau pathology have accounted for essential parts of DM1 neuropathology (Caillet-Boudin et al., 2014).

### Other Pathological Features

Other neuropathological features of DM1 include neuronal loss in different areas, such as the superficial layer of the frontal and parietal cortices, the occipital cortex, the medullary arcuate nuclei, the anterior and dorsomedial thalamic nuclei, the midbrain, and pontine reticular formation, which may be related to cortical atrophy (Culebras et al., 1973; Ono et al., 1995, 1998; Mizukami et al., 1999). The cell loss of specific areas might also contribute to the cognitive and behavioral abnormalities in DM1 patients (Rosman and Kakulas, 1966; Ono et al., 1996; Mizukami et al., 1999). In the post mortem brain and spinal cord of DM1 patients with congenital or childhood onset with intellectual deficiency, heterotopic neurons have been found, suggesting abnormal neurodevelopment (Ogata et al., 1998). Degenerative WM changes were also reported in DM1 patients, including myelin and axonal loss, expansion of perivascular spaces, gliosis, and hyalinization of capillaries in deep and subcortical WM (Itoh et al., 2010). However, most studies exploring the microscopic brain pathology in DM1 patients, as well as their relationships with neuroimaging features and splicing changes are case reports or small-scale researches. Clearly, larger follow-up studies are eagerly needed to improve the understandings of pathological alterations in DM1.

## Developmental or Neurodegenerative?

Multiple studies have identified a wide range of CNS alterations in DM1 patients. However, the varied characteristics between DM1 patients, such as CTG triplet expansion size, duration time, and age, significantly influence the cerebral performances. In several studies based on cross-sectional analyses, progressive GM loss and increased rate of cortex volume loss correlated with age have been reported (Weber et al., 2010; Minnerop et al., 2011; Caso et al., 2014; Serra et al., 2015; Zanigni et al., 2016; Cabada et al., 2017). A longitudinal study evaluating MRI in DM1 patients also revealed that the WM degeneration and ventricular enlargement progressed over time, though it varied between different individuals (Conforti et al., 2016). However, other studies did not find significant associations between WMHL and age or a significant increase of WML during disease progression (Meola and Sansone, 2007; Itoh et al., 2010; Bajrami et al., 2017; Cabada et al., 2017). Moreover, studies by Antonini et al. (2004) demonstrated a significantly reduced GM volume which was negatively correlated with age in DM1 patients, whereas WM volume was shown not to be correlated with cortical atrophy or age. Thus, further longitudinal evaluations are still need to assess spatiotemporal imaging changes. Regarding clinical features, Sansone et al. (2007) noted a progression in frontal cognitive impairment (attentional) in both DM1 and DM2 patients. Studies by Winblad and Gallais reported a cognitive decline in adult-onset DM1 is positively related to the earlier onset and longer duration of the disease (Winblad et al., 2016; Gallais et al., 2017). Moreover, a close relationship between CNS defects and CTG expansion size has also been discovered. For example, the impaired facial emotion recognition is significantly related to CTG repeat size (Winblad et al., 2006). The extent of GM and WM damage could be correlated with CTG expansion (Serra et al., 2015). And longer CTG expansion sizes could indicate a larger decrease in GM volume (Labayru et al., 2019). Further studies are needed to determine the progressive pattern of CNS dysfunctions.

Whether the progressive cerebral involvement in DM1 patients is due to a developmental or a neurodegenerative process is still an open question (Axford and Pearson, 2013). A variety of neurodegenerative pathological features, including NFTs, LBs and WM abnormalities, as well as a progressive cognitive decline reported by a limited number of longitudinal studies have been found in DM1 patients, which support that DM1 is in part a neurodegenerative process. However, another view supports that the progressive CNS dysfunction may be responsible for cognitive impairment, rather than neurodegenerative changes, since the distinct brain alteration patterns different from neurodegenerative features might also be associated with cognitive impairments in DM1 (Axford and Pearson, 2013; van der Plas et al., 2019; Labayru et al., 2020; Langbehn et al., 2021). A newly emerged opinion proposes that DM1 could be considered a progeroid disease (an early and accelerated aging process) (Sansone et al., 2007; Modoni et al., 2008; Caso et al., 2014; Winblad et al., 2016; Gallais et al., 2017; Solovyeva et al., 2021), as typical symptoms related to aging, such as cognitive decline, occur in the early years. However, a recent study shown an interesting contrast, that the presentation of simple tasks is hugely decreased while that of complex tasks are mostly retained in DM1, which is different from normal aging (Gallais et al., 2017). Further, it’s worth noting that in the congenital/childhood-onset DM1 patients, typical molecular and clinical deficits are observed during early developmental processes, which indicate an influence of DM1 on early brain development.

By the way, there are also some limited studies comparing the different characteristics between different phenotypes of DM1 (based on age-onset). In the congenital/childhood-onset DM1 patients, typical clinical deficits including intellectual disability and behavioral abnormalities are frequently reported, without further decline over these ages (Dhaenens et al., 2011; Caillet-Boudin et al., 2014; Fernandez-Gomez et al., 2019; Lindeblad et al., 2019). In addition, compared with adult-onset DM1 patients, verbal intelligence and memory was significantly deteriorated in juvenile-onset DM1 patients, reflecting a more pronounced developmental process in the juvenile type (Woo et al., 2019). However, in contrast, other studies demonstrated that adult-onset DM1 patients presented with more pronounced decrease of quality of life than juvenile-onset DM1 patients in almost all domains (Rakocevic-Stojanovic et al., 2014). A cross sectional study identified a significant cognitive impairment progression by aging in the majority of the cognitive domains in adult-onset DM1 patients (Baldanzi et al., 2016a), suggesting the existence of progressive degeneration. However, verbal memory abilities were relatively preserved, suggesting different changing patterns involved in memory and cognitive deficits (Baldanzi et al., 2016a). Regarding to neuroimaging observations, Caso et al. (2014) observed a more severe damage of GM in adult-onset DM1 patients than in juvenile-onset DM1 patients, supporting a degenerative origin of GM abnormalities. Conversely, the severe and distributed WM microstructural damage detected in both types might support a developmental change of microstructural WM damage. In 2020, the first longitudinal study of structural brain involvement in pediatric and adult/late-onset DM1 shown that the brain volume loss over time in both groups was not significant compared with their healthy controls, thus supporting the probable occurrence of the neurodevelopmental process. However, these findings cannot completely rule out the existence of the neurodegenerative process, since patients were not yet in their 60’s at follow-up (Labayru et al., 2020). In the future, additional studies with larger-sample and longitudinal observations are still needed to further clarify the feature of DM1 brain damages.

## Therapeutic Strategies of DM1

### Management of Neurological Defects

To date, there are no specific therapeutic agents available to reverse neurological defects in DM1. Modalities for management mainly rely on supportive care, including sleep hygiene improvement, cognitive−behavioral therapy, aerobic exercise training, and careful use of stimulant drugs. Sleep−related disorders have been recognized as primary symptoms of CNS involvement in DM1. Early recognition and treatment of sleep disorder breathing with nocturnal non-invasive mechanical ventilation have served as important countermeasures to deal with these problems (Pincherle et al., 2012). In a large clinical cohort study, scientists found that patients who insist on home mechanical ventilation for ≥5 h/24 h shown significantly higher survival rates than those who use it less (Seijger et al., 2021), and their tolerance and adherence were remarkably high. Besides, the biggest multicenter, randomized clinical trial in DM1 named the Observational Prolonged Trial in Myotonic Dystrophy Type 1 to Improve Quality of Life-Standards, a Target Identification Collaboration (OPTIMISTIC) revealed that cognitive behavioral therapy significantly improved the ability for activity and social participation at 10 months in severely fatigued patients with DM1 (NCT02118779) (Okkersen et al., 2018). Compared with usual care, cognitive behavioral therapy plus aerobic exercise training also increased the physical activity in DM1 patients (OPTIMISTIC) (van Engelen, 2015), indicating this therapy as a promising interventions for severe fatigue in DM1. Stimulant drugs also shown the potential to treat EDS. In 2007, the American Academy of Sleep Medicine (AASM) declared that MPH might be an effective tool for treating DM1-related EDS (Morgenthaler et al., 2007). However, a Cochrane review on well-designed psychostimulant trials in patients with DM1 and EDS pointed out the lack of evidence to support its routine use (Annane et al., 2002). Some clinical studies reported that modafinil might improve hypersomnia and fatigue without significantly increasing activity levels in DM1 (Hilton-Jones et al., 2012; Laberge et al., 2013). However, respiratory insufficiency (due to abnormal central drive and respiratory muscle weakness) and sleep fragmentation related to central or obstructive apnea must be excluded (Harper et al., 2002). Except for treating sleep-related disorders, in *Mbnl2*–/– mice, mirtazapine, a kind of antidepressant, has been discovered to reverse cognitive impairments and depression, as well as reduce microglia and neuronal loss (Ramon-Duaso et al., 2020). Moreover, metformin treatment reversed the metabolic and mitochondrial dysfunction and the accelerated aging process, such as impaired proliferation, in fibroblasts derived from DM1 patients (García-Puga et al., 2020). And its beneficial effects on muscle function have been confirmed in several clinical trials (Bassez et al., 2018). Since basic science reports have proposed the important role of abnormal insulin signaling in the brain (Nieuwenhuis et al., 2019), it indicates another possible therapeutic mechanism for metformin in DM1 brain. Furthermore, therapies *via* modulating glutamate levels and dopaminergic function have also emerged to attract more and more attention (Sicot et al., 2017; Serra et al., 2020b), which provide new insights for future DM1 CNS treatment.

Great progress of molecular therapies has been made in DM1, however, before we can move these treatments into clinical trials, there is also a great need to identify feasible outcome measures to evaluate the effectiveness of these therapies by characterizing the component of CNS deficits in DM1. Currently, the main outcome measures actually used to study CNS involvements in DM1 patients are represented by a variety of patient questionnaires and clinical neuropsychological tests which can assess cognitive and behavioral dysfunctions, and computerized neuroimaging techniques which can assess neuroimaging alterations (summarized in Tables 2–4). Besides, given the high heterogeneity of symptoms in DM1 patients, several patient-reported outcome measures are also developed, such as DM1 activity and participation scale for clinical use (DM1-ActivC), and the fatigue and daytime sleepiness scale (FDSS) (Hermans et al., 2013, 2015), which have shown valid measurements in DM1 populations (Angelini and Siciliano, 2020). Notably, due to the increasing risk and higher severity of COVID-19 in DM1 patients, and the difficulty in contacting a doctor during the COVID-19 epidemic, the Italian Association for Glycogenosis (AIG) developed telemedicine equipment, the AIGkit application (AIGkit app), which allows patients to receive constant remote monitoring by using dedicated questionnaires and getting personalized treatment (Symonds et al., 2017). A review by Simoncini et al. (2020) comprehensively summarized the clinical instruments available for cognitive and behavioral measures and neuroimaging assessment in DM1 brain. In the future, additional investigations are warranted to improve the reliability of these available measures, as well as to discover the new outcome markers.

### Potential Therapeutic Strategies

**FIGURE 2:**
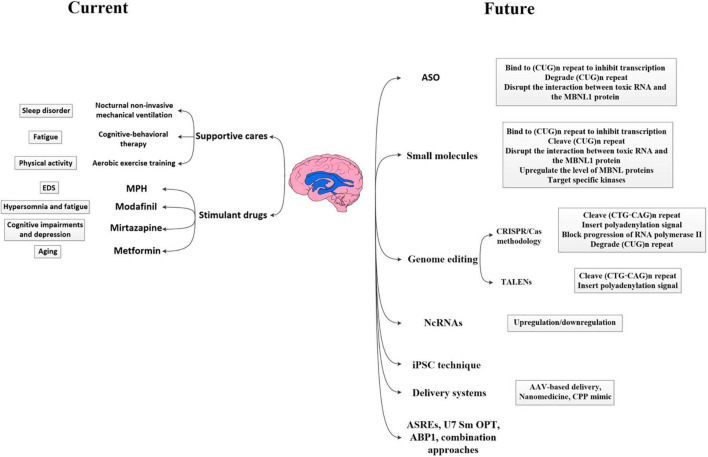
Current and future therapeutic strategies applied to DM1 CNS disorders. The therapeutic strategies for DM1 CNS disorders can be divided into available management measures and future promising strategies. Available modalities for management mainly rely on supportive care, including nocturnal non-invasive mechanical ventilation, cognitive–behavioral therapy, aerobic exercise training, and careful use of stimulant drugs such as MPH, modafinil, mirtazapine, and metformin. Future potential therapeutic strategies currently investigated include ASO-based therapy, small-molecule therapy, genome editing, ncRNAs-based therapy, iPSC technique and so on, which target different levels in the pathological process of DM1 (i.e., DNA level, RNA level, and protein level). Besides, the application of various effective delivery systems also improved the transportation efficiency and promoted the wide distribution of drugs. DM1, myotonic dystrophy type 1; MPH, methylphenidate; EDS, excessive daytime sleepiness; NcRNA, non-coding RNAs; iPSC, induced pluripotent stem cell; AAV, adeno-associated viral vector; ASREs, artificial site-specific RNA endonucleases; CPP, cell-penetrating peptide; ABP1, D-amino acid hexapeptide; ASO, antisense oligonucleotide; CRISPR-Cas9, clustered regularly interspaced short palindromic repeats (CRISPR)/CRISPR-associated system 9; TALEN, transcription activator-like effector nucleases; MBNL, muscleblind-like.

With significant advances in understanding the molecular pathogenesis of DM1, several approaches targeting disease mechanisms have been developed, such as antisense oligonucleotide (ASO)-based therapy, small-molecule therapy, genome editing, non-coding RNAs (ncRNAs)-based therapy, and iPSC technique, which target different steps in the pathological process of DM1 (Mulders et al., 2010; Magaña and Cisneros, 2011). Focusing on the most upstream target should block the initiation of the toxic cascade and correct more defects in tissues. Since most therapeutic efforts of DM1 mainly focused on the myocardium and skeletal muscle, and only few studies described their effects on the neurological defects, we briefly introduce their roles in the treatment of DM1 and their therapeutic potential in CNS deficits, hoping to provide references for DM1 CNS treatment. Figure 2 specifically introduced the current management measures as well as promising therapeutic strategies expected to be applied to DM1 CNS disorders.

#### Antisense Oligonucleotides

Antisense oligonucleotide is one of the important approaches which can target toxic RNA. It consists of a strand of nucleotides that can bind to a specific pre-mRNA/mRNA sequence and then alters protein synthesis through several mechanisms. In DM1, ASOs can interfere with the interaction between MBNL1 protein and toxic RNA, mainly by targeting the CUG repeat to reduce mutant transcripts, or by RNase-H-mediated degradation of expanded transcription (Klein et al., 2015). Other alterative mechanisms including inhibiting mRNA translation or altering RNA stability (Bennett and Swayze, 2010; Schoch and Miller, 2017).

Till now, various kinds of ASOs have shown efficacy *in vitro* and *in vivo* DM1 treatments. In 2003, scientists produced a retrovirus that expressed a 149-base pair (bp) antisense RNA, complementary to the (CUG)_13_ repeats and the proceeding 110-bp region (Furling et al., 2003). Injection of this ASO into human DM1 myoblasts significantly decreased toxic RNA and normalized CELF1 levels, eventually ameliorating the delay of muscle fusion and IR (Furling et al., 2003). However, this approach also reduced the level of normal *DMPK* transcripts and proteins. Since the unmodified ASOs are unstable and could easily be degraded, several chemical modifications to ASOs were developed to increase their stability and affinity for the target mRNA (Bennett et al., 2017; Khorkova and Wahlestedt, 2017). The first and the most widely used generation of modification was the phosphorothioate (PS) backbone modification (Bennett et al., 2017; Schoch and Miller, 2017), usually together with sugar modifications such as 2′-O-methyl (2′-O-Me) and 2′-O-methoxyethyl (2′-MOE). In 2009, a fully 2′-O-Me-PS-modified ASO, complementary to CUG repeats, called CAG7, was developed for DM1. Administration of this ASO in muscle tissue of DM500 mice (a DM1 model carrying > 300 *DMPK* CTG repeats) and HSA^LR^20b mice (a DM1 model expressing human skeletal actin transcripts containing ∼250 *DMPK* CTG repeats) silenced the expression of mutant RNA and decreased the formation of nuclear foci in a selective and (CUG)n-length-dependent manner (Mulders et al., 2009). Subsequently, morpholino ASO was discovered, which can bind to the toxic RNA and inhibit its interactions with proteins as well as disrupt CUG-exp-MBNL1 complexes. CAG25 was the first morpholino ASO to be used in DM1. Injection of CAG25 into muscle fibers of HSA^LR^ mice by intramuscular injection followed by *in vivo* electroporation significantly reversed myotonia within 4–5 weeks, accompanied with the increased translation of the mutant RNA (Wheeler et al., 2009). Since morpholino ASO does not induce cleavage of target mRNA, they do not affect normal *DMPK* transcripts and proteins (Summerton, 1999).

To date, it is still a question whether *DMPK* knout-out will indeed cause DM1 phenotype. Some studies reported that both *DMPK*± and *DMPK-*/*-* mice shown abnormal cardiac conduction (Berul et al., 1999, 2000), and homozygous deletion also exhibited skeletal myopathy and muscle weakness (Reddy et al., 1996), while other findings countered that the administration of *DMPK*-targeting ASOs with heterozygous deletion did not influence the normal cardiac or muscle function in mice, thereby supporting the feasibility and safety of ASOs usage in DM1 (Carrell et al., 2016). In the future, more relevant experiments are needed to reach a conclusion.

Since nuclear-retained CUG repeats are sensitive to antisense silencing, and RNase H are essentially ubiquitous expressed in nuclear (Suzuki et al., 2010), the recruitment of diverse gapmer-based ASOs shown promising futures *via* mediating RNase H cleavage and decay of the target RNA (Walder and Walder, 1988; Wheeler et al., 2012; Nguyen and Yokota, 2020). In HSA^LR^ mice, subcutaneous injection of gapmer ASOs significantly degraded expanded CUG transcripts as well as lncRNAs in skeletal muscle, reversing MBNL1 sequestration, myotonia, and mis-splicing without apparent off-target effects (Schoch and Miller, 2017). This strategy is also more attractive since it’s highly specific to expanded CUG repeats compared with normal-size repeats, and could maintain effects for 1 year after the treatment. Besides, BNANC gapmers targeting the DMPK 3′ UTR specifically knockdown the expanded CUG RNA and reversed the mis-splicing and RNA foci accumulation without inducing caspase activation (Manning et al., 2017). Moreover, the combinative application of gapmer and CAG25 morpholino produced synergistic effects to reduce expanded CUG repeats by 80% and almost eliminate RNA foci in DM1 cell culture, and get a smaller CUG repeats reduction in skeletal muscle of DM1 mice by half (Lee et al., 2012). However, the competition between gapmer and CAG25 in targeting the CUG repeats might limit their effects.

Other chemical modifications of ASOs also significantly optimized its characteristics. For example, the establishment of modified human U7 small nuclear RNAs (hU7-snRNAs), which contain a poly-CAG antisense sequence targeting the mutant CUG repeats, specifically degraded toxic RNA transcripts without influencing the products of wild-type DMPK alleles (Francois et al., 2011). Since the instability of expanded CTG repeats is a critical characteristic of DM1, which could be enhanced by RNA repeats, recent work shown that early intervention with CAG-repeat ASOs can not only reduce RNA toxicity but also stabilize CTG:CAG repeats at subpathogenic lengths in both DM1 human cells and transgenic mice model (Nakamori et al., 2011). One study using a 2′-4′-constrained ethyl-modified (cEt) ASO (ISIS 486178) effectively decreased *DMPK* mRNA levels in multiple organs of DMSXL mice or cynomolgus monkeys (Pandey et al., 2015). This ASO also exhibits a high level of RNA binding affinity and *in vivo* potency, without any association with muscle or cardiac toxicity. Furthermore, the systemic treatment with ASO (ISIS 486178) targeted to the non-CUG sequence within the 3′-UTR of *DMPK* also specifically rescued DM1 phenotypes of myotonia and cardiac conduction defects in DM200 mice [a DM1 model carrying a GFP-DMPK 3′-UTR (CTG) 200 transgene] (Jauvin et al., 2017; Yadava et al., 2020). In order to overcome the low efficiency of ASOs due to its wide distribution, Klein et al. (2019) developed an arginine-rich 466 cell-penetrating peptide (CPP). Compared with previous ASO strategies, this Pip6a-conjugated morpholino phosphorodiamidate oligomer (PMO) significantly increased ASO delivery into striated muscles after systemic administration in HSA^LR^ mice. Only low-dose treatment with Pip6a-PMO-CAG sufficiently rescued splicing defects and myotonia in mice (Klein et al., 2019). Hsieh et al. (2018) designed a short miniPEG-γ peptide with terminal pyrenees, which also exhibited high affinity and sequence specificity to toxic RNA repeats and successfully disrupted the CUG-exp–MBNL1 complex. With the new discovery of the imbalance in the splice isoform profile of *DMPK* in DM1 (Groenen et al., 2000; Wansink et al., 2003), two chemically modified ASOs were developed to prompt exon skipping from mRNA and degrade the CUG repeat from pre-mRNA in fibroblasts of DM1 patients. Disappointingly, neither strategy was as successful as predicted, but they did improve the DM1-related molecular phenotypes (Stepniak-Konieczna et al., 2020). It’s worth noting that a newly selected ASO called ISIS-DMPKRx (ISIS 598769) gapmer-type candidate has been evaluated in a Phase 1/2a clinical trial for the treatment of DM1 (Lim et al., 2017), for it can bind to a specific 3′-UTR gene sequence outside the CUG repeat and degrade toxic RNA (NCT023412011). Though it presented great safety and tolerance, IONIS proposed an inefficient effect on the functional and biological endpoints set in the trial since no sufficient drug could reach the muscles.

Though a variety of modifications have improved pharmacokinetic and pharmacodynamic properties of ASOs, the systemic and tissue-targeted implementation of ASOs are still challenging due to their poor intracellular uptake in some tissues (particularly skeletal muscle, heart and brain) (Muntoni and Wood, 2011). To overcome this disadvantages, different administration routes were developed to increase the tissue specificity. Nowadays, the delivery of ASOs to a single muscle by intramuscular injection have been frequently used in DM1 pre-clinical trials. Given the multi-organ or systemic features of DM1, systemic delivery *via* intraperitoneal, subcutaneous, or intravenous administration results in a rapid and widespread absorption of ASOs to various peripheral tissues (Yin et al., 2008; Hua et al., 2011). Since the highly charged ASOs cannot cross the BBB (Smith et al., 2006), intraventricular or intrathecal injections were invented to directly introduce ASOs into the CSF or parenchyma, which highly increased the delivery efficiency and ensured adequate distribution of drugs in the CNS (Miller et al., 2013; Chiriboga et al., 2016). However, though intrathecal delivery is currently shown safe and well-tolerated, it’s relatively invasive compared with other administrations. Identification of easier ASO delivery routes to the CNS such as intranasal administration may be an important step to promote their translation to human clinical trials. In addition, assisted delivery systems such as CPPs (Lebleu et al., 2008; Lehto et al., 2012; Boisguérin et al., 2015), nanoparticles (Wang et al., 2015) and adeno-associated virus (AAV) vectors (Danos, 2008), also increase the efficacy of ASOs, which would be introduced below.

#### Small Molecules

Recently, small-molecule-related strategies have received increasing attention in the treatment of DM1. Compared to other strategies, small molecules have many benefits, including low manufacturing cost, better oral delivery with shorter half-lives, longer shelf lives than other biologics, and sufficient biodistribution to affect multiple systems. Since a number of small molecules are existing drugs that explored for new applications, it also reduces the development time and potential risks of toxicity. However, despite the multiple advantages, most small molecules currently established can only target downstream processes to reduce toxic RNA or alter protein levels but cannot correct gene mutations. Meanwhile, their instability *in vivo* also limit their uses.

The mechanisms of molecular therapies can be divided into four aspects: inhibiting transcription of mutant RNA, cleaving CUG repeats, disrupting the interaction between toxic RNA and the MBNL1 protein, or targeting downstream pathways (Mulders et al., 2010; López-Morató et al., 2018; Reddy et al., 2019a). Cell and animal studies have indicated that pentamidine and a series of methylene linker analogs could exert beneficial effects by binding the CTG repeat DNA to inhibit transcription (Coonrod et al., 2013). One study using a DM1 HeLa cell model screened out that multiple microtubule inhibitors can target the toxic CUG RNA to reduce r(CUG)_480_ levels and then rescue mis-splicing to some extent, wherein the clinical microtubule inhibitor colchicine could even make positive effects in HSA^LR^ mice and primary DM1 patient-derived cells (Reddy et al., 2019b). This strategy provides a new avenue for DM1 research and suggests an alternative method of repeat-selective screening. A new molecule, JM642, was reported to have the capacity to bind to the expanded r(CUG) repeat and disrupt ribonuclear foci in the C2C12 DM1 cells and HSA^LR^ mice, finally rescuing mis-splicing (Nakatani et al., 2020). Moreover, several potential therapeutic molecules focus on cleaving the aberrant CUG repeats from disease-affected cells. For example, cugamycin is a small molecule that has been confirmed to selectively bind expanded CUG repeat conjugated to a bleomycin A5-cleaving module to cleave expanded CUG repeat. And deglycobleomycin, an analog in which the carbohydrate domain of bleomycin A5 is removed, significantly improves its selectivity by reducing DNA damage as well as maintaining the cleave ability (Angelbello et al., 2020). The major small-molecule compounds identified in DM1 therapy have been summarized in Table 5.

**TABLE 5:** Small molecules in DM1 treatments.

**References**	**Small molecules**	**Mechanisms**	**DM1 models**	**Effects**
**Inhibit transcription**				
Coonrod et al., 2013	Heptamidine	Interact with the CTG DNA	DM1 HSA^LR^ mice	Reduce CUG repeats Rescue mis-splicing
	Pentamidine and its analogs	Interact with the CTG DNA	DM1 HeLa cells	Rescue mis-splicing Rescue myotonia
Siboni et al., 2015b	Actinomycin D	Interact with the CTG DNA Block progression of the RNA polymerase	DM1 HeLa cells DM1 patient-derived fibroblasts DM1 HSA^LR^ mice	Reduce CUG repeats Reduce ribonuclear foci Rescue mis-splicing
Siboni et al., 2015a	Pentamidine and heptamidine	Inhibit transcription or reduce the stability of the transcript or bind to CUG RNA to displace MBNL proteins	DM1 HeLa cells DM1 HSA^LR^ mice	Reduce CUG RNA levels Reduce ribonuclear foci Rescue mis-splicing
Reddy et al., 2019b	Microtubule inhibitors	Transcriptional interference during failed repair of the expanded CTG repeat	DM1 HeLa cells	Reduce CUG repeats Rescue mis-splicing
	Colchicine	Transcriptional interference during failed repair of the expanded CTG repeat	DM1 HSA^LR^ mice DM1 patient-derived myotubes	Reduce CUG repeats Reduce ribonuclear foci Rescue mis-splicing
**Degrade the CUG repeat RNA**				
Angelbello et al., 2019	Cugamycin	Cleave the CUG repeat RNA	DM1 patient-derived myotubes DM1 HSA^LR^ mice	Rescue mis-splicing
Angelbello et al., 2020	A small-molecule-deglycobleomycin conjugate	Cleave the CUG repeat RNA	DM1 patient-derived myotubes DM1 C2C12 cells	Reduce CUG repeats with reduced DNA damage Reduce ribonuclear foci Rescue mis-splicing
**Disrupt the MBNL–CUG RNA interaction**				
Chakraborty et al., 2018	Daunorubicin	Bind to the CUG repeat RNA to displace MBNL1 protein	DM1 patient-derived myoblast cells DM1 patient-derived skin fibroblasts DM1 drosophila-derived cardiomyocytes	Rescue cardiac dysfunction Increase survival Reduce ribonuclear foci Rescue mis-splicing
Hoskins et al., 2014	Dilomofungin	Bind to the CUG repeat RNA to displace MBNL1 protein	C2C12 cells transfected with pLC16	Rescue mis-splicing Increase CUG repeats in nuclear foci
Nakamori et al., 2016	Erythromycin	Bind to the CUG repeat RNA to displace MBNL1 protein	DM1 C2C12 cells DM1 patient-derived fibroblasts DM1 HSA^LR^ mice	Reduce ribonuclear foci Rescue mis-splicing Rescue myotonia
Nakatani et al., 2020	JM642	Bind to the CUG repeat RNA to displace MBNL1 protein	DM1 C2C12 cells DM1 HSA^LR^ mice	Reduce ribonuclear foci Rescue mis-splicing
Warf et al., 2009	Pentamidine	Bind to the CUG repeat RNA to displace MBNL1 protein	DM1 HeLa cells DM1 HEK293 cells DM1 HSA^LR^ mice	Reduce ribonuclear foci Rescue mis-splicing Release MBNL1 from foci
	Neomycin B	Bind to the CUG repeat RNA to displace MBNL2 protein	DM1 HeLa cells	Disrupt the MBNL1–CUG RNA interaction without rescue mis-splicing of any of the tested targets
Childs-Disney et al., 2013	A thiophene-containing compound 1	Bind to MBNL1 protein to inhibits its interaction with RNA	DM1 HeLa cells DM1 C2C12 cells DM1 HEK 293T cells DM1 patient-derived fibroblasts	Improve DM1-associated translational defects Induce splicing defect
	A substituted naphthyridine compound 2	Bind to the CUG repeat RNA to displace MBNL1 protein	DM1 HeLa cells DM1 C2C12 cell DM1 HEK 293T cells	Improve DM1-associated translational defects Improve splicing defects
Luu et al., 2016	Two bisamidinium ligands-linked heterodimer	Bind to the CUG repeat RNA to displace MBNL1 protein	DM1 HeLa cells DM1 drosophila	Reduce ribonuclear foci Rescue mis-splicing Improve eye degeneration and larval crawling defect in drosophila
Ofori et al., 2012	Molecules with benzo[g]quinoline substructure	Bind to the CUG repeat RNA to displace MBNL1 protein	DM1 C2C12 cells DM1 HSA^LR^ mice	Release nuclear CUG-RNA retention Rescue mis-splicing
García-López et al., 2011	D-amino acid hexapeptide	Bind to the CUG repeat RNA to shift duplex it to a single-stranded form	DM1 HSA^LR^ mice DM1 drosophila	Reduce ribonuclear foci Rescue mis-splicing Reverse muscle histopathology
**Target downstream proteins**				
Wang et al., 2019	Tideglusib	Inhibit GSK3β activity	DM1 HSA^LR^ mice DM1 DMSXL mice CDM1 and DM1 patient-derived myoblasts	Normalize CELF1 activity Reduce CUG repeats Normalize CELF1- and MBNL1-regulated mRNA targets Improve postnatal survival and growth and neuromotor activity
Wojciechowska et al., 2014	C16 and C51	Inhibit ATP-binding site-specific kinase	DM1 patient-derived fibroblasts and myoblasts	Normalize CELF1 activity Reduce the size and number of ribonuclear foci Displace MBNL1 from foci Rescue mis-splicing
Zhang et al., 2017	ISOX and vorinostat	Upregulate MBNL1 protein levels	HeLa cell DM1 patient-derived fibroblasts	Increase MBNL1 protein levels Rescue mis-splicing
Wei et al., 2013	Lithium and TDZD-8	Inhibit GSK3β activity	DM1 HSA^LR^ mice	Normalize CELF1 and cyclin D3 activity Improve DM1 muscle function and histology
Chen et al., 2016	Phenylbutazone	Enhance MBNL1 transcription Attenuate binding of MBNL1 to expanded CUG RNA	DM1 C2C12 cells DM1 HSA^LR^ mice	Increase MBNL1 protein levels Rescue mis-splicing Disrupt MBNL1-CUG RNA interaction Improve DM1 wheel-running activity and muscle histopathology in mice
Wang et al., 2009	Ro-31-8220	Inhibit PKC activity	Tamoxifen-inducible heart-specific DM1 mice	Inhibit PKC-mediated elevation of CELF1 Increase survival Ameliorate the cardiac conduction defects and contraction abnormalities Rescue mis-splicing
Ketley et al., 2014	Ro 31-8220	Target unknown kinase	DM1 patient-derived myoblasts Embryos of zebrafish model	Eliminate nuclear foci Reduce MBNL1 protein in the nucleus Normalize CELF1 activity independent of PKC activity Rescue mis-splicing Rescue the mutant phenotype in zebrafish
Bargiela et al., 2019	Chloroquine	Upregulate MBNL1 and 2 protein levels	DM1 drosophila DM1 HSA^LR^ mice DM1 patient-derived myoblasts	Increase MBNL1 and 2 protein levels Rescue mis-splicing Restore locomotion in drosophila Restore muscle function and histopathology in mice
Yadava et al., 2015	P2D10	Anti-TWEAK activity	Transgenic DM1 mice	Block TWEAK/Fn14 signaling Improve muscle histopathology and functional outcomes
Brockhoff et al., 2017	AICAR	Activate AMPK activity	DM1 HSA^LR^ mice DM1 patient-derived myoblasts	Reduce ribonuclear foci Rescue mis-splicing Reduce myotonia in mice
	Rapamycin	Inhibit mTORC1 activity	DM1 HSA^LR^ mice DM1 patient-derived myoblasts	Improve muscle function *via* splicing-independent mechanisms
Oana et al., 2013	Manumycin A	Inhibit H-Ras farnesyltransferase activity	DM1 C2C12 cells DM1 HSA^LR^ mice	Rescue mis-splicing in mice
**Mixed mechanisms**				
Jenquin et al., 2018	Furamidine	Inhibit transcription Upregulate MBNL protein levels	DM1 HeLa cells DM1 patient-derived myotubes DM1 HSA^LR^ mice	Reduce CUG repeats Rescue mis-splicing Rescue gene expression Increase MBNL1 and MBNL2 proteins Disrupt the MBNL–CUG complex
Jenquin et al., 2019	A combination of erythromycin and furamidine	Inhibit transcription Disrupt the MBNL–CUG RNA interaction Upregulate MBNL protein levels	DM1 patient-derived myotubes DM1 HSA^LR^ mice	Reduce CUG repeats Rescue mis-splicing Rescue gene expression Increase MBNL1 and MBNL2 proteins Rescue myotonia in mice

*DM1, myotonic dystrophy type 1; CDM1, congenital DM1; MBNL, muscleblind-like; CELF, CUGBP/Elav-like family; GSK3β, Glycogen synthase kinase-3β; PKC, Protein kinase C; mTORC1, mTOR complex 1; Fn14, fibroblast growth factor-inducible 14.*

Some promising small molecules have even been tested in clinical trials. For example, AMO-02/tideglusib, a GSK-3β enzyme inhibitor, has been investigated for congenital DM by restoring the expression of CELF1 (Jones et al., 2012; Wang et al., 2019), which improved postnatal survival, weight, and neuromotor activity. Till now, a Phase II clinical trial of tideglusib on patients with congenital and juvenile-onset DM1 has already finished, and most participants presented with improved CNS and clinical neuromuscular performances (Horrigan et al., 2020). And a Phase II/III clinical trial on patients with congenital-onset DM1 is ongoing. In addition, MYD-0124 (erythromycin) and ERX-963 have been shown to bind to the CUG hairpin with high selectivity, reduce nuclear foci and reverse mis-splicing in DM1 vitro and vivo models. A Phase II clinical trial on adult patients with DM1 is currently underway to investigate the clinical effects of erythromycin after oral administration (Jenquin et al., 2019). Other molecules evaluated in clinical trials for specific disease symptoms (e.g., insulin resistance phenotype, myotonia, myalgia, or daytime sleepiness) include metformin, mexiletine, ranolazine, cannabinoids, pitolisant, Caffeine, and theobromine formulation MYODM^TM^ (Kouki et al., 2005; Logigian et al., 2010; Laustriat et al., 2015; Bassez et al., 2018; Vita et al., 2019; Heatwole et al., 2021; reviewed in Pascual-Gilabert et al., 2021).

#### Genome Editing

Compared with ASO therapy, the development of genome editing provides an opportunity for permanent corrections of gene mutation, which mainly includes clustered regularly interspaced short palindromic repeats (CRISPR)/CRISPR-associated system (Cas) methodology and transcription activator-like effector nucleases (TALENs) (Richard, 2015; Lee et al., 2016; Long et al., 2016; Nelson et al., 2017; Raaijmakers et al., 2019).

The CRISPR/Cas methodology can be used to target a specific genomic locus in the genome of eukaryotes (Knott and Doudna, 2018). Specifically, the Cas endonuclease is complexed with a small guide RNA (sgRNA) to target a specific genomic locus. Upon binding, the Cas protein generates a double-strand DNA (ds DNA) break by cleaving the DNA in both strands, thereby correcting the genetic defect (Raaijmakers et al., 2019). The main advantage of this strategy is to eliminate the disease defect at the DNA level, so the mutant transcripts and downstream dysregulations are not produced.

So far, the effects of CRISPR/Cas methodology have been validated. In 2017, one study reported that dual cleavage at either side of the CTG expansion can lead to complete and precise excision of the repeat tract from *DMPK* alleles in DM500 cells (myoblasts carrying 500 *DMPK* CTG repeats), myoblasts of DM1 patients, and unaffected individuals. And it also prevented damage to genes in the DM1 locus (van Agtmaal et al., 2017). Then, a CRISPR-Cas9 system from Staphylococcus aureus (Sa) was developed to cleave the CTG repeats in the human *DMPK* locus. A single intramuscular injection of recombinant AAV (rAAV) vectors expressing CRISPR-SaCas9 and selected sgRNAs has been shown to successfully delete the mutant CTG repeats in muscle fibers and reduce the RNA foci in myonuclei of DMSXL mice (Lo Scrudato et al., 2019).

Except for targeting the mutant DNA, RNA-targeting Cas9 (RCas9) systems which can bind to single-stranded RNA were also investigated (Strutt et al., 2018). Batra et al. (2017) found that through delivering a truncated RCas9 system in human DM1 cells, the toxic mRNAs were highly eliminated and aberrant splicing was corrected. This approach also has fewer side effects, since it does not affect normal transcripts. However, the delivery of RCas9 would gradually decay in the genome, which means the requirement of repeat treatments. Other scientists also proposed methods to prevent expanded transcription by inserting a homology-directed polyadenylation signal into the *DMPK* gene (Wang Y. et al., 2018), or recruiting catalytically deficient Cas9 (dCas9) to the repeat effectively to block progression of RNA polymerase II (Pinto et al., 2017). A recent review by Raaijmakers et al. (2019) carefully elaborated CRISPR/Cas-mediated approaches that target the causative mutation in the DNA and the RNA that cause DM1.

In recent years, some novel Cas-associated strategies have gradually emerged. For example, CRISPR-Cas13a is an RNA guided RNase. Zhang et al. (2020) using Leptotrichia shahii (Lsh) Cas13a in DM1 patient-derived myoblasts successfully degraded the expanded CUG RNA and reversed several important mis-splicing events. CRISPR/Cas9-associated base editing (BE) technology is a new therapy that do not rely on a dsDNA break at target sites but directly mediate the conversion of base pairs, and thus reduce the deletions or insertions (Komor et al., 2016; Gaudelli et al., 2017). However, the disability to generate precise edits beyond the allowed mutations might be a huge challenge.

Transcription activator-like effector nucleases is a newly discovered genome editing tool. It relies on modular transcription factors called transcription activator-like effectors, which enables the targeting of any specific DNA sequence (Sun and Zhao, 2013). Previous studies have reported that a dedicated TALEN can induce a dsDNA break into a CAG/CTG tri-nucleotide repeat in heterozygous yeast diploid cells, which shortened the repeat tract with nearly 100% efficacy and very high specificity (Richard et al., 2014). Currently, TALENs has shown great potential in the treatment of DM1. For example, TALENs application corrected the genetic defect of iPSCs, which contribute to the development of autologous stem cell therapy (Xia et al., 2015; Gao et al., 2016). By the way, TALENs seem to be the safest way to shorten trinucleotide repeats to non-pathological lengths, though more research is needed to combat possible off-target effects, immunogenicity to either the genome editing components or delivery particles and unpredictable DNA repair upon cleavage near the unstable repeat.

#### Non-coding RNAs

NcRNAs are RNA molecules that cannot be translated into proteins but are responsible for important regulatory events in cells (Fabbri et al., 2019). There are several types of ncRNAs, including microRNAs (miRNAs), long ncRNAs (lncRNAs), and circular RNAs (circRNAs). To date, global changes in ncRNA expression patterns in DM1 (Czubak et al., 2019a; López Castel et al., 2019; Voellenkle et al., 2019), as well as their key roles in contributing to DM pathogenesis have been presented (Perbellini et al., 2011; Wheeler et al., 2012; Gudde et al., 2016, 2017; Koutsoulidou et al., 2017; Czubak et al., 2019a,b; López Castel et al., 2019; Koehorst et al., 2020), which make them attractive biomarkers (Gambardella et al., 2010; Perbellini et al., 2011; Rau et al., 2011; Fernandez-Costa et al., 2013; Kalsotra et al., 2014; Perfetti et al., 2014, 2016; Koutsoulidou et al., 2015, 2017; Fritegotto et al., 2017; Koehorst et al., 2020; Pegoraro et al., 2020) and therapeutic targets in DM1 (Rau et al., 2011; Koutalianos et al., 2015; Zhang et al., 2016; Cerro-Herreros et al., 2018, 2020; López Castel et al., 2019; Sabater-Arcis et al., 2020). For example, muscle-specific miRNAs (myomiRNAs) like miR-1, miR-133a, miR-133b, and miR-206 as well as myostatin have been considered as attractive biomarkers of DM1 rehabilitation (Pegoraro et al., 2020). Besides, the therapeutic potential of miRNAs have been detected. Koutalianos et al. (2015) shown that in muscle cells from patients with congenital DM1, upregulation of the miR-206 expression by transfection with a miR-206 mimic into cells overexpressing CEFL1 induced myogenesis by inhibiting the expression of CELF1 and Twist-1. Replenishing of miR-7 with agomiR-7 reversed DM1 myoblast fusion defects and myotube growth, while blocking of miR-7 mediated by oligonucleotide worsen the outcomes (Sabater-Arcis et al., 2020). Silencing the regulatory dme-miR-277 and dme-miR-304 by miRNA sponge constructs successfully upregulated MBNL expression at the RNA and protein levels in a DM1 drosophila model, which then rescued mis-splicing and reduced muscle atrophy (Cerro-Herreros et al., 2016). This attempt evaluated miRNA sponge constructs as a powerful and attractive strategy to treat DM1 by blocking specific miRNAs. In recent years, RNA interference (RNAi) technology has attracted increased attention in DM1 (Bisset et al., 2015). Bisset et al. (2015) reported that miRNA-based RNAi hairpins delivered by rAAV vectors significantly downregulated mutant transcripts and reduced muscle pathology in HSA^LR^ mice. Besides, the intramuscular injection and electroporation of synthetic short interfering RNAs (siRNAs) significantly reduced toxic RNA transcripts and nuclear foci in HSA^LR^ mice (Sobczak et al., 2013). Antisense technology (antagomiRs) is a newly developed approach to block specific miRNA. Cerro-Herreros et al. (2020) reported that subcutaneous administration of antagomiR-23b in HSA^LR^ mice strongly increased the level of MBNL1 protein and reversed mis-splicing, grip strength, and myotonia in a dose-dependent manner. However, despite the huge potential of miRNA-based interventions, most of these attempts are still in preclinical phases because of their instability and delivery deficits.

#### Induced Pluripotent Stem Cell Technique

Nowadays, different differentiated cell models, such as neurons or muscle cells, have been used to investigate pathological mechanisms and to evaluate therapeutic strategies of DM1 before clinical validation (Larsen et al., 2011). However, these cell models are strongly limited due to the low *DMPK* transcript levels in affected cells and genetic background variation. Thus, there is a great need to generate alternative myogenic models that can be reliably used for *in vitro* disease modeling and/or drug screening purposes. It’s well-known that iPSCs are self-renewal and can differentiate into any cell type, including neurons and muscles cells. Since genome editing methodology could correct the genetic defect of iPSCs as mentioned before, the various phenotypes observed in DM1 can be subsequently reversed by correcting the lengths of CUG repeats in iPSCs and iPSC-derived cells, thereby offering a great translational platform for therapeutic development. Combining human iPSC lines and genome editing technology to create isogenic cell lines can also eliminate background genetic variation that might affect the expected results. In addition, reprogramming of somatic cells to iPSCs has been reported a valuable tool for disease modeling and drug discovery. Mondragon-Gonzalez and Perlingeiro (2018) established two DM1 iPSC lines from patient-derived fibroblasts, which then be differentiated into myotubes. The iPSC-derived myotubes highly recapitulate the molecular features of DM1 while ASO treatment successfully abolished RNA foci and rescued BIN1 mis-splicing. These results indeed confirmed DM1 iPSCs a kind of valuable alternative myogenic model to study DM1 pathogenesis and screen candidate drugs.

#### Assisted Delivery Systems

The inefficacy of conventional drug delivery and low bioavailability of drugs led to the rapid progress of assisted delivery systems in recent years. Currently, a number of delivery strategies have shown great potential in enhancing the efficacy of therapeutic molecules while minimizing their off-target effects, which can be broadly divided into four types: polymeric, peptide, lipid, and viral delivery systems, wherein AAV-based gene therapy has emerged to be a potent and promising therapeutic tool for DM1. Compared with other strategies, AAV vectors permanently change genetic defects and avoid repeated administrations. Given the multisystemic symptoms of DM1, they can also achieve systemic delivery (Gregorevic et al., 2004; Arruda et al., 2005). Kanadia et al. (2006) reported that upregulation of MBNL1 by AAV-mediated transfection to skeletal muscle effectively reversed mis-splicing and myotonia in HSA^LR^ mice. Besides, systemic AAV-delivered RNAi significantly improved disease phenotype in HSA^LR^ mice (Bisset et al., 2015). Currently, a possible AAV-delivered ASO, AT466 is being established to reduce the toxic RNA levels in cells derived from DM1 patients by RNA degradation, exon skipping or both (Audentes).^1^ Recent efforts on viral delivery to the CNS have also taken an exciting leap forward (Miller et al., 2012). The delivery of AAV9 *via* intravenous administration could traverse the BBB in both neonate and adult animals (van der Bent et al., 2018), which provides a promising tool to treat CNS disorders.

The hydrophilic nature as well as large size and high charge of peptides and proteins prevents their penetration across biological membranes. In order to achieve delivery of therapeutic peptide and protein into cells as well as across epithelial barriers and the BBB, a family of delivery vectors called CPPs has been developed, which shown the potential to traverse cellular membranes and promote the uptake of therapeutic peptides with lower toxicity. To date, CPPs have been applied for intracellular, transepithelial, and transendothelial delivery of various therapeutic cargos. Regarding DM1, one study using the CPP mimic as a scaffold to assemble the multivalent ligand construct significantly increased their binding affinity, which then contributed to phenotypic improvement in a DM1 drosophila model (Bai et al., 2016). Compared to unconjugated PMO, systemic administration of Pip6a-conjugated morpholino PMO remarkably enhanced ASO delivery into DM1 mice muscles. Besides, several CPPs have also been identified to mediate cargo delivery across the BBB and exerted protection in the brain (Kilic et al., 2003, 2004; Dietz et al., 2006). And the combination of CPPs and intranasal administration may further enhance CNS delivery (Kamei and Takeda-Morishita, 2015; McGowan et al., 2016; Khafagy et al., 2020; Akita et al., 2021). However, the BBB-specific CPPs remain not to be found and the mechanisms driving the transportation of CPPs are still unknown.

In order to overcome the instability, limited distribution, rapid degradation and toxicity of therapeutic molecules, nanomedicine has been rapidly developed as an effective drug delivery system in recent years, which greatly improved the efficiency and tissue compatibility of gene therapy, increased safety, and ensured systemic distribution (Koebis et al., 2013; Hermans et al., 2015; Lee et al., 2017; Amini et al., 2019), relying on their adjustable physicochemical properties to prevent toxicity and to carry specific biological molecules to target sites (Andreana et al., 2021). In HSA^LR^ mice, using bubble liposomes as delivery tools significantly improved the delivery efficiency of PMO into muscles, which then increased the expression of chloride channel 1 (Clcn1) protein in skeletal muscle and ameliorated the myotonia (Koebis et al., 2013). Besides, given the inefficiency of macromolecules to cross the BBB, the small nanocarriers-mediated transport, including active transport (e.g., receptor-mediated endocytosis) and facilitated diffusion, also provided a promising pathway for smooth BBB passage of large cargos and targeting various cells with intracellular localization specificity (Hernando et al., 2018; Di Filippo et al., 2021; Nehra et al., 2021; Song et al., 2021). Toward future directions, the combinative applications of these delivery vectors and administration routes would attracted more and more attentions since they provide numerous chances for the discovery of both safe and effective delivery strategies to avoid the side effects of any signal technique.

#### Other Potential Strategies

Artificial site-specific RNA endonucleases (ASREs) are newly discovered molecules specifically targeting mutant RNA accumulated in the nucleus. The results of Zhang et al. (2014) shown that ASRE treatment significantly decreased nuclear foci formation and reversed the mis-splicing of DM1-related genes with few side effects on wild-type alleles. U7 small nuclear ribonucleoproteins (snRNPs) are a specific type of snRNPs that do not participate in splicing mediation but is a key factor in the unique 3′ end processing of replication-dependent histone (RDH) pre-mRNAs. The modified U7 snRNP (U7 Sm OPT) has been used as a promising tool for gene therapy in splicing defects-associated diseases by targeting splicing to induce efficient skipping or inclusion of selected exons. It has multiple advantages, such as small size, good stability, ability to accumulate in the nucleus without toxicity and immunoreactivity, and low risk of transgene dysregulation. In addition, using U7 Sm OPT as a tool in gene therapy also ensures lifelong treatment. Gadgil and Raczyńska et al. (2021) demonstrated that incorporating ASO into the U7 Sm OPT successfully avoided repeated administration. Injection of U7 Sm OPT containing ASO with 15 CAG repeats in skeletal muscle cells isolated from DM1 patients resulted in long-time improvement in splicing and differentiation defects in a dose-dependent manner, without affecting the wild-type *DMPK* transcripts (Le Hir et al., 2013). The critical properties of U7 snRNP might deploy it as a new tool in gene therapy in the future. In a DM1 drosophila model, researchers screened a D-amino acid hexapeptide (ABP1) which can induce the CUG hairpin into a single-stranded conformation and bind the CUG RNA without displacing MBNL1. Compared to ASOs, this method avoids affecting endogenous transcripts. In fly eyes and muscles, overexpression of natural, L-amino acid ABP1 analogs reduced RNA toxicity. And in HSA^LR^ mice, ABP1 reversed muscle histopathology and partially rescued mis-splicing of MBNL1 targets (García-López et al., 2011). Notably, combination approaches such as different small molecules or gene therapies targeting different processes have also attracted increasing attention. This may produce greater benefits in disease modulation than simply additional effects. Importantly, this combination treatment might also reduce off-target effects.

### Therapeutic Strategies Targeting Central Nervous System

The current advances in therapeutic strategies should also be applicable in principle to CNS since the pathogenesis of CNS deficits is similar to that of other organs, such as RNA toxicity and splicing defects. However, the existence of BBB hinders the delivery and distribution of drugs in the brain. Therefore, the ideal therapeutic agents need to be able to cross the BBB readily. Some improvements have been made, such as intracellular delivery of the therapeutic molecules to promote uptake, administering molecules intracerebroventricularly or intrathecally (Baughan et al., 2009; Geary et al., 2015), or regulating the molecule size and charge to achieve an efficient delivery to the brain. Recent advances also found that intranasal delivery efficiently bypasses the BBB and highly increases the CNS concentrations of drugs and is non-invasive. Besides, the combination of U7 methodology with highly efficient AAV-mediated delivery, receptor-mediated endocytosis of ASOs, and nanoparticles-, exosomes- or CPP-based delivery of large cargos also favor the BBB passage and the higher distributions of drugs in the CNS (Foust and Kaspar, 2009; Krupa et al., 2014; McGowan et al., 2015, 2016; Bai et al., 2016; Kristensen et al., 2016). In conclusion, all of these therapeutic agents, administration routes, and assisted delivery systems create numerous chances for the discovery of effective therapies for CNS disorders.

Several molecules applied for CNS treatment have shown positive effects in preclinical trials, which may provide new thoughts for DM1 CNS treatment. For example, in mice overexpressing *APP*, intracerebroventricular injections of PS-modified ASOs significantly reduced the expression of APP protein and improved learning and memory deficits (Kumar et al., 2000). In the Alzheimer’s disease (AD) mouse model, a designed 2′-O-Me-PS-modified ASO sustainly increased exon 19 splicing of apolipoprotein E receptor 2 (ApoER2) as well as restored synaptic function and learning and memory (Hinrich et al., 2016). Besides, an 2′MOE-modified ASO called IONIS MAPTRx (ISIS 814907) has been evaluated in a phase I/II study in patients with mild AD (NCT03186989), as it highly reduced the expression of tau protein through targeting MAPT mRNA. Hu et al. (2021) using a 20-mer RNase H-active gapmer ASO combined with a 3-month exercise training program in old HSA^LR^ mice reversed all measures of fatigue, though they did not detect the index of fatigue due to CNS dysfunctions. These findings may provide new thoughts for DM1 CNS treatment. Murlidharan et al. (2016) discovered a lab-derived AAV chimeric (AAV2g9), which has favorable CNS properties derived from both parental counterparts, AAV2 and AAV9. Administration of CRISPR/Cas9 with this synthetic AAV vector into the CSF minimized systemic leakage and reduced the sequestration and gene transfer in off-target organs.

The modification of stem cells also offers a new chance for CNS treatment. Previous studies have shown that NSC derived from ES cells or iPSCs could present critical features of DM1 (Marteyn et al., 2011; Denis et al., 2013; Xia and Ashizawa, 2015; Xia et al., 2015). In DM1 NSCs, insertion of poly A signals upstream of *DMPK* CTG repeats by TALEN-mediated homologous recombination significantly eliminated mutant transcripts and nuclear RNA foci, corrected mis-splicing, and ultimately reversed phenotypes (Xia et al., 2015; Gao et al., 2016). Despite the broad prospects of strategies in CNS treatment, there are still many challenges, such as lower efficiency and distribution to CNS and safety regarding immune response and gene therapy specificity. In addition, no studies have discussed the targeted brain cell types of different strategies and the corresponding alterations of brain cells after treatment. The resolution of these issues may provide a deeper understanding of the therapeutic mechanisms of drugs.

## Limitations and Future Directions

Though great progresses have been made, there are still many limitations in current studies that hinder the understanding of the complex nature of CNS defects and the development of new treatments. Firstly, despite the current knowledge of the molecular basis underlying CNS defects, it is still unclear how these molecular alterations could be translated into specific pathological changes and clinical symptoms, nor do we know how these deficits progress over time. In the future, more longitudinal studies with large sample size are needed to fully understand the DM1 neuropathology and its progression. Secondly, although several cell and animal models have been developed to reproduce the DM1 gene mutation and pathogenesis similar to the human phenotype, such as MBNL-loss or CELF-overexpressing phenotypes, or *in vitro* and *ex vivo* alternatives which can assist animal experimentation, none of them completely recreate the multisystemic phenotypes of DM1, which block the in-depth assessment of DM1 defects and effective evaluations of feasible interventions. Thirdly, more applicable patient questionnaires and clinical neurocognitive evaluation protocols should be investigated to better identify the disease component and serve as potential markers to evaluate the effectiveness of therapeutic strategies. Fourthly, at the pharmacotherapeutic level, more efforts are needed to improve drug delivery efficiency, biodistribution and availability, and reduce toxicity. Meanwhile, the design of drugs for CNS treatment must be able to readily cross the BBB.

## Conclusion

In this review, we primarily focus on CNS involvement in DM1, outlining the primary pathological alterations and pathogenesis underlying CNS. Then, we highlight promising therapeutic strategies for DM1. Excepting for some available drugs targeting for specific neurological impairments, the promising results of some biological molecules (i.e., ASO, small molecules, CRISPR/Cas9, ncRNA.) *via* targeting mutant DNA, RNA, or downstream proteins in preclinical models or even clinical trials have also provided relevant candidates for DM1 treatment. Particularly, a variety of re-purposed small molecules drugs have been evaluated in DM1, which present with low manufacturing cost, greater safety and stability *in vivo* treatment. Meanwhile, the development of screening and rational design technologies also promote the production of newly potent small molecules, which increases the availability of small molecule therapy in DM1 in the near future. In principle, these interventions should be applicable to the CNS, since strategies such as eliminating toxic RNAs, reducing the formation of nuclear foci, or restoring the levels of splicing-related factors are also effective in brain cells, however, additional researches are still needed to improve the understanding of DM1 progression and to transfer available therapeutic strategies and knowledge into actionable clinical applications.

## Author Contributions

JL wrote the first draft of the manuscript. X-LY and Z-NG prepared the figures. SH and YY reviewed and edited the manuscript. All authors contributed to critical revision of the manuscript and approved the final manuscript for submission.

## Conflict of Interest

The authors declare that the research was conducted in the absence of any commercial or financial relationships that could be construed as a potential conflict of interest.

## Publisher’s Note

All claims expressed in this article are solely those of the authors and do not necessarily represent those of their affiliated organizations, or those of the publisher, the editors and the reviewers. Any product that may be evaluated in this article, or claim that may be made by its manufacturer, is not guaranteed or endorsed by the publisher.

## Footnotes

1
www.audentestx.com/innovative-therapies

## References

[B1] Akiguchi I. Nakano S. Shiino A. Kimura R. Inubushi T. Handa J. (1999). Brain proton magnetic resonance spectroscopy and brain atrophy in myotonic dystrophy. *Arch. Neurol.* 56 325–330. 10.1001/archneur.56.3.325 10190823

[B2] Akita T. Kimura R. Akaguma S. Nagai M. Nakao Y. Tsugane M. (2021). Usefulness of cell-penetrating peptides and penetration accelerating sequence for nose-to-brain delivery of glucagon-like peptide-2. *J. Control. Release* 335 575–583. 10.1016/j.jconrel.2021.06.007 34116136

[B3] Amini M. A. Abbasi A. Z. Cai P. Lip H. Gordijo C. R. Li J. (2019). Combining tumor microenvironment modulating nanoparticles with doxorubicin to enhance chemotherapeutic efficacy and boost antitumor immunity. *J. Natl. Cancer Institute* 111 399–408. 10.1093/jnci/djy131 30239773

[B4] Anderson P. J. (2013). Leader of the pack - neuropsychological assessment, 5th Edition, Muriel Lezak, Diane B. Howieson, Erin D. Bigler, & Daniel Tranel. *J. Int. Neuropsychol. Soc.* 19 488–489. 10.1017/S1355617713000337

[B5] Andreadis A. (2012). Tau splicing and the intricacies of dementia. *J. Cell. Physiol.* 227 1220–1225. 10.1002/jcp.22842 21604267 PMC3177961

[B6] Andreana I. Repellin M. Carton F. Kryza D. Briançon S. Chazaud B. (2021). Nanomedicine for gene delivery and drug repurposing in the treatment of muscular dystrophies. *Pharmaceutics* 13:278. 10.3390/pharmaceutics13020278 33669654 PMC7922331

[B7] Angeard N. Gargiulo M. Jacquette A. Radvanyi H. Eymard B. Héron D. (2007). Cognitive profile in childhood myotonic dystrophy type 1: is there a global impairment? *Neuromusc. Disord.* 17 451–458. 10.1016/j.nmd.2007.02.012 17433680

[B8] Angeard N. Jacquette A. Gargiulo M. Radvanyi H. Moutier S. Eymard B. (2011). A new window on neurocognitive dysfunction in the childhood form of myotonic dystrophy type 1 (DM1). *Neuromusc. Disord.* 21 468–476. 10.1016/j.nmd.2011.04.009 21592796

[B9] Angelbello A. J. DeFeo M. E. Glinkerman C. M. Boger D. L. Disney M. D. (2020). Precise targeted cleavage of a r(CUG) repeat expansion in cells by using a small-molecule-deglycobleomycin conjugate. *ACS Chem. Biol.* 15 849–855. 10.1021/acschembio.0c00036 32186845 PMC7360342

[B10] Angelbello A. J. Rzuczek S. G. McKee K. K. Chen J. L. Olafson H. Cameron M. D. (2019). Precise small-molecule cleavage of an r(CUG) repeat expansion in a myotonic dystrophy mouse model. *Proc. Natl. Acad. Sci. U.S.A.* 116 7799–7804. 10.1073/pnas.1901484116 30926669 PMC6475439

[B11] Angelini C. Pinzan E. (2019). Advances in imaging of brain abnormalities in neuromuscular disease. *Ther. Adv. Neurol. Disord.* 12:1756286419845567. 10.1177/1756286419845567 31105770 PMC6503605

[B12] Angelini C. Siciliano G. (2020). Neuromuscular diseases and Covid-19: advices from scientific societies and early observations in Italy. *Eur. J. Transl. Myol.* 30:9032. 10.4081/ejtm.2019.9032 32782765 PMC7385692

[B13] Angelini C. Tasca E. (2012). Fatigue in muscular dystrophies. *Neuromusc. Disord.* 22(Suppl. 3), S214–S220. 10.1016/j.nmd.2012.10.010 23182642 PMC3526799

[B14] Annane D. Miller R. Barnes P. (2002). Psychostimulants for hypersomnia (excessive daytime sleepiness) in myotonic dystrophy. *Cochrane Database Syst. Rev.* 4:Cd003218. 10.1002/14651858.cd003218 12519589

[B15] Antonini G. Mainero C. Romano A. Giubilei F. Ceschin V. Gragnani F. (2004). Cerebral atrophy in myotonic dystrophy: a voxel based morphometric study. *J. Neurol. Neurosurg. Psychiatry* 75 1611–1613. 10.1136/jnnp.2003.032417 15489397 PMC1738796

[B16] Arruda V. R. Stedman H. H. Nichols T. C. Haskins M. E. Nicholson M. Herzog R. W. (2005). Regional intravascular delivery of AAV-2-F.IX to skeletal muscle achieves long-term correction of hemophilia B in a large animal model. *Blood* 105 3458–3464. 10.1182/blood-2004-07-2908 15479726 PMC1895010

[B17] Axford M. M. Pearson C. E. (2013). Illuminating CNS and cognitive issues in myotonic dystrophy: workshop report. *Neuromusc. Disord.* 23 370–374. 10.1016/j.nmd.2013.01.003 23453858

[B18] Azotla-Vilchis C. N. Sanchez-Celis D. Agonizantes-Juárez L. E. Suárez-Sánchez R. Hernández-Hernández J. M. Peña J. (2021). Transcriptome analysis reveals altered inflammatory pathway in an inducible glial cell model of myotonic dystrophy type 1. *Biomolecules* 11:159. 10.3390/biom11020159 33530452 PMC7910866

[B19] Bai Y. Nguyen L. Song Z. Peng S. Lee J. Zheng N. (2016). Integrating display and delivery functionality with a cell penetrating peptide mimic as a scaffold for intracellular multivalent multitargeting. *J. Am. Chem. Soc.* 138 9498–9507. 10.1021/jacs.6b03697 27355522

[B20] Bajrami A. Azman F. Yayla V. Cagirici S. Keskinkiliç C. Sozer N. (2017). MRI findings and cognitive functions in a small cohort of myotonic dystrophy type 1: retrospective analyses. *Neuroradiol. J.* 30 23–27. 10.1177/1971400916678223 27837184 PMC5564333

[B21] Baldanzi S. Cecchi P. Fabbri S. Pesaresi I. Simoncini C. Angelini C. (2016b). Relationship between neuropsychological impairment and grey and white matter changes in adult-onset myotonic dystrophy type 1. *Neuroimage Clin.* 12 190–197. 10.1016/j.nicl.2016.06.011 27437180 PMC4939389

[B22] Baldanzi S. Bevilacqua F. Lorio R. Volpi L. Simoncini C. Petrucci A. (2016a). Disease awareness in myotonic dystrophy type 1: an observational cross-sectional study. *Orphanet J. Rare Dis.* 11:34. 10.1186/s13023-016-0417-z 27044540 PMC4820880

[B23] Bargiela A. Sabater-Arcis M. Espinosa-Espinosa J. Zulaica M. Lopez de Munain A. Artero R. (2019). Increased Muscleblind levels by chloroquine treatment improve myotonic dystrophy type 1 phenotypes in in vitro and in vivo models. *Proc. Natl. Acad. Sci. U.S.A.* 116 25203–25213. 10.1073/pnas.1820297116 31754023 PMC6911202

[B24] Bassez G. Audureau E. Hogrel J. Y. Arrouasse R. Baghdoyan S. Bhugaloo H. (2018). Improved mobility with metformin in patients with myotonic dystrophy type 1: a randomized controlled trial. *Brain* 141 2855–2865. 10.1093/brain/awy231 30169600

[B25] Batra R. Charizanis K. Manchanda M. Mohan A. Li M. Finn D. J. (2014). Loss of MBNL leads to disruption of developmentally regulated alternative polyadenylation in RNA-mediated disease. *Mol. Cell* 56 311–322. 10.1016/j.molcel.2014.08.027 25263597 PMC4224598

[B26] Batra R. Nelles D. A. Pirie E. Blue S. M. Marina R. J. Wang H. (2017). Elimination of toxic microsatellite repeat expansion RNA by RNA-targeting Cas9. *Cell* 170 899.e10–912.e10. 10.1016/j.cell.2017.07.010 28803727 PMC5873302

[B27] Baughan T. D. Dickson A. Osman E. Y. Lorson C. L. (2009). Delivery of bifunctional RNAs that target an intronic repressor and increase SMN levels in an animal model of spinal muscular atrophy. *Hum. Mol. Genet.* 18 1600–1611. 10.1093/hmg/ddp076 19228773 PMC2667287

[B28] Bellamy T. C. (2006). Interactions between *Purkinje neurones* and *Bergmann glia*. *Cerebellum* 5 116–126. 10.1080/14734220600724569 16818386

[B29] Bennett C. F. Baker B. F. Pham N. Swayze E. Geary R. S. (2017). Pharmacology of antisense drugs. *Annu. Rev. Pharmacol. Toxicol.* 57 81–105. 10.1146/annurev-pharmtox-010716-104846 27732800

[B30] Bennett C. F. Swayze E. E. (2010). RNA targeting therapeutics: molecular mechanisms of antisense oligonucleotides as a therapeutic platform. *Annu. Rev. Pharmacol. Toxicol.* 50 259–293. 10.1146/annurev.pharmtox.010909.105654 20055705

[B31] Bertrand J. A. Jean S. Laberge L. Gagnon C. Mathieu J. Gagnon J. F. (2015). Psychological characteristics of patients with myotonic dystrophy type 1. *Acta Neurol. Scand.* 132 49–58. 10.1111/ane.12356 25496310

[B32] Berul C. I. Maguire C. T. Aronovitz M. J. Greenwood J. Miller C. Gehrmann J. (1999). DMPK dosage alterations result in atrioventricular conduction abnormalities in a mouse myotonic dystrophy model. *J. Clin. Investig.* 103 R1–R7. 10.1172/jci5346 10021468 PMC408103

[B33] Berul C. I. Maguire C. T. Gehrmann J. Reddy S. (2000). Progressive atrioventricular conduction block in a mouse myotonic dystrophy model. *J. Intervent. Cardiac Electrophysiol.* 4 351–358. 10.1023/a:1009842114968 10936001

[B34] Bird T. D. (1993). “Myotonic dystrophy type 1,” in *Genereviews((r))*, eds Adam M. P. Ardinger H. H. Pagon R. A. Wallace S. E. Bean L. J. H. Stephens K. (Seattle, WA: University of Washington).

[B35] Bisset D. R. Stepniak-Konieczna E. A. Zavaljevski M. Wei J. Carter G. T. Weiss M. D. (2015). Therapeutic impact of systemic AAV-mediated RNA interference in a mouse model of myotonic dystrophy. *Hum. Mol. Genet.* 24 4971–4983. 10.1093/hmg/ddv219 26082468 PMC4527493

[B36] Boisguérin P. Deshayes S. Gait M. J. O’Donovan L. Godfrey C. Betts C. A. (2015). Delivery of therapeutic oligonucleotides with cell penetrating peptides. *Adv. Drug Deliv. Rev.* 87 52–67. 10.1016/j.addr.2015.02.008 25747758 PMC7102600

[B37] Breton É Légaré C. Overend G. Guay S. P. Monckton D. Mathieu J. (2020). DNA methylation at the DMPK gene locus is associated with cognitive functions in myotonic dystrophy type 1. *Epigenomics* 12 2051–2064. 10.2217/epi-2020-0328 33301350

[B38] Brockhoff M. Rion N. Chojnowska K. Wiktorowicz T. Eickhorst C. Erne B. (2017). Targeting deregulated AMPK/mTORC1 pathways improves muscle function in myotonic dystrophy type I. *J. Clin. Investig.* 127 549–563. 10.1172/jci89616 28067669 PMC5272183

[B39] Brook J. D. McCurrach M. E. Harley H. G. Buckler A. J. Church D. Aburatani H. (1992). Molecular basis of myotonic dystrophy: expansion of a trinucleotide (CTG) repeat at the 3′ end of a transcript encoding a protein kinase family member. *Cell* 68 799–808. 10.1016/0092-8674(92)90154-5 1310900

[B40] Cabada T. Díaz J. Iridoy M. López P. Jericó I. Lecumberri P. (2020). Longitudinal study in patients with myotonic dystrophy type 1: correlation of brain MRI abnormalities with cognitive performances. *Neuroradiology* 63 1019–1029. 10.1007/s00234-020-02611-9 33237431

[B41] Cabada T. Díaz J. Iridoy M. López P. Jericó I. Lecumberri P. (2021). Longitudinal study in patients with myotonic dystrophy type 1: correlation of brain MRI abnormalities with cognitive performances. *Neuroradiology* 63 1019–1029. 33237431 10.1007/s00234-020-02611-9

[B42] Cabada T. Iridoy M. Jericó I. Lecumberri P. Seijas R. Gargallo A. (2017). Brain involvement in myotonic dystrophy type 1: a morphometric and diffusion tensor imaging study with neuropsychological correlation. *Arch. Clin. Neuropsychol.* 32 401–412. 10.1093/arclin/acx008 28164212

[B43] Caillet-Boudin M. L. Fernandez-Gomez F. J. Tran H. Dhaenens C. M. Buee L. Sergeant N. (2014). Brain pathology in myotonic dystrophy: when tauopathy meets spliceopathy and RNAopathy. *Front. Mol. Neurosci.* 6:57. 10.3389/fnmol.2013.00057 24409116 PMC3885824

[B44] Carpentier C. Ghanem D. Fernandez-Gomez F. J. Jumeau F. Philippe J. V. Freyermuth F. (2014). Tau exon 2 responsive elements deregulated in myotonic dystrophy type I are proximal to exon 2 and synergistically regulated by MBNL1 and MBNL2. *Biochim. Biophys. Acta* 1842 654–664. 10.1016/j.bbadis.2014.01.004 24440524

[B45] Carrell S. T. Carrell E. M. Auerbach D. Pandey S. K. Bennett C. F. Dirksen R. T. (2016). Dmpk gene deletion or antisense knockdown does not compromise cardiac or skeletal muscle function in mice. *Hum. Mol. Genet.* 25 4328–4338. 10.1093/hmg/ddw266 27522499 PMC5291200

[B46] Caso F. Agosta F. Peric S. Rakočevid-Stojanovid V. Copetti M. Kostic V. S. (2014). Cognitive impairment in myotonic dystrophy type 1 is associated with white matter damage. *PLoS One* 9:e104697. 10.1371/journal.pone.0104697 25115999 PMC4130603

[B47] Cerro-Herreros E. Fernandez-Costa J. M. Sabater-Arcis M. Llamusi B. Artero R. (2016). Derepressing muscleblind expression by miRNA sponges ameliorates myotonic dystrophy-like phenotypes in *Drosophila*. *Sci. Rep.* 6:36230. 10.1038/srep36230 27805016 PMC5090246

[B48] Cerro-Herreros E. González-Martínez I. Moreno-Cervera N. Overby S. Pérez-Alonso M. Llamusí B. (2020). Therapeutic potential of AntagomiR-23b for treating myotonic dystrophy. *Mol. Ther. Nucleic Acids* 21 837–849. 10.1016/j.omtn.2020.07.021 32805487 PMC7452101

[B49] Cerro-Herreros E. Sabater-Arcis M. Fernandez-Costa J. M. Moreno N. Perez-Alonso M. Llamusi B. (2018). miR-23b and miR-218 silencing increase Muscleblind-like expression and alleviate myotonic dystrophy phenotypes in mammalian models. *Nat. Commun.* 9:2482. 10.1038/s41467-018-04892-4 29946070 PMC6018771

[B50] Chakraborty M. Sellier C. Ney M. Pascal V. Charlet-Berguerand N. Artero R. (2018). Daunorubicin reduces MBNL1 sequestration caused by CUG-repeat expansion and rescues cardiac dysfunctions in a *Drosophila* model of myotonic dystrophy. *Dis. Models Mech.* 11:dmm032557. 10.1242/dmm.032557 29592894 PMC5963859

[B51] Chang L. Ernst T. Osborn D. Seltzer W. Leonido-Yee M. Poland R. E. (1998). Proton spectroscopy in myotonic dystrophy: correlations with CTG repeats. *Arch. Neurol.* 55 305–311. 10.1001/archneur.55.3.305 9520004

[B52] Charizanis K. Lee K. Y. Batra R. Goodwin M. Zhang C. Yuan Y. (2012). Muscleblind-like 2-mediated alternative splicing in the developing brain and dysregulation in myotonic dystrophy. *Neuron* 75 437–450. 10.1016/j.neuron.2012.05.029 22884328 PMC3418517

[B53] Chau A. Kalsotra A. (2015). Developmental insights into the pathology of and therapeutic strategies for DM1: back to the basics. *Dev. Dyn.* 244 377–390. 10.1002/dvdy.24240 25504326

[B54] Chen G. Carter R. E. Cleary J. D. Reid T. S. Ranum L. P. Swanson M. S. (2018). Altered levels of the splicing factor muscleblind modifies cerebral cortical function in mouse models of myotonic dystrophy. *Neurobiol. Dis.* 112 35–48. 10.1016/j.nbd.2018.01.003 29331264 PMC5859959

[B55] Chen G. Masuda A. Konishi H. Ohkawara B. Ito M. Kinoshita M. (2016). Phenylbutazone induces expression of MBNL1 and suppresses formation of MBNL1-CUG RNA foci in a mouse model of myotonic dystrophy. *Sci. Rep.* 6:25317. 10.1038/srep25317 27126921 PMC4850456

[B56] Childs-Disney J. L. Stepniak-Konieczna E. Tran T. Yildirim I. Park H. Chen C. Z. (2013). Induction and reversal of myotonic dystrophy type 1 pre-mRNA splicing defects by small molecules. *Nat. Commun.* 4:2044. 10.1038/ncomms3044 23806903 PMC3710115

[B57] Chiriboga C. A. Swoboda K. J. Darras B. T. Iannaccone S. T. Montes J. De Vivo D. C. (2016). Results from a phase 1 study of nusinersen (ISIS-SMN(Rx)) in children with spinal muscular atrophy. *Neurology* 86 890–897. 10.1212/wnl.0000000000002445 26865511 PMC4782111

[B58] Çoban A. Bilgiç B. Lohmann E. Küçükali C. Benbir G. Karadeniz D. (2013). Reduced orexin-A levels in frontotemporal dementia: possible association with sleep disturbance. *Am. J. Alzheimers Dis. Dement.* 28 606–611. 10.1177/1533317513494453 23813609 PMC10852656

[B59] Comim C. M. Mathia G. B. Hoepers A. Tuon L. Kapczinski F. Dal-Pizzol F. (2015). Neurotrophins, cytokines, oxidative parameters and funcionality in progressive muscular dystrophies. *Anais Acad. Bras. Ciencias* 87 1809–1818. 10.1590/0001-3765201520140508 25910175

[B60] Conboy J. G. (2017). Developmental regulation of RNA processing by Rbfox proteins. *Wiley Interdiscip. Rev. RNA* 8:1398. 10.1002/wrna.1398 27748060 PMC5315656

[B61] Conforti R. de Cristofaro M. Cristofano A. Brogna B. Sardaro A. Tedeschi G. (2016). Brain MRI abnormalities in the adult form of myotonic dystrophy type 1: a longitudinal case series study. *Neuroradiol. J.* 29 36–45. 10.1177/1971400915621325 26755488 PMC4978343

[B62] Coonrod L. A. Nakamori M. Wang W. Carrell S. Hilton C. L. Bodner M. J. (2013). Reducing levels of toxic RNA with small molecules. *ACS Chem. Biol.* 8 2528–2537. 10.1021/cb400431f 24028068 PMC4108295

[B63] Culebras A. Feldman R. G. Merk F. B. (1973). Cytoplasmic inclusion bodies within neurons of the thalamus in myotonic dystrophy. A light and electron microscope study. *J. Neurol. Sci.* 19 319–329. 10.1016/0022-510x(73)90095-6 4123799

[B64] Czubak K. Sedehizadeh S. Kozlowski P. Wojciechowska M. (2019a). An overview of circular RNAs and their implications in myotonic dystrophy. *Int. J. Mol. Sci.* 20:4385. 10.3390/ijms20184385 31500099 PMC6769675

[B65] Czubak K. Taylor K. Piasecka A. Sobczak K. Kozlowska K. Philips A. (2019b). Global increase in circular RNA levels in myotonic dystrophy. *Front. Genet.* 10:649. 10.3389/fgene.2019.00649 31428124 PMC6689976

[B66] Dagenhardt J. Trinh A. Sumner H. Scott J. Aamodt E. Dwyer D. S. (2017). Insulin signaling deficiency produces immobility in caenorhabditis elegans that models diminished motivation states in man and responds to antidepressants. *Mol. Neuropsychiatry* 3 97–107. 10.1159/000478049 29230398 PMC5701274

[B67] Danos O. (2008). AAV vectors for RNA-based modulation of gene expression. *Gene Ther.* 15 864–869. 10.1038/gt.2008.69 18418414

[B68] Dasgupta T. Ladd A. N. (2012). The importance of CELF control: molecular and biological roles of the CUG-BP, Elav-like family of RNA-binding proteins. *Wiley Interdiscip. Rev. RNA* 3 104–121. 10.1002/wrna.107 22180311 PMC3243963

[B69] De Antonio M. Dogan C. Hamroun D. Mati M. Zerrouki S. Eymard B. (2016). Unravelling the myotonic dystrophy type 1 clinical spectrum: a systematic registry-based study with implications for disease classification. *Revue Neurol.* 172 572–580. 10.1016/j.neurol.2016.08.003 27665240

[B70] de León M. B. Cisneros B. (2008). Myotonic dystrophy 1 in the nervous system: from the clinic to molecular mechanisms. *J. Neurosci. Res.* 86 18–26. 10.1002/jnr.21377 17549748

[B71] Denis J. A. Gauthier M. Rachdi L. Aubert S. Giraud-Triboult K. Poydenot P. (2013). mTOR-dependent proliferation defect in human ES-derived neural stem cells affected by myotonic dystrophy type 1. *J. Cell Sci.* 126(Pt 8), 1763–1772. 10.1242/jcs.116285 23444380

[B72] Dhaenens C. M. Schraen-Maschke S. Tran H. Vingtdeux V. Ghanem D. Leroy O. (2008). Overexpression of MBNL1 fetal isoforms and modified splicing of Tau in the DM1 brain: two individual consequences of CUG trinucleotide repeats. *Exp. Neurol.* 210 467–478. 10.1016/j.expneurol.2007.11.020 18177861

[B73] Dhaenens C. M. Tran H. Frandemiche M. L. Carpentier C. Schraen-Maschke S. Sistiaga A. (2011). Mis-splicing of Tau exon 10 in myotonic dystrophy type 1 is reproduced by overexpression of CELF2 but not by MBNL1 silencing. *Biochim. Biophys. Acta* 1812 732–742. 10.1016/j.bbadis.2011.03.010 21439371

[B74] Di Filippo L. D. Duarte J. Fonseca-Santos B. Tavares Júnior A. G. Araújo V. Roque Borda C. A. (2021). Mucoadhesive nanosystems for nose-to-brain drug delivery in the treatment of central nervous system diseases. *Curr. Med. Chem.* 10.2174/0929867328666210813154019 [Epub ahead of print]. 34391374

[B75] Dietz G. P. Valbuena P. C. Dietz B. Meuer K. Müeller P. Weishaupt J. H. (2006). Application of a blood-brain-barrier-penetrating form of GDNF in a mouse model for Parkinson’s disease. *Brain Res.* 1082 61–66. 10.1016/j.brainres.2006.01.083 16703672

[B76] Douniol M. Jacquette A. Cohen D. Bodeau N. Rachidi L. Angeard N. (2012). Psychiatric and cognitive phenotype of childhood myotonic dystrophy type 1. *Dev. Med. Child Neurol.* 54 905–911. 10.1111/j.1469-8749.2012.04379.x 22861906

[B77] Dragileva E. Hendricks A. Teed A. Gillis T. Lopez E. T. Friedberg E. C. (2009). Intergenerational and striatal CAG repeat instability in Huntington’s disease knock-in mice involve different DNA repair genes. *Neurobiol. Dis.* 33 37–47. 10.1016/j.nbd.2008.09.014 18930147 PMC2811282

[B78] Du J. Campau E. Soragni E. Jespersen C. Gottesfeld J. M. (2013). Length-dependent CTG⋅CAG triplet-repeat expansion in myotonic dystrophy patient-derived induced pluripotent stem cells. *Hum. Mol. Genet.* 22 5276–5287. 10.1093/hmg/ddt386 23933738 PMC3842182

[B79] Echenne B. Rideau A. Roubertie A. Sébire G. Rivier F. Lemieux B. (2008). Myotonic dystrophy type I in childhood Long-term evolution in patients surviving the neonatal period. *Eur. J. Paediatr. Neurol.* 12 210–223. 10.1016/j.ejpn.2007.07.014 17892958

[B80] Ekström A. B. Hakenäs-Plate L. Samuelsson L. Tulinius M. Wentz E. (2008). Autism spectrum conditions in myotonic dystrophy type 1: a study on 57 individuals with congenital and childhood forms. *Am. J. Med. Genet. B Neuropsychiatr. Genet.* 147b 918–926. 10.1002/ajmg.b.30698 18228241

[B81] Fabbri M. Girnita L. Varani G. Calin G. A. (2019). Decrypting noncoding RNA interactions, structures, and functional networks. *Genome Res.* 29 1377–1388. 10.1101/gr.247239.118 31434680 PMC6724670

[B82] Fardaei M. Rogers M. T. Thorpe H. M. Larkin K. Hamshere M. G. Harper P. S. (2002). Three proteins, MBNL, MBLL and MBXL, co-localize in vivo with nuclear foci of expanded-repeat transcripts in DM1 and DM2 cells. *Hum. Mol. Genet.* 11 805–814. 10.1093/hmg/11.7.805 11929853

[B83] Fernandez-Costa J. M. Garcia-Lopez A. Zuñiga S. Fernandez-Pedrosa V. Felipo-Benavent A. Mata M. (2013). Expanded CTG repeats trigger miRNA alterations in Drosophila that are conserved in myotonic dystrophy type 1 patients. *Hum. Mol. Genet.* 22 704–716. 10.1093/hmg/dds478 23139243

[B84] Fernandez-Gomez F. Tran H. Dhaenens C. M. Caillet-Boudin M. L. Schraen-Maschke S. Blum D. (2019). Myotonic dystrophy: an RNA toxic gain of function tauopathy? *Adv. Exp. Med. Biol.* 1184 207–216. 10.1007/978-981-32-9358-8_17 32096040

[B85] Fiorelli M. Duboc D. Mazoyer B. M. Blin J. Eymard B. Fardeau M. (1992). Decreased cerebral glucose utilization in myotonic dystrophy. *Neurology* 42 91–94. 10.1212/wnl.42.1.91 1734329

[B86] Flower M. Lomeikaite V. Ciosi M. Cumming S. Morales F. Lo K. (2019). MSH3 modifies somatic instability and disease severity in Huntington’s and myotonic dystrophy type 1. *Brain* 142 1876–1886. 10.1093/brain/awz115 31216018 PMC6598626

[B87] Foust K. D. Kaspar B. K. (2009). Over the barrier and through the blood: to CNS delivery we go. *Cell Cycle* 8 4017–4018. 10.4161/cc.8.24.10245 19949299 PMC2911437

[B88] Franc D. T. Muetzel R. L. Robinson P. R. Rodriguez C. P. Dalton J. C. Naughton C. E. (2012). Cerebral and muscle MRI abnormalities in myotonic dystrophy. *Neuromusc. Disord.* 22 483–491. 10.1016/j.nmd.2012.01.003 22290140 PMC3350604

[B89] Francois V. Klein A. F. Beley C. Jollet A. Lemercier C. Garcia L. (2011). Selective silencing of mutated mRNAs in DM1 by using modified hU7-snRNAs. *Nat. Struct. Mol. Biol.* 18 85–87. 10.1038/nsmb.1958 21186365

[B90] Fritegotto C. Ferrati C. Pegoraro V. Angelini C. (2017). Micro-RNA expression in muscle and fiber morphometry in myotonic dystrophy type 1. *Neurol. Sci.* 38 619–625. 10.1007/s10072-017-2811-2 28078570

[B91] Fu Y. H. Pizzuti A. Fenwick R. G. Jr. King J. Rajnarayan S. Dunne P. W. (1992). An unstable triplet repeat in a gene related to myotonic muscular dystrophy. *Science* 255 1256–1258. 10.1126/science.1546326 1546326

[B92] Furling D. Doucet G. Langlois M. A. Timchenko L. Belanger E. Cossette L. (2003). Viral vector producing antisense RNA restores myotonic dystrophy myoblast functions. *Gene Ther.* 10 795–802. 10.1038/sj.gt.3301955 12704419

[B93] Furuta M. Kimura T. Nakamori M. Matsumura T. Fujimura H. Jinnai K. (2018). Macroscopic and microscopic diversity of missplicing in the central nervous system of patients with myotonic dystrophy type 1. *Neuroreport* 29 235–240. 10.1097/wnr.0000000000000968 29381654

[B94] Gadgil A. Raczyńska K. D. (2021). U7 snRNA: a tool for gene therapy. *J. Gene Med.* 23:e3321. 10.1002/jgm.3321 33590603 PMC8243935

[B95] Gallais B. Gagnon C. Mathieu J. Richer L. (2017). Cognitive decline over time in adults with myotonic dystrophy type 1: a 9-year longitudinal study. *Neuromusc. Disord.* 27 61–72. 10.1016/j.nmd.2016.10.003 27919548

[B96] Gallais B. Montreuil M. Gargiulo M. Eymard B. Gagnon C. Laberge L. (2015). Prevalence and correlates of apathy in myotonic dystrophy type 1. *BMC Neurol.* 15:148. 10.1186/s12883-015-0401-6 26296336 PMC4546188

[B97] Gambardella S. Rinaldi F. Lepore S. M. Viola A. Loro E. Angelini C. (2010). Overexpression of microRNA-206 in the skeletal muscle from myotonic dystrophy type 1 patients. *J. Transl. Med.* 8:48. 10.1186/1479-5876-8-48 20487562 PMC2880982

[B98] Gao Y. Guo X. Santostefano K. Wang Y. Reid T. Zeng D. (2016). Genome therapy of myotonic dystrophy type 1 ips cells for development of autologous stem cell therapy. *Mol. Ther.* 24 1378–1387. 10.1038/mt.2016.97 27203440 PMC5023370

[B99] García-López A. Llamusí B. Orzáez M. Pérez-Payá E. Artero R. D. (2011). In vivo discovery of a peptide that prevents CUG-RNA hairpin formation and reverses RNA toxicity in myotonic dystrophy models. *Proc. Natl. Acad. Sci. U.S.A.* 108 11866–11871. 10.1073/pnas.1018213108 21730182 PMC3141925

[B100] García-Puga M. Saenz-Antoñanzas A. Fernández-Torrón R. Munain A. L. Matheu A. (2020). Myotonic Dystrophy type 1 cells display impaired metabolism and mitochondrial dysfunction that are reversed by metformin. *Aging* 12 6260–6275. 10.18632/aging.103022 32310829 PMC7185118

[B101] Gaudelli N. M. Komor A. C. Rees H. A. Packer M. S. Badran A. H. Bryson D. I. (2017). Programmable base editing of A•T to G•C in genomic DNA without DNA cleavage. *Nature* 551 464–471. 10.1038/nature24644 29160308 PMC5726555

[B102] Geary R. S. Norris D. Yu R. Bennett C. F. (2015). Pharmacokinetics, biodistribution and cell uptake of antisense oligonucleotides. *Adv. Drug Deliv. Rev.* 87 46–51. 10.1016/j.addr.2015.01.008 25666165

[B103] Gehman L. T. Stoilov P. Maguire J. Damianov A. Lin C. H. Shiue L. (2011). The splicing regulator Rbfox1 (A2BP1) controls neuronal excitation in the mammalian brain. *Nat. Genet.* 43 706–711. 10.1038/ng.841 21623373 PMC3125461

[B104] Glantz R. H. Wright R. B. Huckman M. S. Garron D. C. Siegel I. M. (1988). Central nervous system magnetic resonance imaging findings in myotonic dystrophy. *Arch. Neurol.* 45 36–37. 10.1001/archneur.1988.00520250042017 3337674

[B105] Gonçalves R. A. Wijesekara N. Fraser P. E. De Felice F. G. (2019). The link between tau and insulin signaling: implications for Alzheimer’s disease and other tauopathies. *Front. Cell. Neurosci.* 13:17. 10.3389/fncel.2019.00017 30804755 PMC6371747

[B106] González-Barriga A. Lallemant L. Dincã D. M. Braz S. O. Polvèche H. Magneron P. (2021). Integrative cell type-specific multi-omics approaches reveal impaired programs of glial cell differentiation in mouse culture models of DM1. *Front. Cell. Neurosci.* 15:662035. 10.3389/fncel.2021.662035 34025359 PMC8136287

[B107] Goodwin M. Mohan A. Batra R. Lee K. Y. Charizanis K. Fernández Gómez F. J. (2015). MBNL sequestration by toxic RNAs and RNA misprocessing in the myotonic dystrophy brain. *Cell Rep.* 12 1159–1168. 10.1016/j.celrep.2015.07.029 26257173 PMC4545389

[B108] Gregorevic P. Blankinship M. J. Allen J. M. Crawford R. W. Meuse L. Miller D. G. (2004). Systemic delivery of genes to striated muscles using adeno-associated viral vectors. *Nat. Med.* 10 828–834. 10.1038/nm1085 15273747 PMC1365046

[B109] Groenen P. J. Wansink D. G. Coerwinkel M. van den Broek W. Jansen G. Wieringa B. (2000). Constitutive and regulated modes of splicing produce six major myotonic dystrophy protein kinase (DMPK) isoforms with distinct properties. *Hum. Mol. Genet.* 9 605–616. 10.1093/hmg/9.4.605 10699184

[B110] Gudde A. van Heeringen S. J. de Oude A. I. van Kessel I. D. G. Estabrook J. Wang E. T. (2017). Antisense transcription of the myotonic dystrophy locus yields low-abundant RNAs with and without (CAG)n repeat. *RNA Biol.* 14 1374–1388. 10.1080/15476286.2017.1279787 28102759 PMC5711456

[B111] Gudde A. E. González-Barriga A. van den Broek W. J. Wieringa B. Wansink D. G. (2016). A low absolute number of expanded transcripts is involved in myotonic dystrophy type 1 manifestation in muscle. *Hum. Mol. Genet.* 25 1648–1662. 10.1093/hmg/ddw042 26908607 PMC4805313

[B112] Han S. Nam J. Li Y. Kim S. Cho S. H. Cho Y. S. (2010). Regulation of dendritic spines, spatial memory, and embryonic development by the TANC family of PSD-95-interacting proteins. *J. Neurosci.* 30 15102–15112. 10.1523/jneurosci.3128-10.2010 21068316 PMC6633848

[B113] Harper P. S. van Engelen B. G. Eymard B. Rogers M. Wilcox D. (2002). 99th ENMC international workshop: myotonic dystrophy: present management, future therapy. 9-11 November 2001, Naarden, The Netherlands. *Neuromusc. Disord.* 12 596–599. 10.1016/s0960-8966(02)00020-2 12117486

[B114] Hashimoto T. Tayama M. Miyazaki M. Murakawa K. Kawai H. Nishitani H. (1995). Neuroimaging study of myotonic dystrophy. II. MRI measurements of the brain. *Brain Dev.* 17 28–32. 10.1016/0387-7604(94)00097-h 7762759

[B115] Heatwole C. Bode R. Johnson N. Quinn C. Martens W. McDermott M. P. (2012). Patient-reported impact of symptoms in myotonic dystrophy type 1 (PRISM-1). *Neurology* 79 348–357. 10.1212/WNL.0b013e318260cbe6 22786587 PMC3400095

[B116] Heatwole C. Bode R. Johnson N. E. Dekdebrun J. Dilek N. Eichinger K. (2016). Myotonic dystrophy health index: correlations with clinical tests and patient function. *Muscle Nerve* 53 183–190. 10.1002/mus.24725 26044513 PMC4979973

[B117] Heatwole C. Johnson N. Dekdebrun J. Dilek N. Eichinger K. Hilbert J. (2018). Myotonic dystrophy patient preferences in patient-reported outcome measures. *Muscle Nerve* 10.1002/mus.26066 [Epub ahead of print]. 29328504

[B118] Heatwole C. Luebbe E. Rosero S. Eichinger K. Martens W. Hilbert J. (2021). Mexiletine in myotonic dystrophy type 1: a randomized, double-blind, placebo-controlled trial. *Neurology* 96 e228–e240. 10.1212/wnl.0000000000011002 33046619 PMC7905778

[B119] Hermans M. C. Hoeijmakers J. G. Faber C. G. Merkies I. S. (2015). Reconstructing the rasch-built myotonic dystrophy type 1 activity and participation scale. *PLoS One* 10:e0139944. 10.1371/journal.pone.0139944 26484877 PMC4618741

[B120] Hermans M. C. Merkies I. S. Laberge L. Blom E. W. Tennant A. Faber C. G. (2013). Fatigue and daytime sleepiness scale in myotonic dystrophy type 1. *Muscle Nerve* 47 89–95. 10.1002/mus.23478 23042586

[B121] Hernández-Hernández O. Guiraud-Dogan C. Sicot G. Huguet A. Luilier S. Steidl E. (2013a). Myotonic dystrophy CTG expansion affects synaptic vesicle proteins, neurotransmission and mouse behaviour. *Brain* 136(Pt 3), 957–970. 10.1093/brain/aws367 23404338 PMC3580270

[B122] Hernández-Hernández O. Sicot G. Dinca D. M. Huguet A. Nicole A. Buée L. (2013b). Synaptic protein dysregulation in myotonic dystrophy type 1: disease neuropathogenesis beyond missplicing. *Rare Dis.* 1:e25553. 10.4161/rdis.25553 25003003 PMC3927487

[B123] Hernando S. Herran E. Figueiro-Silva J. Pedraz J. L. Igartua M. Carro E. (2018). Intranasal administration of TAT-conjugated lipid nanocarriers loading GDNF for Parkinson’s disease. *Mol. Neurobiol.* 55 145–155. 10.1007/s12035-017-0728-7 28866799

[B124] Hilton-Jones D. Bowler M. Lochmueller H. Longman C. Petty R. Roberts M. (2012). Modafinil for excessive daytime sleepiness in myotonic dystrophy type 1–the patients’ perspective. *Neuromusc. Disord.* 22 597–603. 10.1016/j.nmd.2012.02.005 22425060

[B125] Hinrich A. J. Jodelka F. M. Chang J. L. Brutman D. Bruno A. M. Briggs C. A. (2016). Therapeutic correction of ApoER2 splicing in Alzheimer’s disease mice using antisense oligonucleotides. *EMBO Mol. Med.* 8 328–345. 10.15252/emmm.201505846 26902204 PMC4818756

[B126] Hirase T. Araki S. (1984). Cerebrospinal fluid proteins in muscular dystrophy patients. *Brain Dev.* 6 10–16. 10.1016/s0387-7604(84)80003-0 6203422

[B127] Horrigan J. Gomes T. B. Snape M. Nikolenko N. McMorn A. Evans S. (2020). A phase 2 study of AMO-02 (Tideglusib) in congenital and childhood-onset myotonic dystrophy type 1 (DM1). *Pediatr. Neurol.* 112 84–93. 10.1016/j.pediatrneurol.2020.08.001 32942085

[B128] Hoskins J. W. Ofori L. O. Chen C. Z. Kumar A. Sobczak K. Nakamori M. (2014). Lomofungin and dilomofungin: inhibitors of MBNL1-CUG RNA binding with distinct cellular effects. *Nucleic Acids Res.* 42 6591–6602. 10.1093/nar/gku275 24799433 PMC4041448

[B129] Hsieh W. C. Bahal R. Thadke S. A. Bhatt K. Sobczak K. Thornton C. (2018). Design of a “mini” nucleic acid probe for cooperative binding of an RNA-repeated transcript associated with myotonic dystrophy type 1. *Biochemistry* 57 907–911. 10.1021/acs.biochem.7b01239 29334465 PMC6091549

[B130] Hu N. Kim E. Antoury L. Li J. González-Pérez P. Rutkove S. B. (2021). Antisense oligonucleotide and adjuvant exercise therapy reverse fatigue in old mice with myotonic dystrophy. *Mol. Ther. Nucleic Acids* 23 393–405. 10.1016/j.omtn.2020.11.014 33473325 PMC7787993

[B131] Hua Y. Sahashi K. Rigo F. Hung G. Horev G. Bennett C. F. (2011). Peripheral SMN restoration is essential for long-term rescue of a severe spinal muscular atrophy mouse model. *Nature* 478 123–126. 10.1038/nature10485 21979052 PMC3191865

[B132] Huguet A. Medja F. Nicole A. Vignaud A. Guiraud-Dogan C. Ferry A. (2012). Molecular, physiological, and motor performance defects in DMSXL mice carrying >1,000 CTG repeats from the human DM1 locus. *PLoS Genet.* 8:e1003043. 10.1371/journal.pgen.1003043 23209425 PMC3510028

[B133] Itoh K. Mitani M. Kawamoto K. Futamura N. Funakawa I. Jinnai K. (2010). Neuropathology does not correlate with regional differences in the extent of expansion of CTG repeats in the brain with myotonic dystrophy type 1. *Acta Histochem. Cytochem.* 43 149–156. 10.1267/ahc.10019 21245981 PMC3015052

[B134] Jacko M. Weyn-Vanhentenryck S. M. Smerdon J. W. Yan R. Feng H. Williams D. J. (2018). Rbfox splicing factors promote neuronal maturation and axon initial segment assembly. *Neuron* 97 853.e6–868.e6. 10.1016/j.neuron.2018.01.020 29398366 PMC5823762

[B135] Jauvin D. Chretien J. Pandey S. K. Martineau L. Revillod L. Bassez G. (2017). Targeting DMPK with Antisense Oligonucleotide Improves Muscle Strength in Myotonic Dystrophy Type 1 Mice. *Mol. Ther. Nucleic Acids* 7 465–474. 10.1016/j.omtn.2017.05.007 28624222 PMC5453865

[B136] Jean S. Richer L. Laberge L. Mathieu J. (2014). Comparisons of intellectual capacities between mild and classic adult-onset phenotypes of myotonic dystrophy type 1 (DM1). *Orphanet J. Rare Dis.* 9:186. 10.1186/s13023-014-0186-5 25424323 PMC4247010

[B137] Jenquin J. R. Coonrod L. A. Silverglate Q. A. Pellitier N. A. Hale M. A. Xia G. (2018). Furamidine rescues myotonic dystrophy type I associated mis-splicing through multiple mechanisms. *ACS Chem. Biol.* 13 2708–2718. 10.1021/acschembio.8b00646 30118588 PMC6343479

[B138] Jenquin J. R. Yang H. Huigens R. W. III Nakamori M. Berglund J. A. (2019). Combination treatment of erythromycin and furamidine provides additive and synergistic rescue of mis-splicing in myotonic dystrophy type 1 models. *ACS Pharmacol. Transl. Sci.* 2 247–263. 10.1021/acsptsci.9b00020 31485578 PMC6726129

[B139] Jiang H. Mankodi A. Swanson M. S. Moxley R. T. Thornton C. A. (2004). Myotonic dystrophy type 1 is associated with nuclear foci of mutant RNA, sequestration of muscleblind proteins and deregulated alternative splicing in neurons. *Hum. Mol. Genet.* 13 3079–3088. 10.1093/hmg/ddh327 15496431

[B140] Jimenez-Marin A. Diez I. Labayru G. Sistiaga A. Caballero M. C. Andres-Benito P. (2021). Transcriptional signatures of synaptic vesicle genes define myotonic dystrophy type I neurodegeneration. *Neuropathol. Appl. Neurobiol.* 10.1111/nan.12725 [Epub ahead of print]. 33955002 PMC9292638

[B141] Jinnai K. Mitani M. Futamura N. Kawamoto K. Funakawa I. Itoh K. (2013). Somatic instability of CTG repeats in the cerebellum of myotonic dystrophy type 1. *Muscle Nerve* 48 105–108. 10.1002/mus.23717 23629807

[B142] Jones K. Wei C. Iakova P. Bugiardini E. Schneider-Gold C. Meola G. (2012). GSK3β mediates muscle pathology in myotonic dystrophy. *J. Clin. Investig.* 122 4461–4472. 10.1172/jci64081 23160194 PMC3533547

[B143] Kalsotra A. Singh R. K. Gurha P. Ward A. J. Creighton C. J. Cooper T. A. (2014). The Mef2 transcription network is disrupted in myotonic dystrophy heart tissue, dramatically altering miRNA and mRNA expression. *Cell Rep.* 6 336–345. 10.1016/j.celrep.2013.12.025 24412363 PMC3927417

[B144] Kamei N. Takeda-Morishita M. (2015). Brain delivery of insulin boosted by intranasal coadministration with cell-penetrating peptides. *J. Control. Release* 197 105–110. 10.1016/j.jconrel.2014.11.004 25445695

[B145] Kanadia R. N. Shin J. Yuan Y. Beattie S. G. Wheeler T. M. Thornton C. A. (2006). Reversal of RNA missplicing and myotonia after muscleblind overexpression in a mouse poly(CUG) model for myotonic dystrophy. *Proc. Natl. Acad. Sci. U.S.A.* 103 11748–11753. 10.1073/pnas.0604970103 16864772 PMC1544241

[B146] Ketley A. Chen C. Z. Li X. Arya S. Robinson T. E. Granados-Riveron J. (2014). High-content screening identifies small molecules that remove nuclear foci, affect MBNL distribution and CELF1 protein levels via a PKC-independent pathway in myotonic dystrophy cell lines. *Hum. Mol. Genet.* 23 1551–1562. 10.1093/hmg/ddt542 24179176 PMC3929092

[B147] Khafagy E. S. Kamei N. Fujiwara Y. Okumura H. Yuasa T. Kato M. (2020). Systemic and brain delivery of leptin via intranasal coadministration with cell-penetrating peptides and its therapeutic potential for obesity. *J. Control. Release* 319 397–406. 10.1016/j.jconrel.2020.01.016 31926192

[B148] Khorkova O. Wahlestedt C. (2017). Oligonucleotide therapies for disorders of the nervous system. *Nat. Biotechnol.* 35 249–263. 10.1038/nbt.3784 28244991 PMC6043900

[B149] Kilic U. Kilic E. Dietz G. P. Bähr M. (2003). Intravenous TAT-GDNF is protective after focal cerebral ischemia in mice. *Stroke* 34 1304–1310. 10.1161/01.str.0000066869.45310.50 12677018

[B150] Kilic U. Kilic E. Dietz G. P. Bähr M. (2004). The TAT protein transduction domain enhances the neuroprotective effect of glial-cell-line-derived neurotrophic factor after optic nerve transection. *Neuro Degener. Dis.* 1 44–49. 10.1159/000076669 16908973

[B151] Kim K. Lee S. G. Kegelman T. P. Su Z. Z. Das S. K. Dash R. (2011). Role of excitatory amino acid transporter-2 (EAAT2) and glutamate in neurodegeneration: opportunities for developing novel therapeutics. *J. Cell. Physiol.* 226 2484–2493. 10.1002/jcp.22609 21792905 PMC3130100

[B152] Klein A. F. Dastidar S. Furling D. Chuah M. K. (2015). Therapeutic approaches for dominant muscle diseases: highlight on myotonic dystrophy. *Curr. Gene Ther.* 15 329–337. 10.2174/1566523215666150630120537 26122101

[B153] Klein A. F. Varela M. A. Arandel L. Holland A. Naouar N. Arzumanov A. (2019). Peptide-conjugated oligonucleotides evoke long-lasting myotonic dystrophy correction in patient-derived cells and mice. *J. Clin. Investig.* 129 4739–4744. 10.1172/jci128205 31479430 PMC6819114

[B154] Klinck R. Fourrier A. Thibault P. Toutant J. Durand M. Lapointe E. (2014). RBFOX1 cooperates with MBNL1 to control splicing in muscle, including events altered in myotonic dystrophy type 1. *PLoS One* 9:e107324. 10.1371/journal.pone.0107324 25211016 PMC4161394

[B155] Knott G. J. Doudna J. A. C. R. I. S. P. R. - (2018). Cas guides the future of genetic engineering. *Science* 361 866–869. 10.1126/science.aat5011 30166482 PMC6455913

[B156] Kobayakawa M. Tsuruya N. Kawamura M. (2012). Theory of mind impairment in adult-onset myotonic dystrophy type 1. *Neurosci. Res.* 72 341–346. 10.1016/j.neures.2012.01.005 22326781

[B157] Kobayakawa M. Tsuruya N. Takeda A. Suzuki A. Kawamura M. (2010). Facial emotion recognition and cerebral white matter lesions in myotonic dystrophy type 1. *J. Neurol. Sci.* 290 48–51. 10.1016/j.jns.2009.11.011 20006353

[B158] Koebis M. Kiyatake T. Yamaura H. Nagano K. Higashihara M. Sonoo M. (2013). Ultrasound-enhanced delivery of morpholino with Bubble liposomes ameliorates the myotonia of myotonic dystrophy model mice. *Sci. Rep.* 3:2242. 10.1038/srep02242 23873129 PMC3718203

[B159] Koehorst E. Ballester-Lopez A. Arechavala-Gomeza V. Martínez-Piñeiro A. Nogales-Gadea G. (2020). The biomarker potential of miRNAs in myotonic dystrophy type I. *J. Clin. Med.* 9:3939. 10.3390/jcm9123939 33291833 PMC7762003

[B160] Komor A. C. Kim Y. B. Packer M. S. Zuris J. A. Liu D. R. (2016). Programmable editing of a target base in genomic DNA without double-stranded DNA cleavage. *Nature* 533 420–424. 10.1038/nature17946 27096365 PMC4873371

[B161] Kouki T. Takasu N. Nakachi A. Tamanaha T. Komiya I. Tawata M. (2005). Low-dose metformin improves hyperglycaemia related to myotonic dystrophy. *Diabetic Med.* 22 346–347. 10.1111/j.1464-5491.2005.01432.x 15717887

[B162] Koutalianos D. Koutsoulidou A. Mastroyiannopoulos N. P. Furling D. Phylactou L. A. (2015). MyoD transcription factor induces myogenesis by inhibiting Twist-1 through miR-206. *J. Cell Sci.* 128 3631–3645. 10.1242/jcs.172288 26272918

[B163] Koutsoulidou A. Kyriakides T. C. Papadimas G. K. Christou Y. Kararizou E. Papanicolaou E. Z. (2015). Elevated muscle-specific miRNAs in serum of myotonic dystrophy patients relate to muscle disease progress. *PLoS One* 10:e0125341. 10.1371/journal.pone.0125341 25915631 PMC4411125

[B164] Koutsoulidou A. Photiades M. Kyriakides T. C. Georgiou K. Prokopi M. Kapnisis K. (2017). Identification of exosomal muscle-specific miRNAs in serum of myotonic dystrophy patients relating to muscle disease progress. *Hum. Mol. Genet.* 26 3285–3302. 10.1093/hmg/ddx212 28637233

[B165] Kristensen M. Birch D. Mørck Nielsen H. (2016). Applications and challenges for use of cell-penetrating peptides as delivery vectors for peptide and protein cargos. *Int. J. Mol. Sci.* 17:185. 10.3390/ijms17020185 26840305 PMC4783919

[B166] Krogias C. Bellenberg B. Prehn C. Schneider R. Meves S. H. Gold R. (2015). Evaluation of CNS involvement in myotonic dystrophy type 1 and type 2 by transcranial sonography. *J. Neurol.* 262 365–374. 10.1007/s00415-014-7566-6 25385052

[B167] Krogias C. Walter U. (2016). Transcranial sonography findings in depression in association with psychiatric and neurologic diseases: a review. *J. Neuroimaging* 26 257–263. 10.1111/jon.12328 27119431

[B168] Krupa P. Řehák S. Diaz-Garcia D. Filip S. (2014). Nanotechnology - new trends in the treatment of brain tumours. *Acta Med.* 57 142–150. 10.14712/18059694.2015.79 25938897

[B169] Kullmann S. Heni M. Hallschmid M. Fritsche A. Preissl H. Häring H. U. (2016). Brain insulin resistance at the crossroads of metabolic and cognitive disorders in humans. *Physiol. Rev.* 96 1169–1209. 10.1152/physrev.00032.2015 27489306

[B170] Kumar V. B. Farr S. A. Flood J. F. Kamlesh V. Franko M. Banks W. A. (2000). Site-directed antisense oligonucleotide decreases the expression of amyloid precursor protein and reverses deficits in learning and memory in aged SAMP8 mice. *Peptides* 21 1769–1775. 10.1016/s0196-9781(00)00339-9 11150636

[B171] Kuyumcu-Martinez N. M. Wang G. S. Cooper T. A. (2007). Increased steady-state levels of CUGBP1 in myotonic dystrophy 1 are due to PKC-mediated hyperphosphorylation. *Mol. Cell* 28 68–78. 10.1016/j.molcel.2007.07.027 17936705 PMC2083558

[B172] Labayru G. Arenzana I. Aliri J. Zulaica M. López de Munain A. Sistiaga A. A. (2018). Social cognition in myotonic dystrophy type 1: specific or secondary impairment? *PLoS One* 13:e0204227. 10.1371/journal.pone.0204227 30248121 PMC6152965

[B173] Labayru G. Diez I. Sepulcre J. Fernández E. Zulaica M. Cortés J. M. (2019). Regional brain atrophy in gray and white matter is associated with cognitive impairment in Myotonic Dystrophy type 1. *Neuroimage Clin.* 24:102078. 10.1016/j.nicl.2019.102078 31795042 PMC6861566

[B174] Labayru G. Jimenez-Marin A. Fernández E. Villanua J. Zulaica M. Cortes J. M. (2020). Neurodegeneration trajectory in pediatric and adult/late DM1: a follow-up MRI study across a decade. *Ann. Clin. Transl. Neurol.* 7 1802–1815. 10.1002/acn3.51163 32881379 PMC7545612

[B175] Laberge L. Gagnon C. Dauvilliers Y. (2013). Daytime sleepiness and myotonic dystrophy. *Curr. Neurol. Neurosci. Rep.* 13:340. 10.1007/s11910-013-0340-9 23430686

[B176] Ladd A. N. (2013). CUG-BP, Elav-like family (CELF)-mediated alternative splicing regulation in the brain during health and disease. *Mol. Cell. Neurosci.* 56 456–464. 10.1016/j.mcn.2012.12.003 23247071 PMC3650117

[B177] Ladd A. N. Charlet N. Cooper T. A. (2001). The CELF family of RNA binding proteins is implicated in cell-specific and developmentally regulated alternative splicing. *Mol. Cell. Biol.* 21 1285–1296. 10.1128/mcb.21.4.1285-1296.2001 11158314 PMC99581

[B178] Ladd A. N. Nguyen N. H. Malhotra K. Cooper T. A. (2004). CELF6, a member of the CELF family of RNA-binding proteins, regulates muscle-specific splicing enhancer-dependent alternative splicing. *J. Biol. Chem.* 279 17756–17764. 10.1074/jbc.M310687200 14761971

[B179] Langbehn K. E. van der Plas E. Moser D. J. Long J. D. Gutmann L. Nopoulos P. C. (2021). Cognitive function and its relationship with brain structure in myotonic dystrophy type 1. *J. Neurosci. Res.* 99 190–199. 10.1002/jnr.24595 32056295 PMC7872331

[B180] Larsen J. Pettersson O. J. Jakobsen M. Thomsen R. Pedersen C. B. Hertz J. M. (2011). Myoblasts generated by lentiviral mediated MyoD transduction of myotonic dystrophy type 1 (DM1) fibroblasts can be used for assays of therapeutic molecules. *BMC Res. Notes* 4:490. 10.1186/1756-0500-4-490 22078098 PMC3226528

[B181] Laustriat D. Gide J. Barrault L. Chautard E. Benoit C. Auboeuf D. (2015). *In vitro* and *In vivo* modulation of alternative splicing by the biguanide metformin. *Mol. Ther. Nucleic Acids* 4:e262. 10.1038/mtna.2015.35 26528939 PMC4877444

[B182] Le Hir M. Goyenvalle A. Peccate C. Précigout G. Davies K. E. Voit T. (2013). AAV genome loss from dystrophic mouse muscles during AAV-U7 snRNA-mediated exon-skipping therapy. *Mol. Ther.* 21 1551–1558. 10.1038/mt.2013.121 23752313 PMC3734654

[B183] Lebleu B. Moulton H. M. Abes R. Ivanova G. D. Abes S. Stein D. A. (2008). Cell penetrating peptide conjugates of steric block oligonucleotides. *Adv. Drug Deliv. Rev.* 60 517–529. 10.1016/j.addr.2007.09.002 18037527 PMC7103303

[B184] Leddy S. Serra L. Esposito D. Vizzotto C. Giulietti G. Silvestri G. (2021). Lesion distribution and substrate of white matter damage in myotonic dystrophy type 1: comparison with multiple sclerosis. *Neuroimage Clin.* 29:102562. 10.1016/j.nicl.2021.102562 33516936 PMC7848627

[B185] Lee H. B. Sundberg B. N. Sigafoos A. N. Clark K. J. (2016). Genome engineering with TALE and CRISPR systems in neuroscience. *Front. Genet.* 7:47. 10.3389/fgene.2016.00047 27092173 PMC4821859

[B186] Lee J. E. Bennett C. F. Cooper T. A. (2012). RNase H-mediated degradation of toxic RNA in myotonic dystrophy type 1. *Proc. Natl. Acad. Sci. U.S.A.* 109 4221–4226. 10.1073/pnas.1117019109 22371589 PMC3306674

[B187] Lee K. Conboy M. Park H. M. Jiang F. Kim H. J. Dewitt M. A. (2017). Nanoparticle delivery of Cas9 ribonucleoprotein and donor DNA in vivo induces homology-directed DNA repair. *Nat. Biomed. Eng.* 1 889–901. 10.1038/s41551-017-0137-2 29805845 PMC5968829

[B188] Lee K. Y. Chang H. C. Seah C. Lee L. J. (2019). Deprivation of muscleblind-like proteins causes deficits in cortical neuron distribution and morphological changes in dendritic spines and postsynaptic densities. *Front. Neuroanat.* 13:75. 10.3389/fnana.2019.00075 31417371 PMC6682673

[B189] Lee K. Y. Li M. Manchanda M. Batra R. Charizanis K. Mohan A. (2013). Compound loss of muscleblind-like function in myotonic dystrophy. *EMBO Mol. Med.* 5 1887–1900. 10.1002/emmm.201303275 24293317 PMC3914532

[B190] Lehto T. Kurrikoff K. Langel Ü (2012). Cell-penetrating peptides for the delivery of nucleic acids. *Expert Opin. Drug Deliv.* 9 823–836. 10.1517/17425247.2012.689285 22594635

[B191] Leroy O. Dhaenens C. M. Schraen-Maschke S. Belarbi K. Delacourte A. Andreadis A. (2006a). ETR-3 represses Tau exons 2/3 inclusion, a splicing event abnormally enhanced in myotonic dystrophy type I. *J. Neurosci. Res.* 84 852–859. 10.1002/jnr.20980 16862542

[B192] Leroy O. Wang J. Maurage C. A. Parent M. Cooper T. Buée L. (2006b). Brain-specific change in alternative splicing of Tau exon 6 in myotonic dystrophy type 1. *Biochim. Biophys. Acta* 1762 460–467. 10.1016/j.bbadis.2005.12.003 16487687

[B193] Lim J. J. Derby M. A. Zhang Y. Deng R. Larouche R. Anderson M. (2017). A phase 1, randomized, double-blind, placebo-controlled, single-ascending-dose study to investigate the safety, tolerability, and pharmacokinetics of an anti-influenza B virus monoclonal antibody, MHAB5553A, in healthy volunteers. *Antimicrob. Agents Chemother.* 61:e00279–17. 10.1128/aac.00279-17 28559255 PMC5527589

[B194] Lindeblad G. Kroksmark A. K. Ekström A. B. (2019). Cognitive and adaptive functioning in congenital and childhood forms of myotonic dystrophy type 1: a longitudinal study. *Dev. Med. Child Neurol.* 61 1214–1220. 10.1111/dmcn.14161 30706460

[B195] Lo Scrudato M. Poulard K. Sourd C. Tomé S. Klein A. F. Corre G. (2019). Genome editing of expanded CTG repeats within the human DMPK gene reduces nuclear RNA foci in the muscle of DM1 mice. *Mol. Ther.* 27 1372–1388. 10.1016/j.ymthe.2019.05.021 31253581 PMC6697452

[B196] Logigian E. L. Martens W. B. Moxley RTt McDermott M. P. Dilek N. Wiegner A. W. (2010). Mexiletine is an effective antimyotonia treatment in myotonic dystrophy type 1. *Neurology* 74 1441–1448. 10.1212/WNL.0b013e3181dc1a3a 20439846 PMC2871004

[B197] Long C. Amoasii L. Bassel-Duby R. Olson E. N. (2016). Genome editing of monogenic neuromuscular diseases: a systematic review. *JAMA Neurol.* 73 1349–1355. 10.1001/jamaneurol.2016.3388 27668807 PMC5695221

[B198] López Castel A. Overby S. J. Artero R. (2019). MicroRNA-based therapeutic perspectives in myotonic dystrophy. *Int. J. Mol. Sci.* 20:5600. 10.3390/ijms20225600 31717488 PMC6888406

[B199] López-Morató M. Brook J. D. Wojciechowska M. (2018). Small molecules which improve pathogenesis of myotonic dystrophy type 1. *Front. Neurol.* 9:349. 10.3389/fneur.2018.00349 29867749 PMC5968088

[B200] Lopez-Titla M. M. Chirino A. Cruz Solis S. V. Hernandez-Castillo C. R. Diaz R. Márquez-Quiroz L. D. C. (2021). Cognitive decline and white matter integrity degradation in myotonic dystrophy type I. *J. Neuroimaging* 31 192–198. 10.1111/jon.12786 32936994

[B201] Luu L. M. Nguyen L. Peng S. Lee J. Lee H. Y. Wong C. H. (2016). A potent inhibitor of protein sequestration by expanded triplet (CUG) repeats that shows phenotypic improvements in a drosophila model of myotonic dystrophy. *Chem. Med. Chem.* 11 1428–1435. 10.1002/cmdc.201600081 27245480 PMC5074844

[B202] Magaña J. J. Cisneros B. (2011). Perspectives on gene therapy in myotonic dystrophy type 1. *J. Neurosci. Res.* 89 275–285. 10.1002/jnr.22551 21259315

[B203] Mahadevan M. Tsilfidis C. Sabourin L. Shutler G. Amemiya C. Jansen G. (1992). Myotonic dystrophy mutation: an unstable CTG repeat in the 3′ untranslated region of the gene. *Science* 255 1253–1255. 10.1126/science.1546325 1546325

[B204] Manning K. S. Rao A. N. Castro M. Cooper T. A. (2017). BNA (NC) gapmers revert splicing and reduce RNA foci with low toxicity in myotonic dystrophy cells. *ACS Chem. Biol.* 12 2503–2509. 10.1021/acschembio.7b00416 28853853 PMC5694563

[B205] Marteyn A. Maury Y. Gauthier M. M. Lecuyer C. Vernet R. Denis J. A. (2011). Mutant human embryonic stem cells reveal neurite and synapse formation defects in type 1 myotonic dystrophy. *Cell Stem Cell* 8 434–444. 10.1016/j.stem.2011.02.004 21458401

[B206] Martínez-Rodríguez J. E. Lin L. Iranzo A. Genis D. Martí M. J. Santamaria J. (2003). Decreased hypocretin-1 (Orexin-A) levels in the cerebrospinal fluid of patients with myotonic dystrophy and excessive daytime sleepiness. *Sleep* 26 287–290. 10.1093/sleep/26.3.287 12749547

[B207] Matynia A. Ng C. H. Dansithong W. Chiang A. Silva A. J. Reddy S. (2010). Muscleblind1, but not Dmpk or Six5, contributes to a complex phenotype of muscular and motivational deficits in mouse models of myotonic dystrophy. *PLoS One* 5:e9857. 10.1371/journal.pone.0009857 20360842 PMC2845609

[B208] Mazzoli M. Ariatti A. Garuti G. C. Agnoletto V. Genovese M. Gozzi M. (2020). Predictors of prognosis in type 1 myotonic dystrophy (DM1): longitudinal 18-years experience from a single center. *Acta Myol.* 39 109–120. 10.36185/2532-1900-015 33305167 PMC7711325

[B209] McGowan J. W. Bidwell G. L. III Vig P. J. (2015). Challenges and new strategies for therapeutic peptide delivery to the CNS. *Ther. Deliv.* 6 841–853. 10.4155/tde.15.30 26228775

[B210] McGowan J. W. Shao Q. Vig P. J. Bidwell G. L. III (2016). Intranasal administration of elastin-like polypeptide for therapeutic delivery to the central nervous system. *Drug Design Dev. Ther.* 10 2803–2813. 10.2147/dddt.s106216 27660412 PMC5019317

[B211] McKinney B. C. Sze W. Lee B. Murphy G. G. (2009). Impaired long-term potentiation and enhanced neuronal excitability in the amygdala of Ca(V)1.3 knockout mice. *Neurobiol. Learn. Mem.* 92 519–528. 10.1016/j.nlm.2009.06.012 19595780 PMC2747027

[B212] Meola G. Cardani R. (2015). Myotonic dystrophies: an update on clinical aspects, genetic, pathology, and molecular pathomechanisms. *Biochim. Biophys. Acta* 1852 594–606. 10.1016/j.bbadis.2014.05.019 24882752

[B213] Meola G. Sansone V. (2007). Cerebral involvement in myotonic dystrophies. *Muscle Nerve* 36 294–306. 10.1002/mus.20800 17486579

[B214] Meola G. Sansone V. Perani D. Scarone S. Cappa S. Dragoni C. (2003). Executive dysfunction and avoidant personality trait in myotonic dystrophy type 1 (DM-1) and in proximal myotonic myopathy (PROMM/DM-2). *Neuromusc. Disord.* 13 813–821. 10.1016/s0960-8966(03)00137-8 14678804

[B215] Miller J. N. Kruger A. Moser D. J. Gutmann L. van der Plas E. Koscik T. R. (2021). Cognitive deficits, apathy, and hypersomnolence represent the core brain symptoms of adult-onset myotonic dystrophy type 1. *Front. Neurol.* 12:700796. 10.3389/fneur.2021.700796 34276551 PMC8280288

[B216] Miller J. W. Urbinati C. R. Teng-Umnuay P. Stenberg M. G. Byrne B. J. Thornton C. A. (2000). Recruitment of human muscleblind proteins to (CUG)(n) expansions associated with myotonic dystrophy. *Embo J.* 19 4439–4448. 10.1093/emboj/19.17.4439 10970838 PMC302046

[B217] Miller R. G. Mitchell J. D. Moore D. H. (2012). Riluzole for amyotrophic lateral sclerosis (ALS)/motor neuron disease (MND). *Cochrane Database Syst. Rev.* 2012:Cd001447. 10.1002/14651858.CD001447.pub3 11687111

[B218] Miller T. M. Pestronk A. David W. Rothstein J. Simpson E. Appel S. H. (2013). An antisense oligonucleotide against SOD1 delivered intrathecally for patients with SOD1 familial amyotrophic lateral sclerosis: a phase 1, randomised, first-in-man study. *Lancet Neurol.* 12 435–442. 10.1016/s1474-4422(13)70061-9 23541756 PMC3712285

[B219] Minier L. Lignier B. Bouvet C. Gallais B. Camart N. (2018). A review of psychopathology features, personality, and coping in myotonic dystrophy type 1. *J. Neuromusc. Dis.* 5 279–294. 10.3233/jnd-180310 30040740 PMC6087440

[B220] Minnerop M. Gliem C. Kornblum C. (2018). Current progress in CNS imaging of myotonic dystrophy. *Front. Neurol.* 9:646. 10.3389/fneur.2018.00646 30186217 PMC6110944

[B221] Minnerop M. Weber B. Schoene-Bake J. C. Roeske S. Mirbach S. Anspach C. (2011). The brain in myotonic dystrophy 1 and 2: evidence for a predominant white matter disease. *Brain* 134(Pt 12), 3530–3546. 10.1093/brain/awr299 22131273 PMC3235566

[B222] Misra C. Bangru S. Lin F. Lam K. Koenig S. N. Lubbers E. R. (2020). Aberrant expression of a non-muscle RBFOX2 isoform triggers cardiac conduction defects in myotonic dystrophy. *Dev. Cell* 52 748.e6–763.e6. 10.1016/j.devcel.2020.01.037 32109384 PMC7098852

[B223] Mizukami K. Sasaki M. Baba A. Suzuki T. Shiraishi H. (1999). An autopsy case of myotonic dystrophy with mental disorders and various neuropathologic features. *Psychiatry Clin. Neurosci.* 53 51–55. 10.1046/j.1440-1819.1999.00470.x 10201284

[B224] Mizuno Y. Maeda N. Hamasaki H. Arahata H. Sasagasako N. Honda H. (2018). Four-repeat tau dominant pathology in a congenital myotonic dystrophy type 1 patient with mental retardation. *Brain Pathol.* 28 431–433. 10.1111/bpa.12603 29740938 PMC8028262

[B225] Modoni A. Silvestri G. Vita M. G. Quaranta D. Tonali P. A. Marra C. (2008). Cognitive impairment in myotonic dystrophy type 1 (DM1): a longitudinal follow-up study. *J. Neurol.* 255 1737–1742. 10.1007/s00415-008-0017-5 18821050

[B226] Mohan A. Goodwin M. Swanson M. S. (2014). RNA-protein interactions in unstable microsatellite diseases. *Brain Res.* 1584 3–14. 10.1016/j.brainres.2014.03.039 24709120 PMC4174988

[B227] Mondragon-Gonzalez R. Perlingeiro R. C. R. (2018). Recapitulating muscle disease phenotypes with myotonic dystrophy 1 induced pluripotent stem cells: a tool for disease modeling and drug discovery. *Dis. Models Mech.* 11:dmm034728. 10.1242/dmm.034728 29898953 PMC6078411

[B228] Morales F. Vásquez M. Corrales E. Vindas-Smith R. Santamaría-Ulloa C. Zhang B. (2020). Longitudinal increases in somatic mosaicism of the expanded CTG repeat in myotonic dystrophy type 1 are associated with variation in age-at-onset. *Hum. Mol. Genet.* 29 2496–2507. 10.1093/hmg/ddaa123 32601694

[B229] Morgenthaler T. I. Kapur V. K. Brown T. Swick T. J. Alessi C. Aurora R. N. (2007). Practice parameters for the treatment of narcolepsy and other hypersomnias of central origin. *Sleep* 30 1705–1711. 10.1093/sleep/30.12.1705 18246980 PMC2276123

[B230] Moxley R. T. III Griggs R. C. Goldblatt D. VanGelder V. Herr B. E. Thiel R. (1978). Decreased insulin sensitivity of forearm muscle in myotonic dystrophy. *J. Clin. Investig.* 62 857–867. 10.1172/jci109198 701484 PMC371838

[B231] Mulders S. A. van den Broek W. J. Wheeler T. M. Croes H. J. van Kuik-Romeijn P. de Kimpe S. J. (2009). Triplet-repeat oligonucleotide-mediated reversal of RNA toxicity in myotonic dystrophy. *Proc. Natl. Acad. Sci. U.S.A.* 106 13915–13920. 10.1073/pnas.0905780106 19667189 PMC2728995

[B232] Mulders S. A. van Engelen B. G. Wieringa B. Wansink D. G. (2010). Molecular therapy in myotonic dystrophy: focus on RNA gain-of-function. *Hum. Mol. Genet.* 19 R90–R97. 10.1093/hmg/ddq161 20406734

[B233] Muntoni F. Wood M. J. (2011). Targeting RNA to treat neuromuscular disease. *Nat. Rev. Drug Discov.* 10 621–637. 10.1038/nrd3459 21804598

[B234] Murlidharan G. Sakamoto K. Rao L. Corriher T. Wang D. Gao G. (2016). CNS-restricted transduction and CRISPR/Cas9-mediated gene deletion with an engineered AAV vector. *Mol. Ther. Nucleic Acids* 5:e338. 10.1038/mtna.2016.49 27434683 PMC5330941

[B235] Nakamori M. Gourdon G. Thornton C. A. (2011). Stabilization of expanded (CTG)•(CAG) repeats by antisense oligonucleotides. *Mol. Ther.* 19 2222–2227. 10.1038/mt.2011.191 21971425 PMC3242663

[B236] Nakamori M. Takahashi T. Yamazaki Y. Kurashige T. Yamawaki T. Matsumoto M. (2012). Cyclin-dependent kinase 5 immunoreactivity for granulovacuolar degeneration. *Neuroreport* 23 867–872. 10.1097/WNR.0b013e328358720b 22968343

[B237] Nakamori M. Taylor K. Mochizuki H. Sobczak K. Takahashi M. P. (2016). Oral administration of erythromycin decreases RNA toxicity in myotonic dystrophy. *Ann. Clin. Transl. Neurol.* 3 42–54. 10.1002/acn3.271 26783549 PMC4704483

[B238] Nakatani K. Matsumoto J. Nakamori M. Okamoto T. Murata A. Dohno C. (2020). The dimeric form of 1,3-diaminoisoquinoline derivative rescued the mis-splicing of Atp2a1 and Clcn1 genes in myotonic dystrophy type 1 mouse model. *Chemistry* 26 14305–14309. 10.1002/chem.202001572 32449537 PMC7702137

[B239] Nehra M. Uthappa U. T. Kumar V. Kumar R. Dixit C. Dilbaghi N. (2021). Nanobiotechnology-assisted therapies to manage brain cancer in personalized manner. *J. Control. Release* 338 224–243. 10.1016/j.jconrel.2021.08.027 34418523

[B240] Nelson C. E. Robinson-Hamm J. N. Gersbach C. A. (2017). Genome engineering: a new approach to gene therapy for neuromuscular disorders. *Nat. Rev. Neurol.* 13 647–661. 10.1038/nrneurol.2017.126 28960187

[B241] Nguyen Q. Yokota T. (2020). Degradation of toxic RNA in myotonic dystrophy using gapmer antisense oligonucleotides. *Methods Mol. Biol.* 2176 99–109. 10.1007/978-1-0716-0771-8_7 32865785

[B242] Nieuwenhuis S. Okkersen K. Widomska J. Blom P. t Hoen P. A. C. van Engelen B. (2019). Insulin signaling as a key moderator in myotonic dystrophy type 1. *Front. Neurol.* 10:1229. 10.3389/fneur.2019.01229 31849810 PMC6901991

[B243] Nishi M. Kimura T. Igeta M. Furuta M. Suenaga K. Matsumura T. (2020). Differences in splicing defects between the grey and white matter in myotonic dystrophy type 1 patients. *PLoS One* 15:e0224912. 10.1371/journal.pone.0224912 32407311 PMC7224547

[B244] Nutter C. A. Bubenik J. L. Oliveira R. Ivankovic F. Sznajder ŁJ. Kidd B. M. (2019). Cell-type-specific dysregulation of RNA alternative splicing in short tandem repeat mouse knockin models of myotonic dystrophy. *Genes Dev.* 33 1635–1640. 10.1101/gad.328963.119 31624084 PMC6942047

[B245] Oana K. Oma Y. Suo S. Takahashi M. P. Nishino I. Takeda S. (2013). Manumycin A corrects aberrant splicing of Clcn1 in myotonic dystrophy type 1 (DM1) mice. *Sci. Rep.* 3:2142. 10.1038/srep02142 23828222 PMC3701899

[B246] Ofori L. O. Hoskins J. Nakamori M. Thornton C. A. Miller B. L. (2012). From dynamic combinatorial ‘hit’ to lead: in vitro and in vivo activity of compounds targeting the pathogenic RNAs that cause myotonic dystrophy. *Nucleic Acids Res.* 40 6380–6390. 10.1093/nar/gks298 22492623 PMC3401475

[B247] Ogata A. Terae S. Fujita M. Tashiro K. (1998). Anterior temporal white matter lesions in myotonic dystrophy with intellectual impairment: an MRI and neuropathological study. *Neuroradiology* 40 411–415. 10.1007/s002340050613 9730337

[B248] Okkersen K. Buskes M. Groenewoud J. Kessels R. P. C. Knoop H. van Engelen B. (2017a). The cognitive profile of myotonic dystrophy type 1: a systematic review and meta-analysis. *Cortex* 95 143–155. 10.1016/j.cortex.2017.08.008 28892766

[B249] Okkersen K. Monckton D. G. Le N. Tuladhar A. M. Raaphorst J. van Engelen B. G. M. (2017b). Brain imaging in myotonic dystrophy type 1: a systematic review. *Neurology* 89 960–969. 10.1212/wnl.0000000000004300 28768849

[B250] Okkersen K. Jimenez-Moreno C. Wenninger S. Daidj F. Glennon J. Cumming S. (2018). Cognitive behavioural therapy with optional graded exercise therapy in patients with severe fatigue with myotonic dystrophy type 1: a multicentre, single-blind, randomised trial. *Lancet Neurol.* 17 671–680. 10.1016/s1474-4422(18)30203-5 29934199

[B251] Ono S. Kanda F. Takahashi K. Fukuoka Y. Jinnai K. Kurisaki H. (1995). Neuronal cell loss in the dorsal raphe nucleus and the superior central nucleus in myotonic dystrophy: a clinicopathological correlation. *Acta Neuropathol.* 89 122–125. 10.1007/bf00296355 7732784

[B252] Ono S. Kanda F. Takahashi K. Fukuoka Y. Jinnai K. Kurisaki H. (1996). Neuronal loss in the medullary reticular formation in myotonic dystrophy: a clinicopathological study. *Neurology* 46 228–231. 10.1212/wnl.46.1.228 8559381

[B253] Ono S. Takahashi K. Jinnai K. Kanda F. Fukuoka Y. Kurisaki H. (1998). Loss of serotonin-containing neurons in the raphe of patients with myotonic dystrophy: a quantitative immunohistochemical study and relation to hypersomnia. *Neurology* 50 535–538. 10.1212/wnl.50.2.535 9484393

[B254] Otero B. A. Poukalov K. Hildebrandt R. P. Thornton C. A. Jinnai K. Fujimura H. (2021). Transcriptome alterations in myotonic dystrophy frontal cortex. *Cell Rep.* 34:108634. 10.1016/j.celrep.2020.108634 33472074 PMC9272850

[B255] Oude Ophuis R. J. Mulders S. A. van Herpen R. E. van de Vorstenbosch R. Wieringa B. Wansink D. G. (2009). DMPK protein isoforms are differentially expressed in myogenic and neural cell lineages. *Muscle Nerve* 40 545–555. 10.1002/mus.21352 19626675

[B256] Pandey S. K. Wheeler T. M. Justice S. L. Kim A. Younis H. S. Gattis D. (2015). Identification and characterization of modified antisense oligonucleotides targeting DMPK in mice and nonhuman primates for the treatment of myotonic dystrophy type 1. *J. Pharmacol. Exp. Ther.* 355 329–340. 10.1124/jpet.115.226969 26330536 PMC4613955

[B257] Pascual-Gilabert M. López-Castel A. Artero R. (2021). Myotonic dystrophy type 1 drug development: a pipeline toward the market. *Drug Discov. Today* 26 1765–1772. 10.1016/j.drudis.2021.03.024 33798646 PMC8372527

[B258] Pegoraro V. Cudia P. Baba A. Angelini C. (2020). MyomiRNAs and myostatin as physical rehabilitation biomarkers for myotonic dystrophy. *Neurol. Sci.* 41 2953–2960. 10.1007/s10072-020-04409-2 32350671

[B259] Perbellini R. Greco S. Sarra-Ferraris G. Cardani R. Capogrossi M. C. Meola G. (2011). Dysregulation and cellular mislocalization of specific miRNAs in myotonic dystrophy type 1. *Neuromusc. Disord.* 21 81–88. 10.1016/j.nmd.2010.11.012 21169019

[B260] Perfetti A. Greco S. Bugiardini E. Cardani R. Gaia P. Gaetano C. (2014). Plasma microRNAs as biomarkers for myotonic dystrophy type 1. *Neuromusc. Disord.* 24 509–515. 10.1016/j.nmd.2014.02.005 24679513

[B261] Perfetti A. Greco S. Cardani R. Fossati B. Cuomo G. Valaperta R. (2016). Validation of plasma microRNAs as biomarkers for myotonic dystrophy type 1. *Sci. Rep.* 6:38174. 10.1038/srep38174 27905532 PMC5131283

[B262] Peric S. Brajkovic L. Belanovic B. Ilic V. Salak-Djokic B. Basta I. (2017a). Brain positron emission tomography in patients with myotonic dystrophy type 1 and type 2. *J. Neurol. Sci.* 378 187–192. 10.1016/j.jns.2017.05.013 28566162

[B263] Peric S. Heatwole C. Durovic E. Kacar A. Nikolic A. Basta I. (2017b). Prospective measurement of quality of life in myotonic dystrophy type 1. *Acta Neurol. Scand.* 136 694–697. 10.1111/ane.12788 28660733

[B264] Peric S. Pavlovic A. Ralic V. Dobricic V. Basta I. Lavrnic D. (2014b). Transcranial sonography in patients with myotonic dystrophy type 1. *Muscle Nerve* 50 278–282. 10.1002/mus.24162 24395217

[B265] Peric S. Mandic-Stojmenovic G. Markovic I. Stefanova E. Ilic V. Parojcic A. (2014a). Cerebrospinal fluid biomarkers of neurodegeneration in patients with juvenile and classic myotonic dystrophy type 1. *Eur. J. Neurol.* 21 231–237. 10.1111/ene.12237 23834502

[B266] Peric S. Vujnic M. Dobricic V. Marjanovic A. Basta I. Novakovic I. (2016). Five-year study of quality of life in myotonic dystrophy. *Acta Neurol. Scand.* 134 346–351. 10.1111/ane.12549 27696366

[B267] Pettersson O. J. Aagaard L. Jensen T. G. Damgaard C. K. (2015). Molecular mechanisms in DM1 - a focus on foci. *Nucleic Acids Res.* 43 2433–2441. 10.1093/nar/gkv029 25605794 PMC4344492

[B268] Pincherle A. Patruno V. Raimondi P. Moretti S. Dominese A. Martinelli-Boneschi F. (2012). Sleep breathing disorders in 40 Italian patients with Myotonic dystrophy type 1. *Neuromusc. Disord.* 22 219–224. 10.1016/j.nmd.2011.08.010 22137426

[B269] Pinto B. S. Saxena T. Oliveira R. Méndez-Gómez H. R. Cleary J. D. Denes L. T. (2017). Impeding transcription of expanded microsatellite repeats by deactivated Cas9. *Mol. Cell* 68 479.e5–490.e5. 10.1016/j.molcel.2017.09.033 29056323 PMC6013302

[B270] Quera Salva M. A. Blumen M. Jacquette A. Durand M. C. Andre S. De Villiers M. (2006). Sleep disorders in childhood-onset myotonic dystrophy type 1. *Neuromusc. Disord.* 16 564–570. 10.1016/j.nmd.2006.06.007 16934465

[B271] Raaijmakers R. H. L. Ripken L. Ausems C. R. M. Wansink D. G. (2019). CRISPR/Cas applications in myotonic dystrophy: expanding opportunities. *Int. J. Mol. Sci.* 20:3689. 10.3390/ijms20153689 31357652 PMC6696057

[B272] Rakocevic-Stojanovic V. Peric S. Madzarevic R. Dobricic V. Ralic V. Ilic V. (2014). Significant impact of behavioral and cognitive impairment on quality of life in patients with myotonic dystrophy type 1. *Clin. Neurol. Neurosurg.* 126 76–81. 10.1016/j.clineuro.2014.08.021 25215445

[B273] Ramon-Duaso C. Gener T. Consegal M. Fernández-Avilés C. Gallego J. J. Castarlenas L. (2019). Methylphenidate attenuates the cognitive and mood alterations observed in Mbnl2 knockout mice and reduces microglia overexpression. *Cerebr. Cortex* 29 2978–2997. 10.1093/cercor/bhy164 30060068 PMC7963113

[B274] Ramon-Duaso C. Rodríguez-Morató J. Selma-Soriano E. Fernández-Avilés C. Artero R. de la Torre R. (2020). Protective effects of mirtazapine in mice lacking the Mbnl2 gene in forebrain glutamatergic neurons: relevance for myotonic dystrophy 1. *Neuropharmacology* 170:108030. 10.1016/j.neuropharm.2020.108030 32171677

[B275] Rau F. Freyermuth F. Fugier C. Villemin J. P. Fischer M. C. Jost B. (2011). Misregulation of miR-1 processing is associated with heart defects in myotonic dystrophy. *Nat. Struct. Mol. Biol.* 18 840–845. 10.1038/nsmb.2067 21685920

[B276] Reddy K. Jenquin J. R. Cleary J. D. Berglund J. A. (2019a). Mitigating RNA toxicity in myotonic dystrophy using small molecules. *Int. J. Mol. Sci.* 20:4017. 10.3390/ijms20164017 31426500 PMC6720693

[B277] Reddy K. Jenquin J. R. McConnell O. L. Cleary J. D. Richardson J. I. Pinto B. S. (2019b). A CTG repeat-selective chemical screen identifies microtubule inhibitors as selective modulators of toxic CUG RNA levels. *Proc. Natl. Acad. Sci. U.S.A.* 116 20991–21000. 10.1073/pnas.1901893116 31570586 PMC6800345

[B278] Reddy S. Smith D. B. Rich M. M. Leferovich J. M. Reilly P. Davis B. M. (1996). Mice lacking the myotonic dystrophy protein kinase develop a late onset progressive myopathy. *Nat. Genet.* 13 325–335. 10.1038/ng0796-325 8673132

[B279] Renard D. Collombier L. Castelli C. Pouget J. P. Kotzki P. O. Boudousq V. (2016). In myotonic dystrophy type 1 reduced FDG-uptake on FDG-PET is most severe in Brodmann area 8. *BMC Neurol.* 16:100. 10.1186/s12883-016-0630-3 27411408 PMC4944494

[B280] Richard G. F. (2015). Shortening trinucleotide repeats using highly specific endonucleases: a possible approach to gene therapy? *Trends Genet.* 31 177–186. 10.1016/j.tig.2015.02.003 25743488

[B281] Richard G. F. Viterbo D. Khanna V. Mosbach V. Castelain L. Dujon B. (2014). Highly specific contractions of a single CAG/CTG trinucleotide repeat by TALEN in yeast. *PLoS One* 9:e95611. 10.1371/journal.pone.0095611 24748175 PMC3991675

[B282] Romeo V. Pegoraro E. Squarzanti F. Sorarù G. Ferrati C. Ermani M. (2010b). Retrospective study on PET-SPECT imaging in a large cohort of myotonic dystrophy type 1 patients. *Neurol. Sci.* 31 757–763. 10.1007/s10072-010-0406-2 20842397

[B283] Romeo V. Pegoraro E. Ferrati C. Squarzanti F. Sorarù G. Palmieri A. (2010a). Brain involvement in myotonic dystrophies: neuroimaging and neuropsychological comparative study in DM1 and DM2. *J. Neurol.* 257 1246–1255. 10.1007/s00415-010-5498-3 20221771

[B284] Rosahl T. W. Geppert M. Spillane D. Herz J. Hammer R. E. Malenka R. C. (1993). Short-term synaptic plasticity is altered in mice lacking synapsin I. *Cell* 75 661–670. 10.1016/0092-8674(93)90487-b 7902212

[B285] Rosman N. P. Kakulas B. A. (1966). Mental deficiency associated with muscular dystrophy. A neuropathological study. *Brain* 89 769–788. 10.1093/brain/89.4.769 4163581

[B286] Sabater-Arcis M. Bargiela A. Furling D. Artero R. (2020). miR-7 restores phenotypes in myotonic dystrophy muscle cells by repressing hyperactivated autophagy. *Mol. Ther. Nucleic Acids* 19 278–292. 10.1016/j.omtn.2019.11.012 31855836 PMC6926285

[B287] Sakurai T. (2014). The role of orexin in motivated behaviours. *Nat. Rev. Neurosci.* 15 719–731. 10.1038/nrn3837 25301357

[B288] Salvatori S. Furlan S. Fanin M. Picard A. Pastorello E. Romeo V. (2009). Comparative transcriptional and biochemical studies in muscle of myotonic dystrophies (DM1 and DM2). *Neurol. Sci.* 30 185–192. 10.1007/s10072-009-0048-4 19326042

[B289] Sansone V. Gandossini S. Cotelli M. Calabria M. Zanetti O. Meola G. (2007). Cognitive impairment in adult myotonic dystrophies: a longitudinal study. *Neurol. Sci.* 28 9–15. 10.1007/s10072-007-0742-z 17385090

[B290] Schmitz F. Pierozan P. Rodrigues A. F. Biasibetti H. Coelho D. M. Mussulini B. H. (2016). Chronic treatment with a clinically relevant dose of methylphenidate increases glutamate levels in cerebrospinal fluid and impairs glutamatergic homeostasis in prefrontal cortex of juvenile rats. *Mol. Neurobiol.* 53 2384–2396. 10.1007/s12035-015-9219-x 26001762

[B291] Schmitz F. Pierozan P. Rodrigues A. F. Biasibetti H. Grings M. Zanotto B. (2017). Methylphenidate decreases ATP levels and impairs glutamate uptake and Na(+),K(+)-ATPase activity in juvenile rat hippocampus. *Mol. Neurobiol.* 54 7796–7807. 10.1007/s12035-016-0289-1 27844288

[B292] Schneider-Gold C. Bellenberg B. Prehn C. Krogias C. Schneider R. Klein J. (2015). Cortical and subcortical grey and white matter atrophy in myotonic dystrophies type 1 and 2 is associated with cognitive impairment, depression and daytime sleepiness. *PLoS One* 10:e0130352. 10.1371/journal.pone.0130352 26114298 PMC4482602

[B293] Schoch K. M. Miller T. M. (2017). Antisense oligonucleotides: translation from mouse models to human neurodegenerative diseases. *Neuron* 94 1056–1070. 10.1016/j.neuron.2017.04.010 28641106 PMC5821515

[B294] Seijger C. Raaphorst J. Vonk J. van Engelen B. Heijerman H. Stigter N. (2021). New insights in adherence and survival in myotonic dystrophy patients using home mechanical ventilation. *Respir. Int. Rev. Thor. Dis.* 100 154–163. 10.1159/000511962 33461194 PMC7949200

[B295] Sellier C. Cerro-Herreros E. Blatter M. Freyermuth F. Gaucherot A. Ruffenach F. (2018). rbFOX1/MBNL1 competition for CCUG RNA repeats binding contributes to myotonic dystrophy type 1/type 2 differences. *Nat. Commun.* 9:2009. 10.1038/s41467-018-04370-x 29789616 PMC5964235

[B296] Sergeant N. Sablonnière B. Schraen-Maschke S. Ghestem A. Maurage C. A. Wattez A. (2001). Dysregulation of human brain microtubule-associated tau mRNA maturation in myotonic dystrophy type 1. *Hum. Mol. Genet.* 10 2143–2155. 10.1093/hmg/10.19.2143 11590131

[B297] Serra L. Scocchia M. Meola G. D’Amelio M. Bruschini M. Silvestri G. (2020b). Ventral tegmental area dysfunction affects decision-making in patients with myotonic dystrophy type-1. *Cortex* 128 192–202. 10.1016/j.cortex.2020.03.022 32361267

[B298] Serra L. Bianchi G. Bruschini M. Giulietti G. Domenico C. D. Bonarota S. (2020a). Abnormal cortical thickness is associated with deficits in social cognition in patients with myotonic dystrophy type 1. *Front. Neurol.* 11:113. 10.3389/fneur.2020.00113 32180756 PMC7059122

[B299] Serra L. Cercignani M. Bruschini M. Cipolotti L. Mancini M. Silvestri G. (2016a). “I know that you know that i know”: neural substrates associated with social cognition deficits in DM1 patients. *PLoS One* 11:e0156901. 10.1371/journal.pone.0156901 27258100 PMC4892543

[B300] Serra L. Mancini M. Silvestri G. Petrucci A. Masciullo M. Spanò B. (2016b). Brain connectomics’ modification to clarify motor and nonmotor features of myotonic dystrophy type 1. *Neural Plast.* 2016:2696085. 10.1155/2016/2696085 27313901 PMC4897716

[B301] Serra L. Petrucci A. Spanò B. Torso M. Olivito G. Lispi L. (2015). How genetics affects the brain to produce higher-level dysfunctions in myotonic dystrophy type 1. *Funct. Neurol.* 30 21–31. 26214024 PMC4520669

[B302] Serra L. Silvestri G. Petrucci A. Basile B. Masciullo M. Makovac E. (2014). Abnormal functional brain connectivity and personality traits in myotonic dystrophy type 1. *JAMA Neurol.* 71 603–611. 10.1001/jamaneurol.2014.130 24664202

[B303] Shimizu E. Tang Y. P. Rampon C. Tsien J. Z. (2000). NMDA receptor-dependent synaptic reinforcement as a crucial process for memory consolidation. *Science* 290 1170–1174. 10.1126/science.290.5494.1170 11073458

[B304] Siboni R. B. Nakamori M. Wagner S. D. Struck A. J. Coonrod L. A. Harriott S. A. (2015b). Actinomycin D specifically reduces expanded CUG repeat RNA in myotonic dystrophy models. *Cell Rep.* 13 2386–2394. 10.1016/j.celrep.2015.11.028 26686629 PMC4691565

[B305] Siboni R. B. Bodner M. J. Khalifa M. M. Docter A. G. Choi J. Y. Nakamori M. (2015a). Biological efficacy and toxicity of diamidines in myotonic dystrophy type 1 models. *J. Med. Chem.* 58 5770–5780. 10.1021/acs.jmedchem.5b00356 26103061 PMC4972181

[B306] Sicot G. Servais L. Dinca D. M. Leroy A. Prigogine C. Medja F. (2017). Downregulation of the glial GLT1 glutamate transporter and purkinje cell dysfunction in a mouse model of myotonic dystrophy. *Cell Rep.* 19 2718–2729. 10.1016/j.celrep.2017.06.006 28658620 PMC8496958

[B307] Simoncini C. Spadoni G. Lai E. Santoni L. Angelini C. Ricci G. (2020). Central nervous system involvement as outcome measure for clinical trials efficacy in myotonic dystrophy type 1. *Front. Neurol.* 11:624. 10.3389/fneur.2020.00624 33117249 PMC7575726

[B308] Singh R. K. Xia Z. Bland C. S. Kalsotra A. Scavuzzo M. A. Curk T. (2014). Rbfox2-coordinated alternative splicing of Mef2d and Rock2 controls myoblast fusion during myogenesis. *Mol. Cell* 55 592–603. 10.1016/j.molcel.2014.06.035 25087874 PMC4142074

[B309] Sistiaga A. Urreta I. Jodar M. Cobo A. M. Emparanza J. Otaegui D. (2010). Cognitive/personality pattern and triplet expansion size in adult myotonic dystrophy type 1 (DM1): CTG repeats, cognition and personality in DM1. *Psychol. Med.* 40 487–495. 10.1017/s0033291709990602 19627641

[B310] Smith R. A. Miller T. M. Yamanaka K. Monia B. P. Condon T. P. Hung G. (2006). Antisense oligonucleotide therapy for neurodegenerative disease. *J. Clin. Investig.* 116 2290–2296. 10.1172/jci25424 16878173 PMC1518790

[B311] Sobczak K. Wheeler T. M. Wang W. Thornton C. A. (2013). RNA interference targeting CUG repeats in a mouse model of myotonic dystrophy. *Mol. Ther.* 21 380–387. 10.1038/mt.2012.222 23183533 PMC3594017

[B312] Solovyeva E. M. Ibebunjo C. Utzinger S. Eash J. K. Dunbar A. Naumann U. (2021). New insights into molecular changes in skeletal muscle aging and disease: differential alternative splicing and senescence. *Mech. Age. Dev.* 197:111510. 10.1016/j.mad.2021.111510 34019916

[B313] Song J. Lu C. Leszek J. Zhang J. (2021). Design and development of nanomaterial-based drug carriers to overcome the blood-brain barrier by using different transport mechanisms. *Int. J. Mol. Sci.* 22:10118. 10.3390/ijms221810118 34576281 PMC8465340

[B314] Song K. Y. Guo X. M. Wang H. Q. Zhang L. Huang S. Y. Huo Y. C. (2020). MBNL1 reverses the proliferation defect of skeletal muscle satellite cells in myotonic dystrophy type 1 by inhibiting autophagy via the mTOR pathway. *Cell Death Dis.* 11:545. 10.1038/s41419-020-02756-8 32683410 PMC7368861

[B315] Stepniak-Konieczna E. Konieczny P. Cywoniuk P. Dluzewska J. Sobczak K. (2020). AON-induced splice-switching and DMPK pre-mRNA degradation as potential therapeutic approaches for myotonic dystrophy type 1. *Nucleic Acids Research* 48 2531–2543. 10.1093/nar/gkaa007 31965181 PMC7049696

[B316] Steyaert J. Umans S. Willekens D. Legius E. Pijkels E. de Die-Smulders C. (1997). A study of the cognitive and psychological profile in 16 children with congenital or juvenile myotonic dystrophy. *Clin. Genet.* 52 135–141. 10.1111/j.1399-0004.1997.tb02533.x 9377801

[B317] Strutt S. C. Torrez R. M. Kaya E. Negrete O. A. Doudna J. A. (2018). RNA-dependent RNA targeting by CRISPR-Cas9. *eLife* 7:e32724. 10.7554/eLife.32724 29303478 PMC5796797

[B318] Subramony S. H. Wymer J. P. Pinto B. S. Wang E. T. (2020). Sleep disorders in myotonic dystrophies. *Muscle Nerve* 62 309–320. 10.1002/mus.26866 32212331

[B319] Sudhof T. C. (2004). The synaptic vesicle cycle. *Annu. Rev. Neurosci.* 27 509–547. 10.1146/annurev.neuro.26.041002.131412 15217342

[B320] Suenaga K. Lee K. Y. Nakamori M. Tatsumi Y. Takahashi M. P. Fujimura H. (2012). Muscleblind-like 1 knockout mice reveal novel splicing defects in the myotonic dystrophy brain. *PLoS One* 7:e33218. 10.1371/journal.pone.0033218 22427994 PMC3302840

[B321] Sugiyama A. Sone D. Sato N. Kimura Y. Ota M. Maikusa N. (2017). Brain gray matter structural network in myotonic dystrophy type 1. *PLoS One* 12:e0187343. 10.1371/journal.pone.0187343 29095898 PMC5667809

[B322] Summerton J. (1999). Morpholino antisense oligomers: the case for an RNase H-independent structural type. *Biochim. Biophys. Acta* 1489 141–158. 10.1016/s0167-4781(99)00150-5 10807004

[B323] Sun N. Zhao H. (2013). Transcription activator-like effector nucleases (TALENs): a highly efficient and versatile tool for genome editing. *Biotechnol. Bioeng.* 110 1811–1821. 10.1002/bit.24890 23508559

[B324] Suzuki K. Miyamoto M. Miyamoto T. Matsubara T. Inoue Y. Iijima M. (2018). Cerebrospinal fluid orexin-A levels in systemic lupus erythematosus patients presenting with excessive daytime sleepiness. *Lupus* 27 1847–1853. 10.1177/0961203318778767 29848165

[B325] Suzuki Y. Holmes J. B. Cerritelli S. M. Sakhuja K. Minczuk M. Holt I. J. (2010). An upstream open reading frame and the context of the two AUG codons affect the abundance of mitochondrial and nuclear RNase H1. *Mol. Cell. Biol.* 30 5123–5134. 10.1128/mcb.00619-10 20823270 PMC2953059

[B326] Symonds T. Randall J. A. Campbell P. (2017). Review of patient-reported outcome measures for use in myotonic dystrophy type 1 patients. *Muscle Nerve* 56 86–92. 10.1002/mus.25469 27862031

[B327] Takado Y. Terajima K. Ohkubo M. Okamoto K. Shimohata T. Nishizawa M. (2015). Diffuse brain abnormalities in myotonic dystrophy type 1 detected by 3.0 T proton magnetic resonance spectroscopy. *Eur. Neurol.* 73 247–256. 10.1159/000371575 25824277

[B328] Takeda A. Kobayakawa M. Suzuki A. Tsuruya N. Kawamura M. (2009). Lowered sensitivity to facial emotions in myotonic dystrophy type 1. *J. Neurol. Sci.* 280 35–39. 10.1016/j.jns.2009.01.014 19223261

[B329] Timchenko L. T. Miller J. W. Timchenko N. A. DeVore D. R. Datar K. V. Lin L. (1996). Identification of a (CUG)n triplet repeat RNA-binding protein and its expression in myotonic dystrophy. *Nucleic Acids Res.* 24 4407–4414. 10.1093/nar/24.22.4407 8948631 PMC146274

[B330] Todd P. K. Ackall F. Y. Hur J. Sharma K. Paulson H. L. Dowling J. J. (2014). Transcriptional changes and developmental abnormalities in a zebrafish model of myotonic dystrophy type 1. *Dis. Models Mech.* 7 143–155. 10.1242/dmm.012427 24092878 PMC3882056

[B331] Tomé S. Holt I. Edelmann W. Morris G. E. Munnich A. Pearson C. E. (2009). MSH2 ATPase domain mutation affects CTG^∗^CAG repeat instability in transgenic mice. *PLoS Genet.* 5:e1000482. 10.1371/journal.pgen.1000482 19436705 PMC2674216

[B332] Toth A. Lovadi E. Komoly S. Schwarcz A. Orsi G. Perlaki G. (2015). Cortical involvement during myotonia in myotonic dystrophy: an fMRI study. *Acta Neurol. Scand.* 132 65–72. 10.1111/ane.12360 25630356

[B333] Tremblay M. Muslemani S. Côté I. Gagnon C. Fortin J. Gallais B. (2021). Accomplishment of instrumental activities of daily living and its relationship with cognitive functions in adults with myotonic dystrophy type 1 childhood phenotype: an exploratory study. *BMC Psychol.* 9:56. 10.1186/s40359-021-00562-1 33865455 PMC8052658

[B334] Udd B. Krahe R. (2012). The myotonic dystrophies: molecular, clinical, and therapeutic challenges. *Lancet Neurol.* 11 891–905. 10.1016/s1474-4422(12)70204-1 22995693

[B335] van Agtmaal E. L. André L. M. Willemse M. Cumming S. A. van Kessel I. D. G. van den Broek W. (2017). CRISPR/Cas9-induced (CTG⋅CAG)(n) repeat instability in the myotonic dystrophy type 1 locus: implications for therapeutic genome editing. *Mol. Ther.* 25 24–43. 10.1016/j.ymthe.2016.10.014 28129118 PMC5363205

[B336] van de Vondervoort I. Amiri H. Bruchhage M. M. K. Oomen C. A. Rustogi N. Cooper J. D. (2019). Converging evidence points towards a role of insulin signaling in regulating compulsive behavior. *Transl. Psychiatry* 9:225. 10.1038/s41398-019-0559-6 31515486 PMC6742634

[B337] van de Vondervoort I. Poelmans G. Aschrafi A. Pauls D. L. Buitelaar J. K. Glennon J. C. (2016). An integrated molecular landscape implicates the regulation of dendritic spine formation through insulin-related signalling in obsessive-compulsive disorder. *J. Psychiatry Neurosci.* 41 280–285. 10.1503/jpn.140327 26854754 PMC4915937

[B338] van der Bent M. L. Paulino da Silva Filho O. van Luijk J. Brock R. Wansink D. G. (2018). Assisted delivery of antisense therapeutics in animal models of heritable neurodegenerative and neuromuscular disorders: a systematic review and meta-analysis. *Sci. Rep.* 8:4181. 10.1038/s41598-018-22316-7 29520012 PMC5843643

[B339] van der Plas E. Hamilton M. J. Miller J. N. Koscik T. R. Long J. D. Cumming S. (2019). Brain structural features of myotonic dystrophy type 1 and their relationship with CTG repeats. *J. Neuromuscul. Dis.* 6 321–332. 10.3233/jnd-190397 31306140 PMC7480174

[B340] van der Velden B. G. Okkersen K. Kessels R. P. Groenewoud J. van Engelen B. Knoop H. (2019). Affective symptoms and apathy in myotonic dystrophy type 1 a systematic review and meta-analysis. *J. Affect. Disord.* 250 260–269. 10.1016/j.jad.2019.03.036 30870776

[B341] van Engelen B. (2015). Cognitive behaviour therapy plus aerobic exercise training to increase activity in patients with myotonic dystrophy type 1 (DM1) compared to usual care (OPTIMISTIC): study protocol for randomised controlled trial. *Trials* 16:224. 10.1186/s13063-015-0737-7 26002596 PMC4449962

[B342] Vita G. Vita G. L. Musumeci O. Rodolico C. Messina S. (2019). Genetic neuromuscular disorders: living the era of a therapeutic revolution. Part 2: diseases of motor neuron and skeletal muscle. *Neurol. Sci.* 40 671–681. 10.1007/s10072-019-03764-z 30805745

[B343] Voellenkle C. Perfetti A. Carrara M. Fuschi P. Renna L. V. Longo M. (2019). Dysregulation of circular RNAs in myotonic dystrophy type 1. *Int. J. Mol. Sci.* 20:1938. 10.3390/ijms20081938 31010208 PMC6515344

[B344] Walder R. Y. Walder J. A. (1988). Role of RNase H in hybrid-arrested translation by antisense oligonucleotides. *Proc. Natl. Acad. Sci. U.S.A.* 85 5011–5015. 10.1073/pnas.85.14.5011 2839827 PMC281677

[B345] Walker G. L. Rosser R. Mastaglia F. L. Walton J. N. (1984). Psychometric and cranial CT study in myotonic dystrophy. *Clin. Exp. Neurol.* 20 161–167. 6568937

[B346] Wang E. T. Cody N. A. Jog S. Biancolella M. Wang T. T. Treacy D. J. (2012). Transcriptome-wide regulation of pre-mRNA splicing and mRNA localization by muscleblind proteins. *Cell* 150 710–724. 10.1016/j.cell.2012.06.041 22901804 PMC3428802

[B347] Wang E. T. Taliaferro J. M. Lee J. A. Sudhakaran I. P. Rossoll W. Gross C. (2016). Dysregulation of mRNA localization and translation in genetic disease. *J. Neurosci.* 36 11418–11426. 10.1523/jneurosci.2352-16.2016 27911744 PMC5125209

[B348] Wang G. S. Kuyumcu-Martinez M. N. Sarma S. Mathur N. Wehrens X. H. Cooper T. A. (2009). PKC inhibition ameliorates the cardiac phenotype in a mouse model of myotonic dystrophy type 1. *J. Clin. Investig.* 119 3797–3806. 10.1172/jci37976 19907076 PMC2786786

[B349] Wang M. Weng W. C. Stock L. Lindquist D. Martinez A. Gourdon G. (2019). Correction of glycogen synthase kinase 3β in myotonic dystrophy 1 reduces the mutant RNA and improves postnatal survival of DMSXL mice. *Mol. Cell. Biol.* 39:e00155–19. 10.1128/mcb.00155-19 31383751 PMC6791656

[B350] Wang P. Y. Chang K. T. Lin Y. M. Kuo T. Y. Wang G. S. (2018). Ubiquitination of MBNL1 Is required for its cytoplasmic localization and function in promoting neurite outgrowth. *Cell Rep.* 22 2294–2306. 10.1016/j.celrep.2018.02.025 29490267

[B351] Wang P. Y. Lin Y. M. Wang L. H. Kuo T. Y. Cheng S. J. Wang G. S. (2017). Reduced cytoplasmic MBNL1 is an early event in a brain-specific mouse model of myotonic dystrophy. *Hum. Mol. Genet.* 26 2247–2257. 10.1093/hmg/ddx115 28369378

[B352] Wang Y. Hao L. Wang H. Santostefano K. Thapa A. Cleary J. (2018). Therapeutic genome editing for myotonic dystrophy type 1 using CRISPR/Cas9. *Mol. Ther.* 26 2617–2630. 10.1016/j.ymthe.2018.09.003 30274788 PMC6225032

[B353] Wang Y. Miao L. Satterlee A. Huang L. (2015). Delivery of oligonucleotides with lipid nanoparticles. *Adv. Drug Deliv. Rev.* 87 68–80. 10.1016/j.addr.2015.02.007 25733311 PMC4539950

[B354] Wansink D. G. van Herpen R. E. Coerwinkel-Driessen M. M. Groenen P. J. Hemmings B. A. Wieringa B. (2003). Alternative splicing controls myotonic dystrophy protein kinase structure, enzymatic activity, and subcellular localization. *Mol. Cell. Biol.* 23 5489–5501. 10.1128/mcb.23.16.5489-5501.2003 12897125 PMC166319

[B355] Warf M. B. Nakamori M. Matthys C. M. Thornton C. A. Berglund J. A. (2009). Pentamidine reverses the splicing defects associated with myotonic dystrophy. *Proc. Natl. Acad. Sci. U.S.A.* 106 18551–18556. 10.1073/pnas.0903234106 19822739 PMC2774031

[B356] Weber Y. G. Roebling R. Kassubek J. Hoffmann S. Rosenbohm A. Wolf M. (2010). Comparative analysis of brain structure, metabolism, and cognition in myotonic dystrophy 1 and 2. *Neurology* 74 1108–1117. 10.1212/WNL.0b013e3181d8c35f 20220122

[B357] Wei C. Jones K. Timchenko N. A. Timchenko L. (2013). GSK3β is a new therapeutic target for myotonic dystrophy type 1. *Rare Dis.* 1:e26555. 10.4161/rdis.26555 25003008 PMC3927489

[B358] Weijs R. Okkersen K. van Engelen B. Küsters B. Lammens M. Aronica E. (2021). Human brain pathology in myotonic dystrophy type 1: a systematic review. *Neuropathology* 41 3–20. 10.1111/neup.12721 33599033 PMC7986875

[B359] Wheeler T. M. Leger A. J. Pandey S. K. MacLeod A. R. Nakamori M. Cheng S. H. (2012). Targeting nuclear RNA for in vivo correction of myotonic dystrophy. *Nature* 488 111–115. 10.1038/nature11362 22859208 PMC4221572

[B360] Wheeler T. M. Sobczak K. Lueck J. D. Osborne R. J. Lin X. Dirksen R. T. (2009). Reversal of RNA dominance by displacement of protein sequestered on triplet repeat RNA. *Science* 325 336–339. 10.1126/science.1173110 19608921 PMC4109973

[B361] Winblad S. Hellström P. Lindberg C. Hansen S. (2006). Facial emotion recognition in myotonic dystrophy type 1 correlates with CTG repeat expansion. *J. Neurol. Neurosurg. Psychiatry* 77 219–223. 10.1136/jnnp.2005.070763 16421126 PMC2077576

[B362] Winblad S. Månsson J. E. Blennow K. Jensen C. Samuelsson L. Lindberg C. (2008). Cerebrospinal fluid tau and amyloid beta42 protein in patients with myotonic dystrophy type 1. *Eur. J. Neurol.* 15 947–952. 10.1111/j.1468-1331.2008.02217.x 18637827

[B363] Winblad S. Samuelsson L. Lindberg C. Meola G. (2016). Cognition in myotonic dystrophy type 1: a 5-year follow-up study. *Eur. J. Neurol.* 23 1471–1476. 10.1111/ene.13062 27323306

[B364] Wojciechowska M. Taylor K. Sobczak K. Napierala M. Krzyzosiak W. J. (2014). Small molecule kinase inhibitors alleviate different molecular features of myotonic dystrophy type 1. *RNA Biol.* 11 742–754. 10.4161/rna.28799 24824895 PMC4156505

[B365] Woo J. Lee H. W. Park J. S. (2019). Differences in the pattern of cognitive impairments between juvenile and adult onset myotonic dystrophy type 1. *J. Clin. Neurosci.* 68 92–96. 10.1016/j.jocn.2019.07.029 31371188

[B366] Wozniak J. R. Mueller B. A. Bell C. J. Muetzel R. L. Lim K. O. Day J. W. (2013). Diffusion tensor imaging reveals widespread white matter abnormalities in children and adolescents with myotonic dystrophy type 1. *J. Neurol.* 260 1122–1131. 10.1007/s00415-012-6771-4 23192171 PMC3609908

[B367] Wozniak J. R. Mueller B. A. Lim K. O. Hemmy L. S. Day J. W. (2014). Tractography reveals diffuse white matter abnormalities in myotonic dystrophy type 1. *J. Neurol. Sci.* 341 73–78. 10.1016/j.jns.2014.04.005 24768314 PMC4042407

[B368] Wozniak J. R. Mueller B. A. Ward E. E. Lim K. O. Day J. W. (2011). White matter abnormalities and neurocognitive correlates in children and adolescents with myotonic dystrophy type 1: a diffusion tensor imaging study. *Neuromusc. Disord.* 21 89–96. 10.1016/j.nmd.2010.11.013 21169018 PMC3026055

[B369] Xia G. Ashizawa T. (2015). Dynamic changes of nuclear RNA foci in proliferating DM1 cells. *Histochem. Cell Biol.* 143 557–564. 10.1007/s00418-015-1315-5 25715678 PMC4439307

[B370] Xia G. Gao Y. Jin S. Subramony S. H. Terada N. Ranum L. P. (2015). Genome modification leads to phenotype reversal in human myotonic dystrophy type 1 induced pluripotent stem cell-derived neural stem cells. *Stem Cells* 33 1829–1838. 10.1002/stem.1970 25702800 PMC4441571

[B371] Xia G. Santostefano K. E. Goodwin M. Liu J. Subramony S. H. Swanson M. S. (2013). Generation of neural cells from DM1 induced pluripotent stem cells as cellular model for the study of central nervous system neuropathogenesis. *Cell. Reprogram.* 15 166–177. 10.1089/cell.2012.0086 23550732 PMC3616452

[B372] Yadava R. S. Foff E. P. Yu Q. Gladman J. T. Kim Y. K. Bhatt K. S. (2015). TWEAK/Fn14, a pathway and novel therapeutic target in myotonic dystrophy. *Hum. Mol. Genet.* 24 2035–2048. 10.1093/hmg/ddu617 25504044 PMC4355029

[B373] Yadava R. S. Yu Q. Mandal M. Rigo F. Bennett C. F. Mahadevan M. S. (2020). Systemic therapy in an RNA toxicity mouse model with an antisense oligonucleotide therapy targeting a non-CUG sequence within the DMPK 3′UTR RNA. *Hum. Mol. Genet.* 29 1440–1453. 10.1093/hmg/ddaa060 32242217 PMC7268549

[B374] Yamamoto H. Kokame K. Okuda T. Nakajo Y. Yanamoto H. Miyata T. (2011). NDRG4 protein-deficient mice exhibit spatial learning deficits and vulnerabilities to cerebral ischemia. *J. Biol. Chem.* 286 26158–26165. 10.1074/jbc.M111.256446 21636852 PMC3138246

[B375] Yamazaki Y. Matsubara T. Takahashi T. Kurashige T. Dohi E. Hiji M. (2011). Granulovacuolar degenerations appear in relation to hippocampal phosphorylated tau accumulation in various neurodegenerative disorders. *PLoS One* 6:e26996. 10.1371/journal.pone.0026996 22073234 PMC3207829

[B376] Yin H. Moulton H. M. Seow Y. Boyd C. Boutilier J. Iverson P. (2008). Cell-penetrating peptide-conjugated antisense oligonucleotides restore systemic muscle and cardiac dystrophin expression and function. *Hum. Mol. Genet.* 17 3909–3918. 10.1093/hmg/ddn293 18784278 PMC7108561

[B377] Zanigni S. Evangelisti S. Giannoccaro M. P. Oppi F. Poda R. Giorgio A. (2016). Relationship of white and gray matter abnormalities to clinical and genetic features in myotonic dystrophy type 1. *Neuroimage Clin.* 11 678–685. 10.1016/j.nicl.2016.04.012 27330968 PMC4900512

[B378] Zhang B. W. Cai H. F. Wei X. F. Sun J. J. Lan X. Y. Lei C. Z. (2016). miR-30-5p regulates muscle differentiation and alternative splicing of muscle-related genes by targeting MBNL. *Int. J. Mol. Sci.* 17:182. 10.3390/ijms17020182 26840300 PMC4783916

[B379] Zhang F. Bodycombe N. E. Haskell K. M. Sun Y. L. Wang E. T. Morris C. A. (2017). A flow cytometry-based screen identifies MBNL1 modulators that rescue splicing defects in myotonic dystrophy type I. *Hum. Mol. Genet.* 26 3056–3068. 10.1093/hmg/ddx190 28535287 PMC5886090

[B380] Zhang N. Bewick B. Xia G. Furling D. Ashizawa T. (2020). A CRISPR-Cas13a based strategy that tracks and degrades toxic RNA in myotonic dystrophy type 1. *Front. Genet.* 11:594576. 10.3389/fgene.2020.594576 33362853 PMC7758406

[B381] Zhang W. Liu H. Han K. Grabowski P. J. (2002). Region-specific alternative splicing in the nervous system: implications for regulation by the RNA-binding protein NAPOR. *RNA* 8 671–685. 10.1017/s1355838202027036 12022233 PMC1370287

[B382] Zhang W. Wang Y. Dong S. Choudhury R. Jin Y. Wang Z. (2014). Treatment of type 1 myotonic dystrophy by engineering site-specific RNA endonucleases that target (CUG)(n) repeats. *Mol. Ther.* 22 312–320. 10.1038/mt.2013.251 24196578 PMC3916045

[B383] Zorumski C. F. Izumi Y. (2012). NMDA receptors and metaplasticity: mechanisms and possible roles in neuropsychiatric disorders. *Neurosci. Biobehav. Rev.* 36 989–1000. 10.1016/j.neubiorev.2011.12.011 22230702 PMC3288588

